# MYC Binding Near Transcriptional End Sites Regulates Basal Gene Expression, Read‐Through Transcription, and Intragenic Contacts

**DOI:** 10.1002/advs.202414601

**Published:** 2025-05-30

**Authors:** Huabo Wang, Bingwei Ma, Taylor Stevens, Jessica Knapp, Jie Lu, Edward V. Prochownik

**Affiliations:** ^1^ Division of Hematology/Oncology UPMC Children's Hospital of Pittsburgh Pittsburgh PA 15201 USA; ^2^ Department of Colorectal Surgery The First Affiliated Hospital of USTC Division of Life Sciences and Medicine University of Science and Technology of China Hefei Anhui 230001 China; ^3^ The Department of Microbiology and Molecular Genetics UPMC The Hillman Cancer Center of UPMC The Pittsburgh Liver Research Center, UPMC Pittsburgh PA 15224 USA

**Keywords:** BRD4, chromatin, CTCF, DNA looping, enhancers, mediator, RNA polymerase II

## Abstract

The MYC oncoprotein regulates numerous genes involved in processes such as cell cycle control and mitochondrial and ribosomal structure and function. This requires heterodimerization with its partner, MAX, and binding to specific promoter and enhancer elements. Here, it is shown that MYC and MAX also bind near transcriptional end sites (TESs) of over one‐sixth of all annotated genes. These interactions are dose‐dependent, evolutionarily conserved, stabilize the normally short‐lived MYC protein and regulate expression both in concert with and independent of MYC's binding elsewhere. MYC's TES‐associated binding, occurring in coordination with other transcription factors, alters the chromatin landscape, increases nuclease susceptibility and alters transcriptional read‐through, particularly in response to certain stresses. MYC‐bound TESs can directly contact promoters and appear to fine‐tune gene expression in response to both physiologic and pathologic stimuli. Collectively, these findings support a previously unrecognized role for MYC in regulating transcription and its read‐through via direct intragenic contacts between TESs and promoters.

## Graphical abstract

MYC is a transcription factor (TF) that binds DNA near transcriptional start sites (TSSs) and within enhancer elements. Here, unappreciated sites of MYC binding in the vicinity of transcriptional end sites (TESs) of many genes in multiple cell types in association with numerous other TFs are described previously. TES‐associated MYC binding influences basal and read‐through transcription; facilitates direct TSS‐TES contacts and represents a novel form of gene regulation by this pervasive oncoprotein.

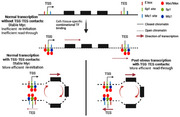

## 1 Introduction

The c‐MYC (MYC) bHLH‐ZIP oncoprotein transcription factor (TF) and its obligate bHLH‐ZIP heterodimerization partner MAX supervise the transcription of hundred‐thousands of genes involved in essential processes such as cell cycle progression, mitochondrial and ribosomal structure and function and translation.^[^ ^1^ ^]^ Sequence‐specific DNA binding by MYC/MAX heterodimers classically occurs at canonical “E boxes” that reside in proximal promoters.^[^ ^1^, ^2^ ^]^ This prompts the recruitment of histone acetylases, which modify and relax chromatin so as to facilitate subsequent entry by RNA polymerase II (RNAPII) and other transcriptional co‐factors. Together, these drive mRNA capping and overcome blocks to elongation that otherwise suppress the initiation and maintenance of robust levels of transcription.^[^ ^3^ ^]^ MAX can also associate with 6 additional bHLH‐ZIP TFs (MXD [1‐4], MNT, and MGA), collectively known as the “MXD” family.^[^ ^1^, ^4^ ^]^ Often expressed in ways that are tissue‐specific and developmentally‐dependent, MAX/MXD heterodimers compete for many of the sites occupied by MYC/MAX.^[^ ^1^, ^4^ ^]^ In doing so, they recruit histone de‐acetylases, which revert the adjacent chromatin to its original compacted and transcriptionally suppressed state.^[^ ^1^, ^4^ ^]^ Together, the above factors comprise the so‐called “MYC Network” that exerts sensitive, flexible, and integrative regulation and balance of MYC target gene expression in response to what are often rapidly changing and conflicting environmental and proliferative cues.^[^ ^1^, ^4^ ^]^ The importance of maintaining strict control among MYC Network members’ levels and functions is underscored by the embryonic lethality of body‐wide *Myc* or *Max* gene knockout (KO) in mice, by the accelerated aging phenotype that accompanies post‐natal *Myc* KO, by the association of MYC over‐expression with numerous naturally‐occurring human cancers, and by the development of cancer in mice driven by MYC deregulation.^[^ ^3^, ^5^ ^]^

MYC also negatively regulates gene expression. This is mediated indirectly by MYC‐MAX heterodimers interacting with and suppressing positively‐acting TFs such as MIZ1, SP1, and SP3, which normally bind sequence‐specific Initiator elements and GC‐rich SP1 sites, respectively, and, like E boxes, tend to lie in proximity to transcriptional start sites (TSSs).^[^ ^6^ ^]^

Previous studies of MYC‐mediated target gene regulation have focused on its associations with promoter‐proximal regions or enhancer elements. The locations of the latter sites cannot be predicted a priori; they are often located many kilobases upstream or downstream of a gene's coding region or may be embedded within its body^[^ ^2^, ^7^ ^]^ Here, however, we describe and characterize evolutionarily conserved and dynamic MYC and/or MAX binding in the vicinity of transcriptional end sites (TESs) of nearly one‐sixth of all annotated genes. Like TSS‐associated 5′‐end binding, MYC/MAX binding near TESs regulates target gene expression in ways that reflect the identities of the bound factors, their cooperation with MYC/MAX binding at other sites, and tissue‐specific preferences. TES‐associated MYC/MAX binding also tends to occur in genes whose functions differ from those lacking such binding. Finally, it contributes to both basal and stress‐induced transcriptional read‐through and the formation of intragenic contacts both with promoters and more distally located enhancers. Collectively, these studies reveal heretofore unrecognized forms of gene regulation mediated by unconventional MYC/MAX binding near TESs.

## 2 Results

### 2.1 MYC and/or MAX Frequently Bind in Proximity to TESs

**Figure 1:**
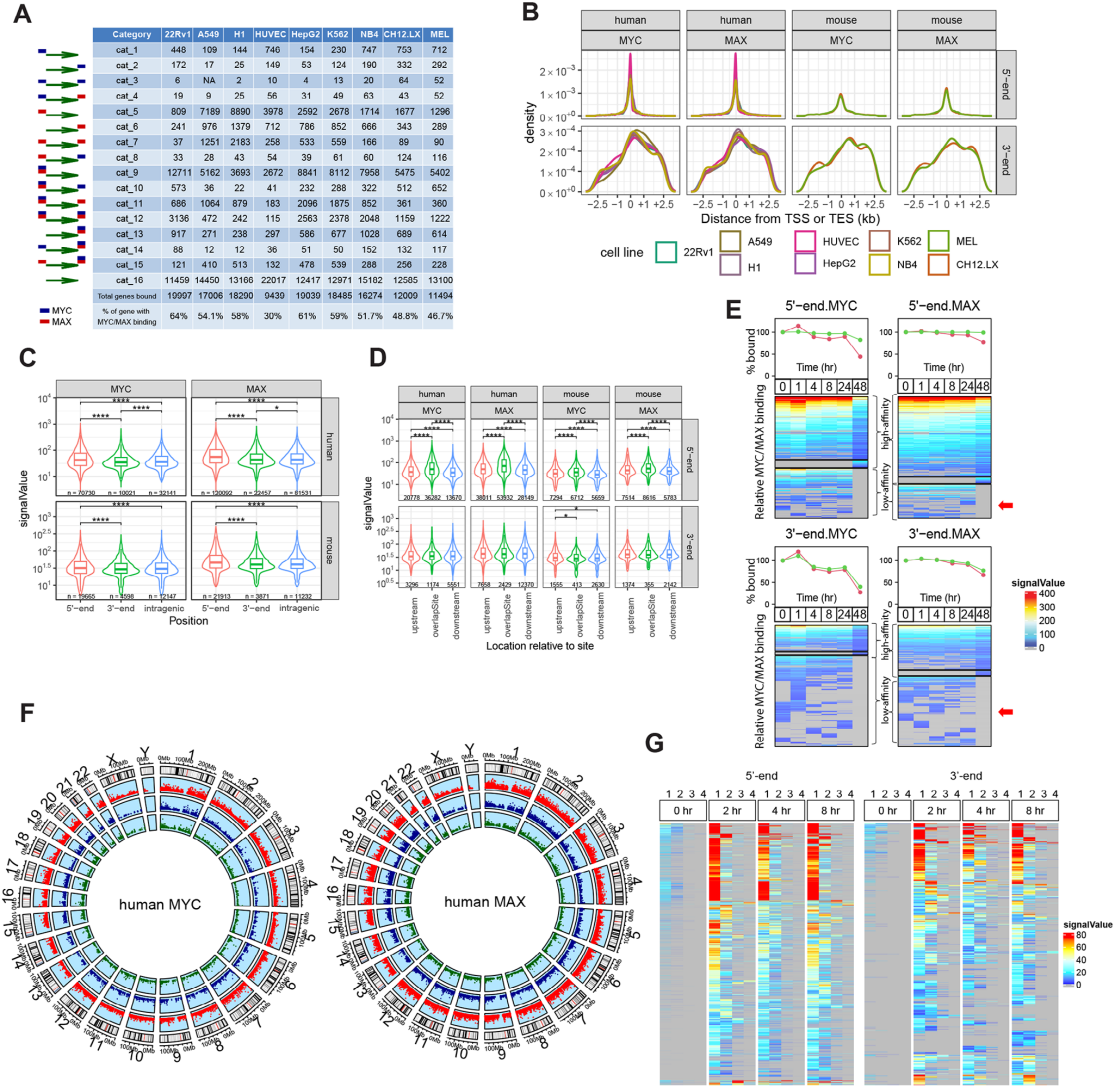
MYC and/or MAX binding site distribution and affinities in the vicinity of TSSs and TESs of human and murine genes. A) Frequency of MYC and MAX binding sites in proximity to TSSs and TESs of all annotated genes in the indicated human and murine cell lines. All possible configurations of MYC (blue) and/or MAX (red) binding are depicted at the left and are arbitrarily designated as categories (cat.) 1–15. Each column indicates the number of genes from each category that were identified in the referenced cell line. To simplify classification, no distinctions were made between genes with single or multiple binding sites (Figure S1, Supporting Information). B) Distribution of MYC and MAX binding sites relative to TSSs and TESs, which are defined as residing at position “0” within their respective genes. Some genes are repeated to allow for assignments to multiple TSSs and/or TESs (Figure S1, Supporting Information). Binding site distribution patterns in individual cell lines are shown in Figure S2 (Supporting Information). C) Mean binding signals ± 1 S.E. for MYC and MAX around TSSs, TESs, and intragenic regions for the genes depicted in A. Numbers below each violin plot indicate the actual number of binding peaks counted. Statistical comparisons were performed using unpaired *t*‐tests. Significance is denoted as follows: *: *p* < 0.05; ***: *p* < 0.001. D) Mean binding signals ± 1 S.E. for MYC and MAX whose peaks reside up to 2.5 kb upstream or downstream of TSSs and TESs. Statistical comparisons were performed using unpaired t‐tests. *: *p* < 0.05; ***: *p* < 0.001. E) Binding near TSSs and TESs stabilizes MYC. Upper portion of panels: 22Rv1 prostate cancer cells were exposed to MYC inhibitor MYCi975 (10 µm) for the indicated times.^[^ ^8^, ^9^ ^]^ MYC and MAX ChIPseq were then performed, and their binding signals around TSSs and TESs of all target genes were quantified. Green circles: percent of MYC or MAX remaining bound. Red circles: binding signal intensity at these sites relative to that at t = 0. Lower portion of panels: heat maps indicating the amounts of MYC or MAX remaining bound to individual genes with high‐ and low‐affinity MYC and/or MAX binding. Immuno‐blotting of whole cell lysates from the cells used in these ChIPseq studies had previously demonstrated near‐total depletion of MYC protein by 48 h.^[^ ^8b^ ^]^ Red arrows indicate groups of genes with low‐affinity sites that are selectively and completely depleted of MYC by 48 h. F) Circos plots indicating the chromosomal locations of annotated human genes that bind MYC and/or MAX only TSS‐associated regions (red dots), only at their TES‐associated regions (green dots), or at both ends (blue dots). The results were compiled from all 7 of the human cell lines shown in panel A and Table S1 (Supporting Information). Figures S3 and S4 (Supporting Information) show these sites in individual human cell lines. Figures S5 and S6 (Supporting Information) show Circos plots for individual murine cell lines. G) Time‐dependent binding of MYC and MAX at multiple TSS‐ and TES‐associated sites in murine B lymphocytes following exposure to LPS at “0 h”. All genes identified as containing more than one MYC binding site in the vicinity of TSSs and TESs at any time during LPS induction are depicted. The binding sites were identified at each point during this exposure and the binding signals (affinities) quantified as explained in E. Numbers at the top of each panel indicate the individual MYC binding sites that were identified as being closest to (1) and furthest from (4) the TSSs or TESs of their respective genes.

Using genome‐wide ChIPseq data sets compiled from 5 human and 2 murine cell lines from the ENCODE and GEO databases, we performed an unbiased search for MYC and MAX binding sites in all genes, whose 5′ and 3′‐ends were defined by the start and end sites of each of their annotated transcripts (**Figure** **1**A; Table S1, Supporting Information).^[^ ^8^ ^]^ To maximize the likelihood that binding locations were assigned to functionally meaningful sites, we considered only those with peaks residing within ± 2.5 kb of previously defined TSSs or TESs. Binding assignments also considered the binding of MYC and MAX in genes adjacent to one another and oriented head‐to‐head, head‐to‐tail, or tail‐to‐tail. When such sites resided within the above‐mentioned ±2.5 kb limit in both genes, they were assigned to both genes. To further simplify our analyses, those genes containing multiple binding sites within the regions of interest were included in the same categories as those with single sites (Figure S1, Supporting Information).

The number of genes represented by each category varied up to 2.1‐fold across cell lines (ex., 22Rv1 versus HUVEC). The near‐identical and tightly clustered 5′‐end binding patterns among all cell lines showed that 64.4‐83.5% of MYC and MAX binding sites resided within ±500 bp of TSSs and 79.0‐91.5% resided within ±1.0 kb (Figure 1B; Figure S2, Supporting Information). 3′‐end binding patterns of MYC and MAX were also quite similar among cell lines, although more broadly distributed, with 49.1‐56.9% of sites located within ± 1.0 kb of TESs. Binding of MYC and/or MAX was detected at the 3′‐ends of 17.5% of all annotated genes. When considering both TSS‐ and TES‐proximal binding sites, some configurations were more common than others, although this also varied among cell types. For example, ′category 9″ binding (MYC/MAX binding at 5′‐ends) was the most common configuration in 6 of the 9 cell lines and accounted for 20.2‐63.6% of all bound genes (Figure 1A). The least common categories (3 and 4), representing 5′‐MYC/3′‐MYC and 5′‐MYC/3′‐MAX binding, collectively comprised no more than 0.50% of all bound genes in any cell type. Other categories showed large differences in their representation among the cell lines. For example, category 7 (5′‐MAX/3′‐MAX) varied over >60‐fold range as a percentage of all bound genes and category 10 (5′‐MYC/MAX/3′‐MAX) varied by nearly 50‐fold. At both 5′‐ and 3′‐ends, the number of individual MYC and/or MAX binding peaks for the vast majority of genes in all cell lines (as determined by the presence of distinct individual ChIPseq peaks) was no greater than 3–4, with multiple sites being more frequent at 5′‐ends (Figure S1, Supporting Information). Collectively, these results indicated that different cell lines displayed distinct MYC/MAX gene binding profiles centered around TSSs and TESs. They also suggested that these patterns represent only snapshots of single conditions, points in time, and states of differentiation among specific cell types that almost certainly change in response to different intracellular and environmental cues, including the levels of expression of various MYC Network members *(vide infra)*.

To better clarify our analysis and to aid in distinguishing TES from TSS binding, we removed any ambiguous peaks that could be construed as both TES and TSS binding among closely spaced genes in head‐to‐tail orientation. Peak signal intensities of MYC and MAX binding around TSSs and TESs from the above cell lines were used to approximate each factor's accessibility to DNA and its binding affinities. On average, these signals were stronger at 5′‐ends although considerable overlap was documented (Figure 1C). They were also stronger for peaks in closest proximity to TSSs (Figure 1D). In contrast, MYC binding at 3′‐ends was equally strong irrespective of where it occurred relative to TESs whereas MAX binding was modestly stronger at upstream sites. By comparison, MYC and MAX binding affinities at TESs in both human and mouse genes were equal to or only modestly lower than the affinities at intergenic sites (>2.5 kb downstream of TSSs and >2.5 kb upstream of TESs) that likely reflected enhancer‐specific binding.

The above binding signals were also used to measure MYC and MAX displacement from their 5′‐ and 3′‐end sites in 22Rv1 prostate cancer cells following exposure to the potent and specific small molecule MYC inhibitor MYCi975 (Figure 1E).^[^ ^8b^ ^]^ This led to 4 observations. First, and consistent with the results shown in Figure 1C, the mean binding affinities for MYC and MAX around TESs in untreated cells were somewhat weaker than those around TSSs as estimated both by their basal binding signals and the rates at which they were diminished in response to MYCi975. Second, low‐affinity MYC and MAX binding, particularly at 3′‐ends, tended to be depleted within 24–48 h of MYCi975 exposure (Figure 1E). Third, despite a >90% reduction in total MYC levels as determined by whole cell immuno‐blotting,^[^ ^8b^ ^]^ significant amounts of MYC and MAX remained bound to higher affinity sites at both ends more than 48 h after exposure to MYCi975 (82.5% versus 39.8%, respectively, for MYC [P = 1.1 × 10^−61^] and 99.0% versus 76.9%, respectively, for MAX [P = 1.3 × 10^−12^]). Finally, MAX's displacement from both 5′‐ and 3′‐ends at 48 h was less than that of MYC's (P = 5.5 × 10^−21^ and P = 6.5 × 10^−35^, respectively). This could reflect MAX's predilection to re‐associate with MXD members and remain bound as MYC levels declined following exposure to MYCi975, as well as its intrinsically longer half‐life.^[^ ^1^, ^4^, ^8^ ^]^ The unexpected persistence of MYC binding indicated that its association with DNA markedly extends its half‐life well beyond the standardly accepted 15–30 min.^[^ ^10^ ^]^

The loss of MYC and MAX binding around high‐affinity TSSs and TESs closely paralleled the MYCi975 exposure time (Figure 1E). In contrast, low‐affinity binding site displacement kinetics were sometimes more complex. For example, binding was initially lost at some sites, only to reappear at later times and then disappear again by 24–48 h. In other cases, no binding was seen initially but paradoxically appeared after a period of exposure to MYCi975 only to subsequently vanish. These findings suggested that MYCi975 treatment initially altered the local chromatin environment in ways that allowed some binding sites, previously unoccupied by MYC and/or MAX, to become permissive for transient binding, even though overall MYC levels were in decline.^[^ ^8b^ ^]^

Although the combined data from the above human cell lines revealed no obvious preferences in the genomic locations of MYC or MAX binding at TSS‐ or TES‐associated sites (Figure 1F), some differences were noted among individual cell lines. For example, less frequent binding of MYC to TES‐associated sites was observed on chromosomes 4 and 13 in A549 cells and on chromosome 4 in HUVECs and H1 cells (Figure S3, Supporting Information). Similarly, less frequent binding of MYC to both 5′ and 3′ sites was noted on chromosome 13 in A549 cells and on chromosomes 8 and 13–15 in H1 cells. This type of segregation was less notable for MAX binding at TES‐associated sites, although a relative paucity was seen in A549 cells (chromosome 13) and HUVECs (chromosomes 4 and 13). A relative lack of gene binding MAX around both TSSs and TESs was also seen on chromosome 15 in HUVECs (Figure S4, Supporting Information). These findings support the idea that some of the gene categories shown in Figure 1A are distributed unequally across the genome and can sometimes be bound by MYC and/or MAX in tissue‐specific ways. Such unequal distribution was less evident across the murine genome, although it may have been due to a lower number of evaluable cell lines, particularly for MAX (Figures S5 and S6, Supporting Information).

Finally, we assessed ChIPseq results from *D. melanogaster* for dMYC and dMAX binding sites.^[^ ^8a^ ^]^ Because shorter regions of non‐coding inter‐genic DNA exist in the fly genome, more stringent criteria were used for the search. We therefore included only those genes that bound these factors within ± 1.0 kb of TSSs or TESs. As was true for humans and mice, many genes bound dMYC and/or dMAX near both TSSs and TESs (Figure S7, Supporting Information). Their more homogeneous chromosomal binding may have reflected the significantly smaller genome of the fly, as well as the extensive larval tissue heterogeneity. At the very least, our findings provide support for the notion that both MYC‐ and MAX‐associated TES binding have remained highly conserved over >700 million years of metazoan evolution.^[^ ^11^ ^]^

As mentioned above, some genes contain multiple TSS‐ and/or TES‐associated MYC binding sites (Figure S1, Supporting Information). To determine the order in which these bind MYC and their relative affinities, we again used ChIPseq data to catalog the kinetics of newly synthesized MYC binding following lipopolysaccharide (LPS)‐mediated activation of quiescent murine B cells.^[^ ^8a^ ^]^ Prior to LPS induction, and despite extremely low MYC protein levels in these cells, small numbers of presumably high‐affinity MYC binding peaks were nonetheless detected at both 5′‐ and 3′‐ends and tended to be those closest to TSSs or TESs (Figure 1G). Within 2 h of LPS induction, large increases in the intensities of these signals were observed, as were new signals at more distal sites. We assume these to be of lower affinity and/or to have been previously inaccessible. Thus, in response to MYC induction, the kinetics of binding site occupancy, the number of sites bound, and the order in which this progressed were very similar at 5′‐ and 3′‐ends. They largely reflected increasing MYC levels and the distance of binding sites from TSSs and TESs, with the highest affinity binding occurring almost exclusively at sites closest to these landmarks.

### 2.2 MYC Binding Around TESs Impacts Gene Expression and Transcriptional Read‐Through

**Figure 2:**
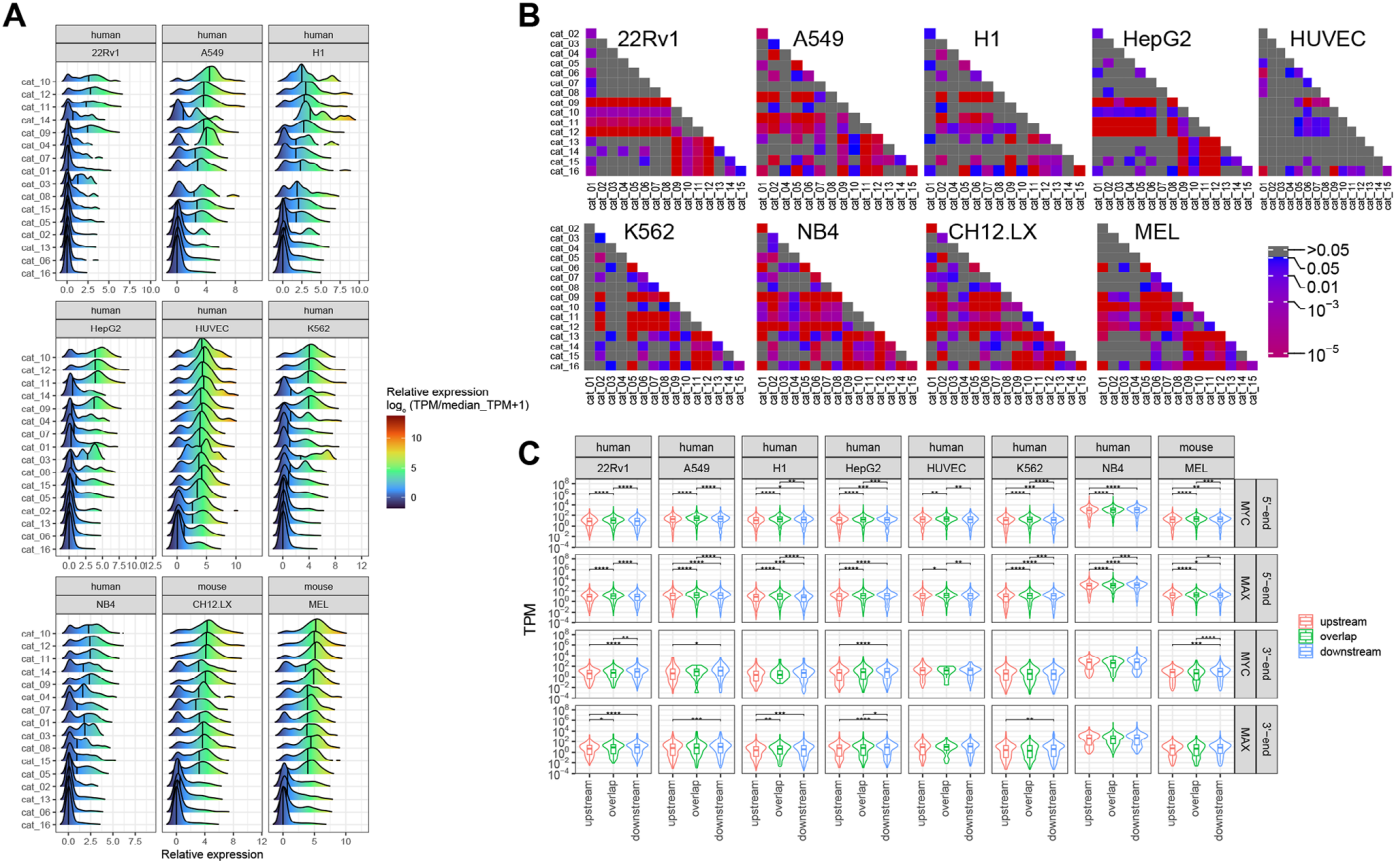
Gene expression is influenced by MYC and/or MAX binding to TESs of human genes. A) Ridge plots of RNAseq results from all 16 categories of genes are depicted in Figure 1A. Results are plotted to allow for both inter‐ and intra‐cell line comparisons. Blank areas indicate categories for which too few genes were available to allow for meaningful comparisons. B) The results from A were compared for significant differences in mean gene expression levels among all genes within the indicated categories. Statistical comparisons were performed using unpaired *t*‐tests. C) Gene expression levels among cell lines correlate with the location of MYC and/or MAX binding around TSSs and TESs. Using the data shown in Figure 1C,D and Figure S2 (Supporting Information), MYC and MAX binding peaks were assigned to sites that either overlapped TSSs or TESs or resided upstream or downstream of them. The mean transcript levels were then plotted for each group among the cell lines for which data were available. Statistical comparisons were performed using unpaired *t*‐tests. **: *p* <0.01; ***: *p* <0.001, ****: *p* <0.0001.

RNAseq results from the ENCODE and GEO databases^[^ ^8a^ ^]^ allowed transcript expression comparisons for the 16 gene categories depicted in Figure 1A. As expected, genes comprising categories 10, 11, and 12, all of which bound MYC/MAX at TSS‐proximal sites, were expressed at higher levels than unbound genes (categories 6,13, and 16) (**Figure** **2**A,B). However, within the former categories were also subsets of genes that were expressed at low levels in cell type‐specific ways. Conversely, the latter categories sometimes contained highly expressed gene subsets, particularly in HUVECs and MEL cells. Evidence that gene categories binding MYC and/or MAX only near TESs also differed in their expression relative to categories that did not (i.e., categories 2, 6, and 13 versus category 16) was seen in several cell lines (A549, H1, NB4, CH12.LX, and MEL). These findings indicated that the binding of MYC and MAX at 3′‐ends could impart variable degrees of generally positive control in a cell type‐specific manner when assessed agnostically across all expressed genes. The remarkable intercellular consistency of expression patterns across all 16 gene categories, despite differences in their gene content, suggested that the presence and location of MYC and/or MAX binding sites at both 5′‐ and 3′‐ends is a general and perhaps more fundamental aspect of gene expression that operates independently of tissue‐specific factors or specific gene identities.

Related to the way that MYC and MAX binding signal strengths reflected the distance of their peaks from TSSs and TESs (Figure 1C,D,G), so too was the expression of their bound genes. Those binding MYC and MAX at sites closest to or overlapping TSSs tended to be more highly expressed than those in which binding was more remote (Figure 2C). In contrast, only modestly higher levels of expression were associated with MYC and MAX binding downstream of TESs.

**Figure 3:**
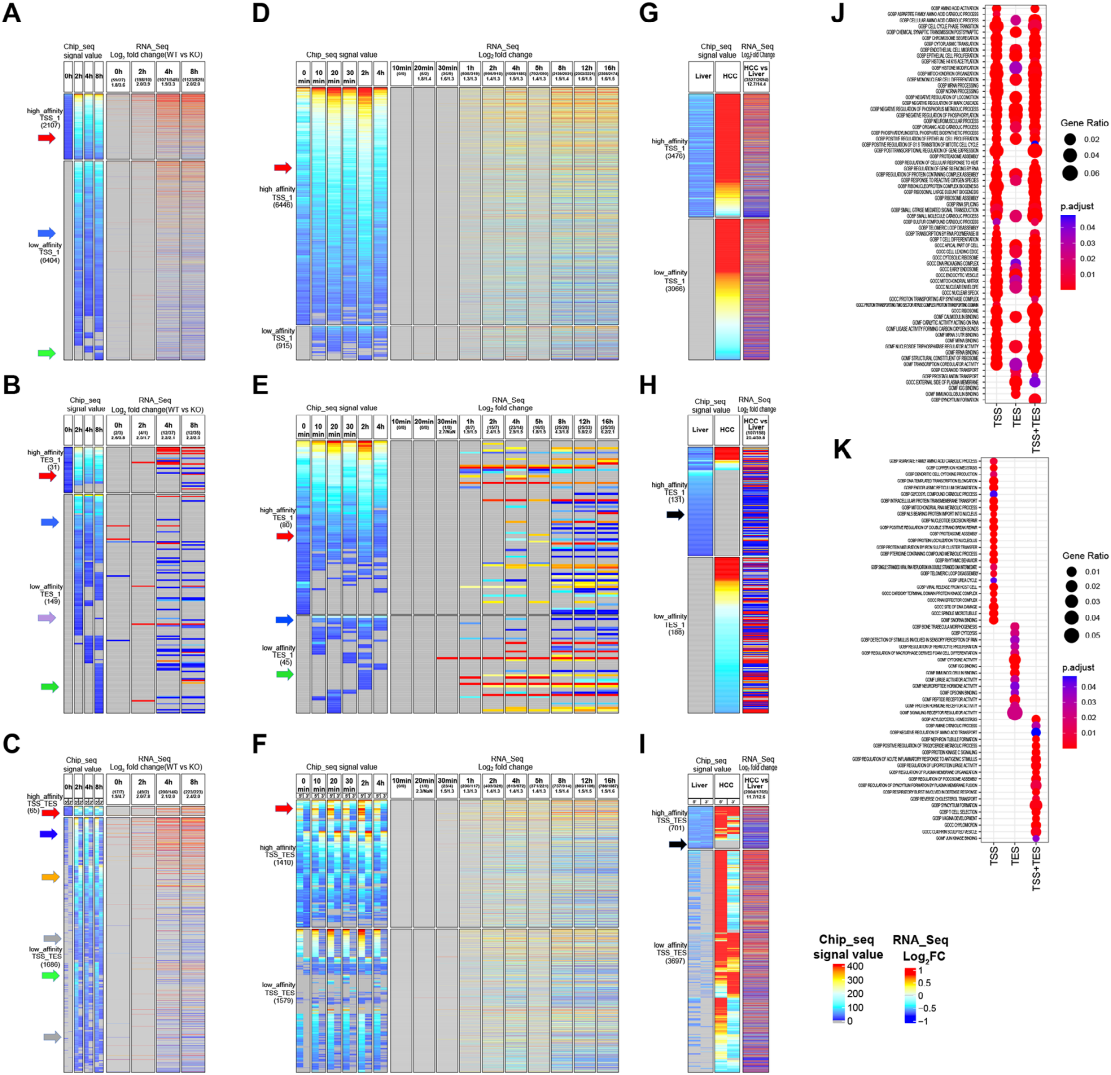
MYC binding near TSSs and/or TESs correlates with differences in gene expression and function. A–C) Quiescent wild‐type (WT) primary murine B lymphocytes treated with LPS.^[^ ^13^ ^]^ MYC ChIPseq and RNAseq were performed at the indicated times after LPS exposure and compared to the results obtained from LPS‐treated *Myc*‐/‐ (KO) B lymphocytes. Panel A: Chipseq and RNAseq results for genes binding MYC around TSSs only. Panel B: Chipseq and RNAseq data for gene binding MYC around TESs only. Panel C: Chipseq and RNAseq data for gene binding MYC at both TSS‐ and TES‐associated sites. Red arrows: examples of gene groups with high‐affinity MYC‐binding sites; blue arrows: examples of low‐affinity sites showing early occupancy by MYC in response to LPS; green arrows: examples of low affinity binding sites showing delayed occupancy by MYC; magenta arrows: examples of MYC binding sites transiently occupied by MYC; gray arrows: examples of MYC binding sites of about equal affinities around TSS and TES sites; orange arrows: example of more robust MYC binding at TES sites; black arrows: examples of high‐affinity sites failed to maintain MYC binding. Numbers above the heat maps indicate the total number of gene differences between the indicated comparisons and the mean fold‐up or down‐regulation. D–F) Logarithmically growing murine embryo fibroblasts harboring a conditional MYC‐estrogen receptor fusion protein (MycER) were treated with 4‐hydroxytamoxifen to activate MycER for the indicated periods of time. MYC ChIPseq and RNAseq were then performed to allow correlations between binding and expression for the subsets of genes that bound MYC only in proximity to TSSs D), TESs E), or both sites F). Arrows indicate similar gene sets described in panels A–C. G–I). HCCs were generated in mice by the hepatocyte‐specific and doxycycline‐regulatable induction of a human *MYC* transgene.^[^ ^5c^ ^]^ MYC ChIPseq and RNAseq were then performed to allow correlations between binding and expression for the subsets of genes that bound MYC only in proximity to TSSs G), to TESs H), or to both sites I). Black arrow in I: examples of high‐affinity MYC binding sites present only in normal liver. Other arrows indicate examples of genes whose behaviors are described in panels A–C. J) Over‐representation analysis from the MSigDB database's C5 GO collection. Genes that bound MYC exclusively at or around TSSs, exclusively at or around TESs, or at or around both TSSs and TESs were categorized into functional groups, the most significant of which are indicated. K) Functional categories of genes uniquely associated with binding of MYC to the sites indicated in J.

RNAseq and ChIPseq results from the LPS‐treated murine B cells described above were examined to allow correlations between MYC expression in response to a physiologic stimulus and its binding signals. Despite the high‐affinity binding of MYC to the previously mentioned minority of target genes prior to LPS exposure (Figure 1G), very little effect on their expression was seen relative to that in control *Myc‐/‐* B cells (**Figure** **3**A–C, red arrows). For these genes, as well as for many of those without any initial evidence of MYC binding, LPS treatment led to rapid MYC association that was largely completed within 2 h at both high‐ and low‐affinity sites, although some delayed binding was also observed (Figure 3A–C, blue and green arrows, respectively). Gene expression changes typically lagged DNA binding by 2–6 h, although exceptions were again noted. Seen in all 3 groups, but particularly prominent among genes with TES‐associated MYC binding only, were many examples in which MYC binding was transient despite more persistent changes in gene expression (Figure 3B magenta arrows). Comparisons of signal intensities again indicated, as noted previously, that MYC binding at 3′‐ends was of somewhat lower average affinity. This was best observed in genes with MYC binding at both ends, where high‐affinity binding prior to LPS induction was overwhelmingly confined to 5′‐ends (Figure 3C). Following LPS induction, however, a substantial number of these genes showed roughly equivalent levels of 5′‐ and 3′‐end MYC binding and some showed more robust binding at 3′‐ends (Figure 3C, gray and orange arrows, respectively).

MYC binding to TSS‐ and TES‐associated sites under “pathological” conditions of MYC over‐expression was examined in logarithmically growing immortalized murine embryo fibroblasts following high‐level induction of a *MYC*‐estrogen receptor (*Myc*ER) fusion transgene.^[^ ^12^ ^]^ Because these cells initially expressed substantial amounts of endogenous MYC, its binding to lower‐affinity target genes prior to MYC induction was more prominent and extensive than seen previously in quiescent B cells (Figure 3D–F, red arrows). As was true for B cells, many genes were associated with low‐affinity 3′‐end sites that bound MYC at different rates (Figure 3E, blue and green arrows).

Another example of target gene binding and gene expression changes in response to pathologic levels of MYC occurred in undifferentiated hepatocellular carcinomas (HCCs) induced by the conditional over‐expression of a human *MYC* transgene.^[^ ^5b,c^ ^]^ Despite its exceedingly low level expression in livers, MYC binding was nonetheless again detected at high‐affinity 5′‐ and 3′‐ends, whereas its binding to lower‐affinity sites was noted only in response to its over‐expression (Figure 3G–I). A small subset of high‐affinity sites also failed to maintain MYC binding despite the continued dysregulation of the associated target genes, most of which were down‐regulated (Figure 3H,I black arrow).

MYC target genes disproportionately participate in the maintenance of energy production, the support of ribosomal structure and function, the regulation of the cell cycle, and the response to and repair of genotoxic damage.^[^ ^1^, ^5^, ^14^ ^]^ A search of the C5 GO Collection from the MSigDB showed that genes binding MYC only at their 5′‐ends, only at their 3′‐ends or at both ends tended to share many of these and other functions (Figure 3J). However, certain non‐canonical functions were uniquely associated with the latter two groups. For example, genes with MYC binding restricted to 3′‐ends were more likely to be implicated in differentiation, proliferation, and cytokine/immune responses, whereas those with MYC binding at both ends were more likely to be involved in transport and membrane maintenance (Figure 3K). A similar analysis, performed upon genes with MYC‐associated TSS and/or TES classified as being of “high‐” or “low”‐affinity (Figure 3A–I) indicated that they too could be classified into distinct functional categories (Figure S8; Supplementary File S1, Supporting Information). Collectively, these results reveal that specific functional categories of genes are regulated not only by specific MYC binding at TESs but also by its affinity.

**Figure 4:**
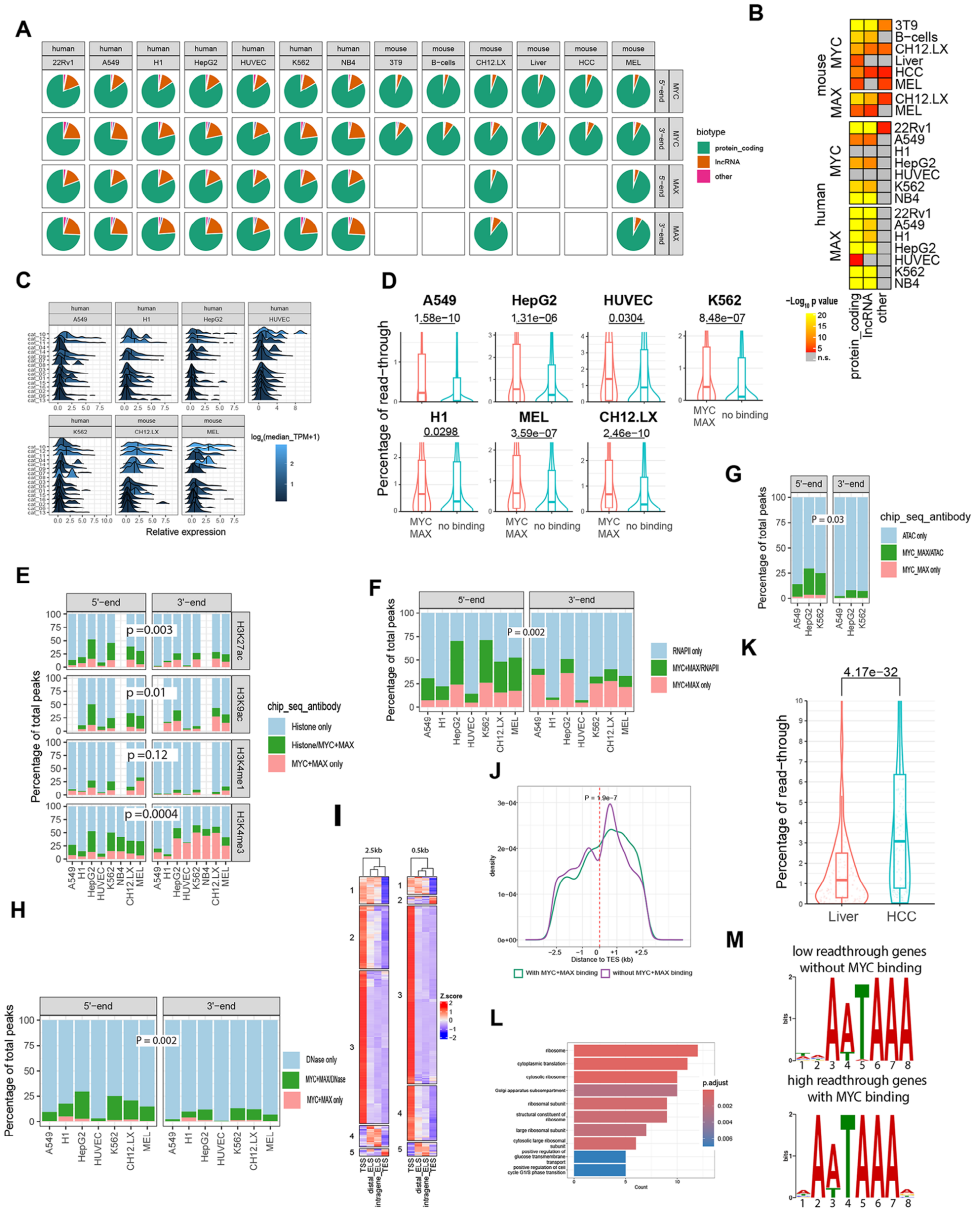
TES‐associated MYC and MAX binding is enriched at genes encoding lncRNAs, participates in chromatin re‐modeling, and alters transcriptional read‐through. A) Fractional distribution of MYC and MAX binding around TSSs and TESs of genes encoding proteins, lncRNAs, and other RNAs in the indicated cell lines. Blank boxes indicate data sets that were unavailable. B) Comparisons of the fraction of genes from A binding MYC/MAX near TESs versus TSSs in the indicated cell lines. Statistical significance was assessed using the Chi‐squared test. C) Ridge plots showing expression differences in lncRNAs for the 16 categories of MYC‐ and MAX‐binding genes (Figure 1A). Blank panels: too few genes were available to allow meaningful distribution patterns to be shown. D) MYC/MAX binding at and around TESs is associated with greater transcriptional read‐through. For expressed genes with or without evidence for TES‐associated MYC/MAX binding (Figure 1D), the ratio of transcripts containing sequences downstream of the TES to those originating from the last exon was determined in the indicated cell lines. Statistical significance was assessed using the Wilcoxon rank‐sum test. E) TSS‐ and TES‐associated MYC/MAX binding site peaks coincide with those for the transcriptionally permissive histone modifications H3K27ac, H3K9ac, H3K4me1, and H3K4me3. ChIPseq data for annotated genes containing TSS‐ and TES‐associated MYC/MAX binding sites within the domains demarcated by Figure 1B were examined for the indicated modified histones using ChIPseq results.^[^ ^8a^ ^]^ Each histogram shows the fraction of genes for which modified histone and/or MYC/MAX binding peaks were or were not coincident. F) TSS‐ and TES‐associated MYC/MAX binding site peaks coincide with those for RNAPII. ChIPseq studies were performed as described as in panel E. G) ATACseq results showing susceptibilities of genes binding MYC/MAX in the vicinity of TSSs and TESs to Tn5 transposase cutting. H) Susceptibilities to DNaseI digestion of genes binding MYC/MAX in the vicinity of TSSs and TESs. I) Binding frequencies of 1210 TFs and co‐factors (File S2, Supporting Information) to TSSs, TESs, and intragenic and distal enhancer‐like sequences (ELS) associated with known MYC‐binding human genes. The TSS‐ and TES‐associated boundaries employed in the search were either the same as those used to define MYC and MAX binding (i.e., ± 2.5 kb: left‐most heatmap) (Figure 1B) or were narrowed to ± 0.5 kb to minimize the inclusion of enhancer sequences (right‐most heatmap). Numbers to the left of the panels (1–5) indicate subsets of factors whose binding patterns allowed the 4 different regions to be distinguished from one another. J) Broader distribution of RNAPII around TESs that bind MYC and/or MAX than at those that do not. K) Fractional DoG transcription of 190 genes expressed in livers and MYC‐driven HCCs^[^ ^5b,c^ ^]^ showing higher levels of TES‐associated MYC/MAX binding in the latter. Statistical significance was assessed using the Wilcoxon rank‐sum test. L) Functional categories of the 190 genes shown in panel K as determined by IPA. M) HOMER plots showing consensus transcriptional termination/poly(A) sites in genes with the highest and lowest levels of DoG expression from K. In panels E–H and J, statistical comparisons were performed using unpaired *t*‐tests.

MYC and MAX also disproportionately bound at or near the TESs of genes encoding long non‐coding RNAs (lncRNAs) (**Figure** **4**A,B). In all cell lines examined, a greater proportion of lncRNA genes bound MYC and MAX at 3′‐ends than at 5′‐ends (human: 19% versus 14%, P = 5.40e‐61; mouse: 7% versus 4%, P = 1.62e‐164). Overall binding at both 5′‐ and 3′‐ends was higher in human cell lines than in mouse cell lines (5′ end 14% versus 4% P = 2.7e‐196; 3′ end 19% versus 7%, P = 2.2e‐96). In those cell lines for which RNAseq data were available, expression patterns of lncRNAs also differed among several of the 16 binding categories (for example, see categories 10, 12, and 14 in Figure 4C). The confinement of MYC and MAX binding around TESs (Figure 1B) suggested a role in mRNA termination. The absence or mutation of cleavage/poly A) signals, various cellular stresses, and neoplastic transformation are among the factors associated with selective increases in transcriptional read‐through efficiency of some genes at the expense of precise mRNA termination and polyadenylation.^[^ ^15^ ^]^ Not uncommonly, these “downstream of gene” (DoG) transcripts extend 50–100 kb beyond canonical TESs.^[^ ^15b,c^ ^]^ We used ENCODE strand‐specific RNAseq data to obtain ratios of DoG transcript expression relative to that encoded by each gene's closest upstream exon. In all cell lines, MYC/MAX binding near TESs was associated with higher fractional read through (Figure 4D). Thus, in addition to contributing to the physiologic and pathologic responses of certain transcripts, specific 3′‐end binding by MYC and MAX is also associated with higher average levels of DoG transcription.

TES‐associated MYC/MAX binding sites, particularly those participating in DoG transcription, might be predicted to possess a more relaxed and transcriptionally conducive local chromatin environment resembling that associated with promoters and enhancers.^[^ ^16^ ^]^ Indeed, ChIPseq results for acetylated and methylated histones H3K27‐ac, H3K9‐ac, H3K4‐me1, and H3K4‐me3 in the available cell lines from Table S1 (Supporting Information) showed that the binding signals for these transcriptionally permissive modifications around TSSs and TESs commonly overlapped those for MYC/MAX (Figure 4E). These results indicated that TES‐associated MYC/MAX binding sites coincide with more extensive regions of transcriptionally permissive histone modifications and read through. This was further supported by demonstrating that many of the above 3′‐end MYC/MAX binding sites also bound RNAPII and that the surrounding regions were susceptible to both ATAC seq and DNaseI digestion (Figure 4F–H).

The above‐described epigenetic modifications of the chromatin landscape recalled classical enhancer elements, which, like promoters, are also marked by numerous TFs, including MYC and RNAPII.^[^ ^16^, ^17^ ^]^ We therefore utilized information gathered from the ReMap2022 database to generate binding profiles for 1210 TFs and co‐factors that mapped to MYC‐bound TESs, TSSs, and both distal and intragenic enhancers (File S2, Supporting Information). These were compiled from 737 human cell lines and tissues and 8103 Chipseq data sets containing 182 million binding regions. Over a range of ±2.5 kb, each of the above 4 MYC‐bound regions showed distinct binding patterns for these factors that could be roughly categorized into at least 5 groups (Figure 4I, leftmost panel). Intragenic and distal enhancers, the most closely related with regard to their binding patterns, could barely be distinguished, whereas TSSs and TESs could be distinguished by groups 1,2,3, and 5. Although TES binding profiles were more closely related overall to those of enhancers, they could nonetheless be readily identified by groups 1, 2, 4, and 5. These differences persisted when coverage was more narrowly restricted to include only factor binding footprints residing within ±0.5 kb of TESs although the number of factors belonging to each of the groups was altered (Figure 4I: rightmost panel). Thus, the local environments of MYC‐associated TESs and enhancers, as defined by TF and co‐factor binding, were distinct from those of TESs. Finally, the distribution of RNAPII around MYC and/or MAX‐associated sites was significantly broader than it was around sites unassociated with MYC and/or MAX binding (Figure 4J).

Tumor‐affiliated DoG transcription is often generated by stresses arising from nutrient deficiencies, redox imbalance, hypoxia, and the deregulated expression of oncogenes, including MYC.^[^ ^15^, ^18^ ^]^ We therefore asked whether DoG transcription in MYC‐induced HCCs^[^ ^5b,c^ ^]^ changed relative to that of normal livers due to more MYC binding near TESs. From 11 normal livers and 15 HCCs from the GEO database, we identified 190 genes with significantly higher levels of TES‐associated MYC binding in the latter (Table S2, Supporting Information). Though not necessarily expressed at higher levels, these genes did demonstrate nearly 3‐fold higher mean fractional levels of DoG transcription (Figure 4K). This was similar to previously reported results in stressed fibroblasts despite the latter having been based upon the sequencing of nuclear RNA, which tends to be enriched for DoGs transcripts.^[^ ^15b^ ^]^ These 190 transcripts comprised 34% of all DoG differences between livers and HCCs (total 590) indicating that factors other than MYC and MAX likely contribute to read‐through transcription and/or that transient MYC binding can have long‐term effects on transcription (Figures 3A–F and 4D–H).^[^ ^15b^ ^]^ Unlike the >4800 DoG transcripts previously identified in stressed NIH3T3 cells, which reportedly did not comprise distinct functional categories,^[^ ^15b^ ^]^ the above 190 transcripts were enriched for those encoding ribosomal proteins and translation factors, which are major categories of MYC‐regulated mRNAs (Figure 4L).^[^ ^1^, ^5^, ^19^ ^]^ 35% of them were also previously shown to have higher levels of DoG expression in NIH3T3 murine fibroblasts subjected to oxidative stress.^[^ ^15b^ ^]^

One proposed explanation for why certain genes are susceptible to DoG transcription is that their 3′‐end cleavage/poly(A) sites deviate from the consensus and thus represent “weaker” transcriptional termination signals.^[^ ^15b^ ^]^ We therefore examined all genes with 3′‐end‐associated binding of MYC/MAX and above‐average DoG transcription levels from the cell lines and HCCs mentioned above and compiled consensus HOMER plots of presumptive cleavage/poly(A) signals residing within 100 bp upstream of their assigned TESs. When compared to genes from the same sources with below‐average DoG transcription levels and without 3′‐end MYC+MAX binding, we found little, if any, difference in these motifs (Figure 4M). Thus, DoG transcription and TES‐associated MYC/MAX binding do not reflect obvious non‐consensus or weaker cleavage/poly(A) signals.

### 2.3 MYC and MAX Recognize Similar DNA Motifs at 5′‐ and 3′‐Ends of their Target Genes

**Figure 5:**
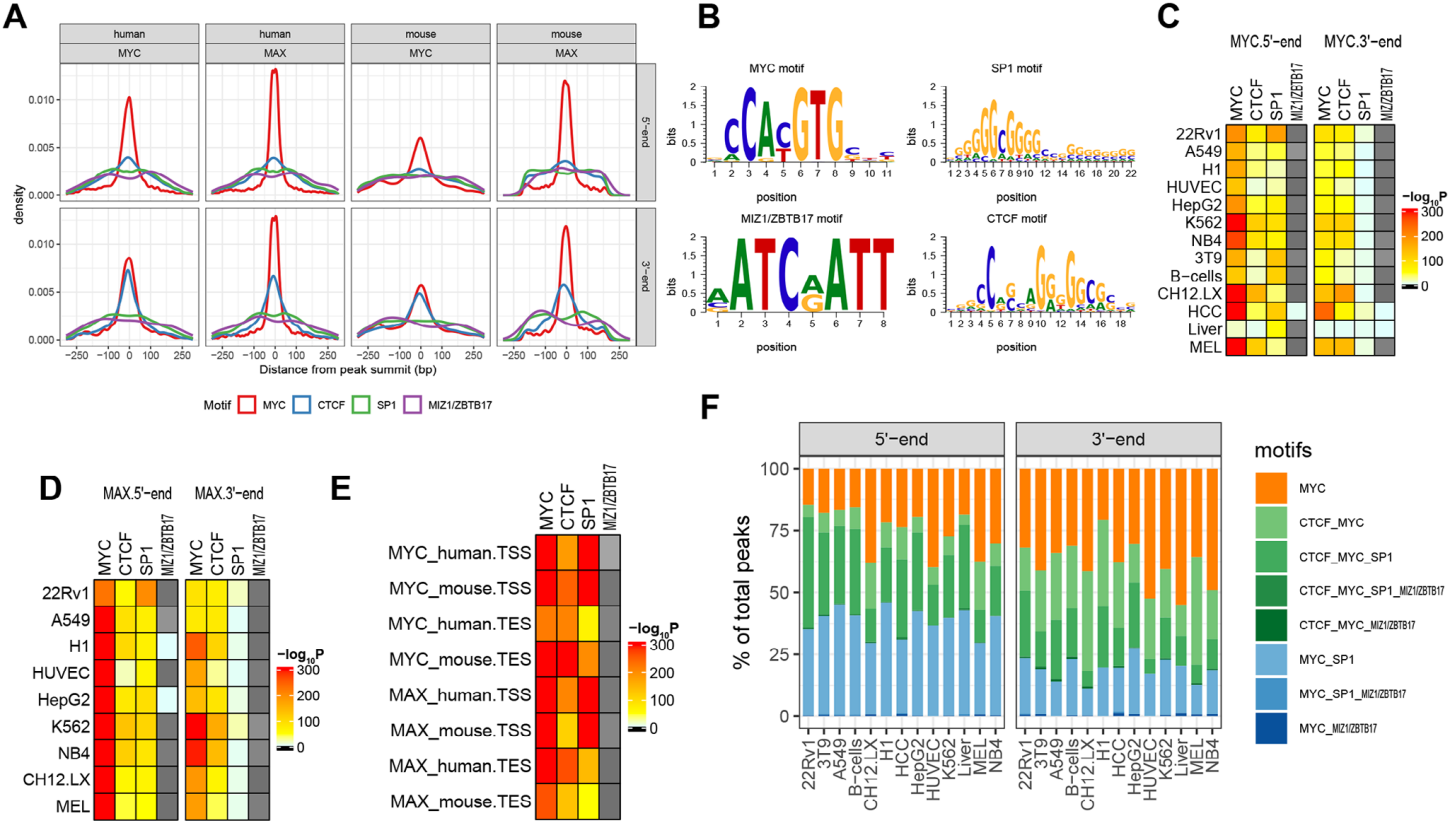
MYC and MAX binding around TSSs and TESs is associated with common DNA motifs. A) Distribution of the top 4 TF binding sites in relation to MYC binding peaks around TSSs and TESs of all annotated genes in the human and mouse cell lines depicted in Figure 1A. Position “0” is defined as the peak of the MYC footprint. B) HOMER plots showing consensus DNA binding motifs associated with ChIPseq peaks from panel A. C) P values for the results in A indicating the non‐random relationships of the indicated binding sites in relation to MYC ChIP signal peaks in TSS‐ and TES‐associated sites. D) P values for the results in A indicating the non‐random relationships of the indicated binding sites in relation to MAX ChIP signal peaks in TSS‐ and TES‐associated sites. E) Combined results from all cell lines shown in C and D. F) Association of MYC ChIP footprints with other binding domains. ChIPseq results from the cell lines depicted in A were combined. All DNA sequences in which peak binding corresponded to an E box motif were then searched as in A for consensus elements for the additional indicated TFs. On average, <25% of the regions associated with MYC footprints were associated exclusively with E boxes. In panels (C), (D), and (E), P values were generated using the FIMO tool from the MEME Suite, which assesses motif occurrences against a background model to determine statistical enrichment.

Although MYC/MAX heterodimers bind E boxes and drive transcription directly, their suppression of target genes is indirect and mediated via inhibitory interactions with MIZ1 and SP1/3.^[^ ^6^ ^]^ Using the same data sets employed for generating the heat map of TF binding sites at TSSs, TESs, and enhancers (Figure 4I), we performed motif enrichment using MEME Suite to compile a rank list of the consensus DNA binding motifs that overlapped the MYC/MAX footprints.^[^ ^20^ ^]^ Not unexpectedly, E boxes and SP1 sites were among the top 3 most prevalent motifs identified, with MIZ1 sites also being significantly enriched. The distribution of these sites was similar at 5′‐ and 3′‐ends of both human and mouse genes (**Figure** **5**A,B). The tight clustering of E boxes around MYC and MAX binding peaks likely reflected direct E box occupancy by these heterodimers whereas the more diffuse distribution of Sp1 and Miz1 sites indicated binding sites at which Sp1 and Miz1 binding were both independent of and associated with MYC and MAX.

Another top binding motif associated with 3′‐ends was that for the multi‐zinc‐finger protein “CCCTC binding factor” (CTCF). Originally described as a suppressor of MYC‐mediated transcription, CTCF also possesses more general roles in transcriptional insulation and repression, as well as chromatin looping between promoters and enhancers.^[^ ^16^, ^21^ ^]^ Some CTCF motifs contain loose approximations of E boxes embedded within their more complex consensus binding sequence (GCC^A^/_C_^C^/_T_CT^G^/_A_^G^/_C_),^[^ ^16d^ ^]^ although they do not appear to comprise even low‐affinity MYC/MAX binding sites.^[^ ^22^ ^]^ In general, CTCF motifs coincided more closely with those for MYC, particularly at TESs (Figure 5A). Thus, rather than being randomly distributed, all the above‐described motifs and their respective bound factors clustered around MYC binding peaks in close proximity to TSSs and TESs (Figure 5C–F).

### 2.4 MYC/MAX Binding at 5′‐ and 3′‐Ends of Genes is Associated with TSS‐TES Interactions

**Figure 6:**
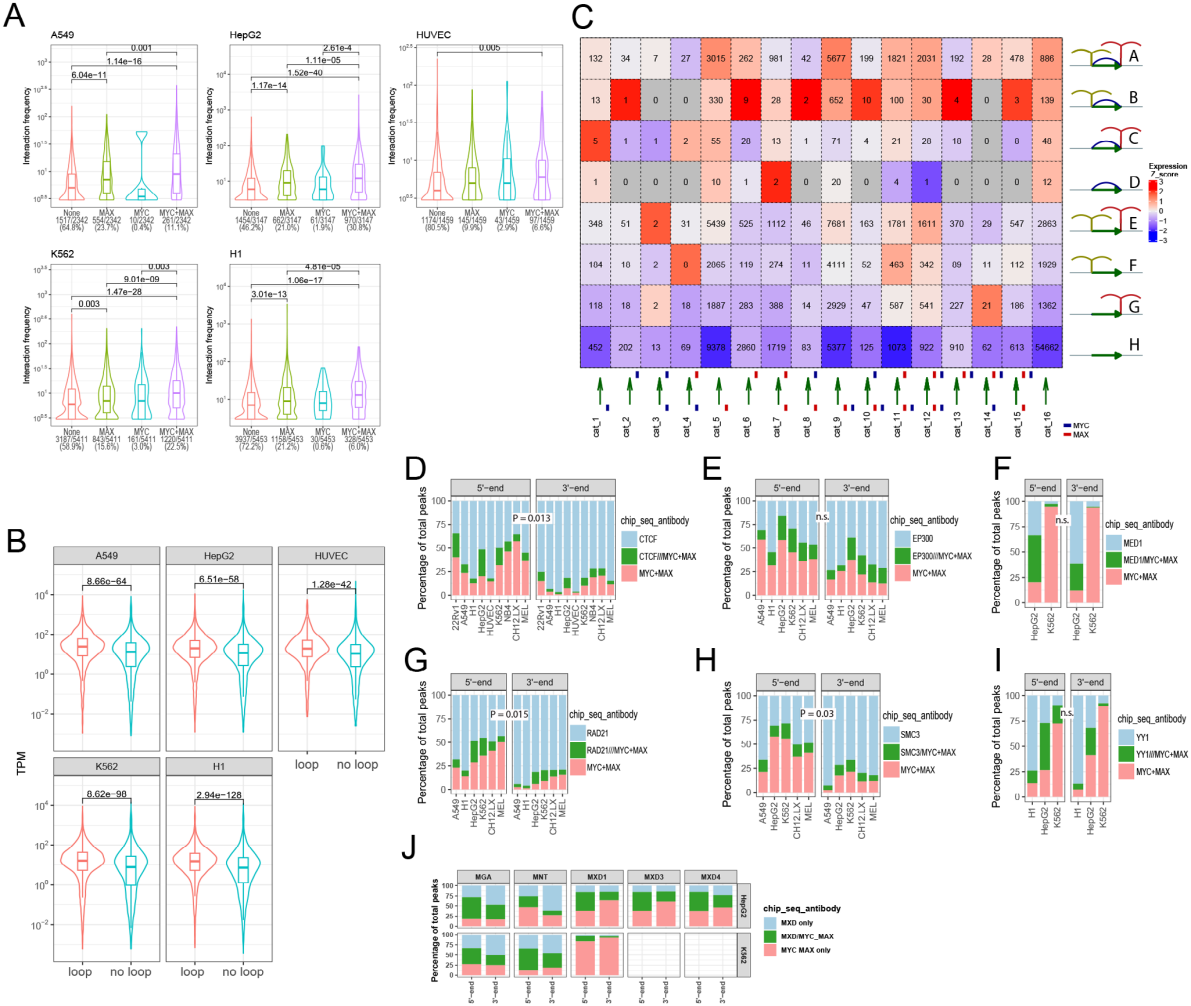
Intragenic contacts and expression are facilitated by TES‐proximal MYC and MAX binding. A) Gene interaction frequencies. For the indicated cell lines, all genes that bound RNAPII were divided into 4 groups based upon whether or not they also bound MYC and/or MAX at TES‐proximal sites. Using ChIA‐PET, TSS‐TES interaction (looping) frequencies were then determined for each of these groups. Numbers below each panel indicate the number and percentage of genes in each category. Statistical significance was assessed using the Wilcoxon rank‐sum test. B) Using RNAseq data for the cell lines shown in A, the expression of genes with or without TSS‐TES looping was determined. Statistical comparisons were performed using unpaired *t*‐tests. C) Combined gene expression heat maps and TSS‐TES interactions for the cell lines shown in A. All genes that bound RNAPII around TSSs and TESs were divided into the 16 MYC and/or MAX binding categories originally shown in Figure 1A. These were further divided into the 8 groups shown at the right A–H) according to types of TSS and TSS contacts that were identified based on ChIA‐PET analyses.^[^ ^8a^ ^]^ Blue curves shown in Groups A–D represent direct contacts between TSSs and TESs. Vertical ticks seen in all Groups except D and H indicate contacts between TSSs or TESs and additional intra‐ or extra‐genic sites. Numbers within each of the 128 squares of the heat map indicate the total number of genes identified among the cell lines. D) Binding of CTCF to regions ±2.5 kb of TSSs and TESs. P value for differences in the total binding of these factors is indicated between the 2 sets of histograms. E) Binding of p300 to the regions described in D. F) Binding of Mediator subunit MED1 to the regions described in D. G,H) Binding of cohesion complex subunits RAD21 and SMC3, respectively, to the regions described in D. I) Binding of YY1 to the regions described in D. J) Heterogeneity of select MAX binding partners at TSS‐ and TES‐proximal sites using ENCODE ChIPseq data for MYC, MAX and the indicated members of the MXD family in HepG2 and K562 cells. In panels D‐I, Statistical comparisons were performed using unpaired *t*‐tests.

Contacts between proximal promoters and remote enhancers, mediated by CTCF‐cohesin‐facilitated DNA looping, allow other TFs bound at these sites, including MYC, to interact directly.^[^ ^7^, ^23^ ^]^ Similar contacts involving TSSs and TESs are believed to allow RNAPII to more efficiently return to promoters upon completing each round of transcription.^[^ ^24^ ^]^ Using ChIA‐PET data from 5 evaluable cell lines in the ENCODE database,^[^ ^25^ ^]^ we assessed TSS‐TES contacts (looping) among genes that bound RNAPII around both TSSs and TESs and MYC and/or MAX around TESs. In all cases examined, TSS‐TES interactions were more abundant when MYC and MAX co‐bound at TES‐proximal sites (**Figure** **6**A). These genes were also expressed at significantly higher levels (Figure 6B).

For deeper insight into how MYC and MAX binding around TSSs and TESs influence DNA looping and transcription, all expressed genes in the above 5 cell lines were divided into the previously mentioned 16 categories (Figure 1A). ChIA‐PET results were then used to further sub‐classify these into 8 additional groups (A–H) based upon the types of contacts made by TSSs and TESs (Figure 6C). These included the TSS‐TES contacts discussed above (Groups A–D) as well as other intra‐ and extra‐genic interactions most likely involving enhancers. Combining these with the mean expression levels of these 128 individual gene sets yielded several observations. First, as already demonstrated (Figure 6B), gene Groups A–D were more highly expressed than those lacking TSS‐TES contacts (Groups E–H); second, expression among the 4 former groups was higher when their TSSs and TESs made additional intra‐ and extra‐genic contacts (groups A–C versus Group D). Third, MYC and MAX co‐binding around TSSs and TESs was associated with higher looping frequencies both at TSSs and elsewhere. For example, twice as many contacts (81.6%) were seen among category 12 genes, with MYC/MAX bound at both ends, than among category 16 genes, with no MYC or MAX binding at either (40.2%). Fourth, significant levels of looping between TSSs and TESs could still occur when MYC/MAX binding occupied only one end or when binding at both ends was incomplete, i.e., 60.1% in the case of category 9 genes, 56.2% in the case of category 13 genes and 81.1% in the case of category 10 genes. Collectively, these findings indicate that the influence exerted by MYC/MAX binding on gene expression is highly correlated with its TSS‐TES contacts. However, this appears to involve additional and likely complex interactions with other TFs as well as intra‐ and extra‐genic enhancers.

In addition to being enriched for RNAPII and CTCF, points of contact between promoters and enhancers also commonly contain the CTCF‐cohesion complex, the EP300 transcriptional activator/acetyltransferase, the multi‐subunit Mediator complex, and the bifunctional transcription factor YY1.^[^ ^7^, ^26^ ^]^ Again, using data from the ENCODE database for all or some of the cell lines (Table S1, Supporting Information)^[^ ^8a^ ^]^ we mapped the binding sites for these factors or their key subunits. Although somewhat less frequent at 3′‐ends than at 5′‐ends, these either directly overlapped MYC/MAX binding signal peaks or resided in close proximity to them (Figure 6D–I). Thus, the protein complexes associated with MYC/MAX at sites of contact between TSS‐ and TES‐containing regions resemble but are distinct from those associated with the points of contact between TSSs and enhancers.

Whether MYC/MAX binding around TESs allows genes to engage in DoG transcription or direct TSS contacts might be further influenced by heterodimers comprised of MAX and members of the MXD family, which negate the positive transcriptional activity of MYC.^[^ ^1^, ^4^ ^]^ Using available ChIPseq results from ENCODE for these factors that were reported for human HepG2 HCC cells and K562 chronic myelogenous leukemia cells, we found evidence for their binding to regions around both TSSs and TESs (Figure 6J). In some cases, their footprints were indistinguishable from those formed by MYC/MAX heterodimers, suggesting that different heterodimer combinations were competing for binding to the same sites.

### 2.5 Loss of TES‐Associated MYC/MAX Binding Alters Gene Expression, Basal and Stress‐Induced DoG Transcription and TSS‐TES Contacts

**Figure 7:**
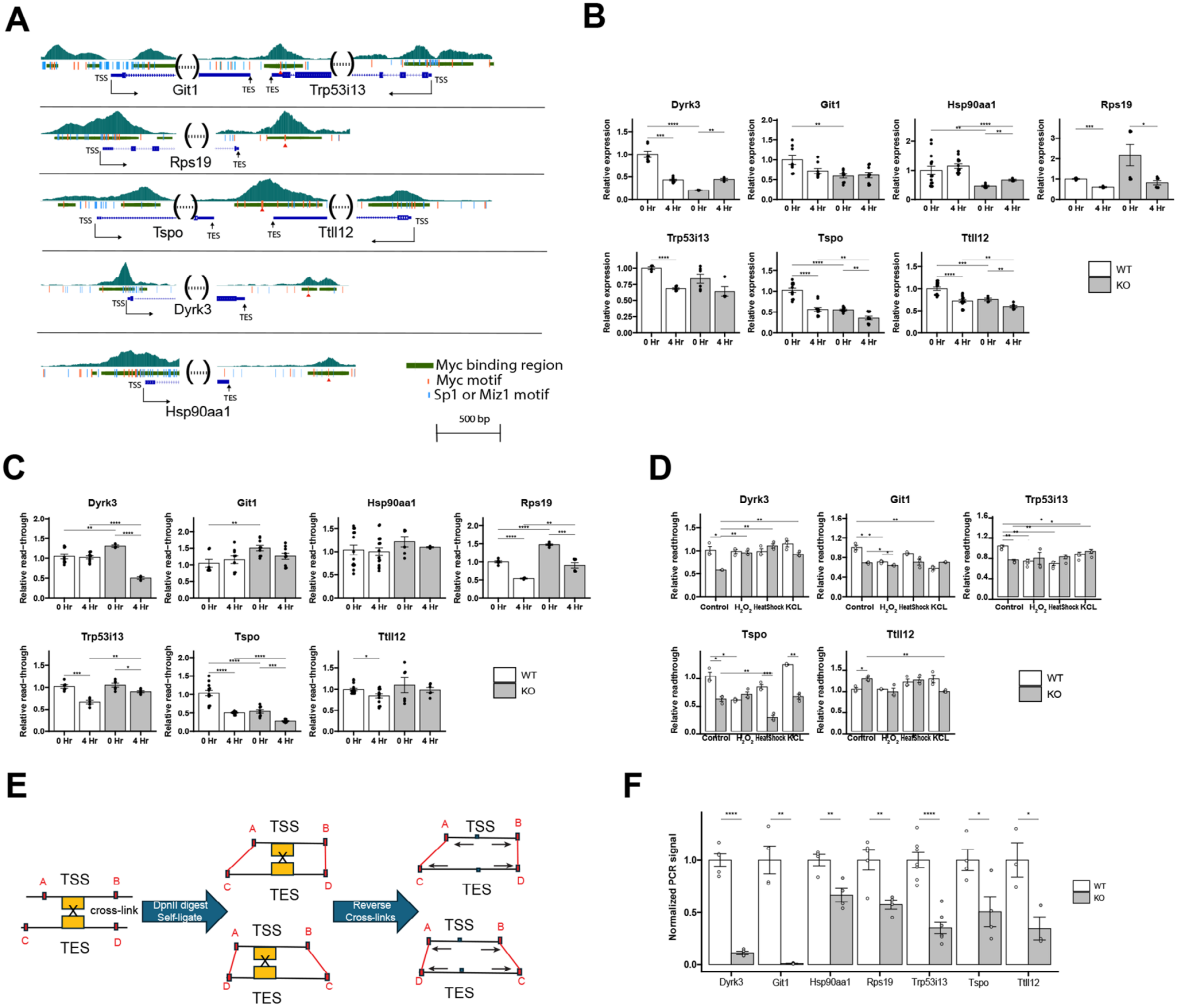
Loss of TES‐proximal E boxes impacts total and read‐through transcription and TSS‐TES contacts. A) Cartoons of the indicated 7 genes showing the locations of TSS‐ and TES‐associated MYC binding sites in immortalized murine fibroblasts along with consensus E boxes and Sp1 and Miz1 sites.^[^ ^8a^ ^]^ Note that the *Git1* and *Trpp53i13* and *Tspo* and *Ttll12* genes are arranged in tail‐tail orientation and bind MYC/MAX at a single site <2.5 kb from each gene's TES. Orange triangles denote the sites of E boxes that were mutated for subsequent studies. B) The indicated NIH3T3 fibroblast lines containing WT or mutant TES‐associated E boxes were maintained for 24 h in medium containing 0.1% FBS. One set of cells was then harvested at this point (time 0). Fresh medium containing 10% FBS was added to the remaining cells, which were harvested 4 h later. Transcript levels were determined in each set by qRT‐PCR using the primers indicated in Table S4 (Supporting Information) and normalized to those of transcripts for a control gene (TBP) as previously described.^[^ ^28^ ^]^ C) Basal read‐through transcription is impacted by E‐box mutagenesis. Total RNAs were directionally reverse‐transcribed using gene‐specific forward primers, followed by qPCR. All primers used are listed in Table S4 (Supporting Information). D) Changes in DoG transcription following a 4 h exposure to heat shock (45 °C), KCl (200 mm) or H_2_O_2_ (0.2 mm). DoG transcription was quantified as described in C. Control cells were grown under standard log‐phase conditions. E) General strategy for modified 3D chromatin conformation capture in WT and KO murine fibroblasts. The approach was designed to identify interactions between TSSs and TESs regardless of their relative orientations as indicated in the upper and lower portions of the diagram. Black lines indicate the gene body. Step 1: formaldehyde cross‐linking. Red boxes A–D indicate Dpn1 sites in closest proximity to TSSs, TESs, and MYC/MAX binding sites (orange boxes). Step 2: Cross‐linked chromatin digested with DpnII and self‐ligated (red lines). Step 3: Cross‐link reversal, SDS‐proteinase K digestion, and phenol‐chloroform extraction. Step 4: PCR reactions, with primers indicated by black arrows, designed to amplify fragments of defined sizes across all 4 possible ligated sites (Figure S10, Supporting Information). Correct PCR products were confirmed based on their size prior to and following DpnII digestion and amplicon sequencing. F) Quantification of TSS‐TES interactions. Using the primers shown in Figure S10 and Table S5 (Supporting Information), qPCR was performed on the indicated genes in WT and KO fibroblasts following formaldehyde cross‐linking, DpnII digestion, re‐ligation, and reversal of cross‐links as depicted in E. Results show the average of triplicate samples ± 1 S.E. after DNA inputs were adjusted using a set of PCR primers that amplified a region of the *Mlx* gene. In some cases, PCR products from WT cells longer than those indicated were found to arise as a result of protection of one of the DpnII sites (panel E) by MYC/MAX. Instead, digestion occurred at the immediately adjacent site. In panels B–D and F, Statistical comparisons were performed using unpaired t‐tests. *: *p* < 0.05; **: *p* < 0.01; ***: *p* < 0.001; ****: *p* < 0.0001.

Many cell types contain genes with high levels of MYC/MAX binding to TSS‐ and TES‐associated E boxes and robust DoG transcription (Figure 4D).^[^ ^8^, ^15^ ^]^ We identified 7 such genes in immortalized murine fibroblasts^[^ ^8a^ ^]^ and then used a Crispr/Cas9‐based approach to delete/mutate their consensus TES‐associated E boxes (**Figure** **7**A; Figure S9 and Table S3, Supporting Information). We then asked how this impacted transcript levels during serum‐starvation‐mediated quiescence (0.1% FBS x 24 h: “MYC‐low”) and the subsequent response to a brief period of serum‐stimulated endogenous MYC induction (10% FBS x 4 h: “MYC‐high”).^[^ ^27^ ^]^ Relative to wild‐type (WT) cells, transcript levels of 5 of the 7 genes were significantly reduced in MYC‐low KO cells indicating that even minimal levels of TES‐associated MYC binding are sufficient to maintain basal gene expression (Figure 7B). In contrast to this positive effect, most of the genes’ transcripts were down‐regulated in MYC‐high WT cells whereas their expression in MYC‐high KO cells was either less affected by serum or, in 2 cases, up‐regulated. Thus, for the limited number of genes examined, MYC binding to TES‐proximal E boxes appeared to serve 2 purposes. First, it positively maintained gene expression when endogenous MYC levels were low, as occurs during quiescence. Second, it determined both the direction and the magnitude of the gene's response during periods of transiently high MYC expression as occurs after a growth‐promoting stimulus.

Under MYC‐low conditions, DoG transcription of 3 genes (*Dyrk3, Git1*, and *Rps19*) in KO cells was increased, implying that MYC binding near TESs normally suppresses this process (Figure 7C and Table S4, Supporting Information). In contrast, the opposite effect was observed with the *Tspo* gene and no effects on DoG transcription were observed with *Hsp90aa1, Trp53i13*, or *Ttll12*. In response to serum stimulation, DoG transcription in KO cells was decreased for 2 genes (*Dyrk3* and *Tspo*), increased for 2 genes (*Rps19* and *Trp53ai13*) and unchanged for the remaining 3 genes (*Git1, Hsp90aa1*, and *Ttll12*). Thus, as was true with overall transcript expression, DoG responses in quiescent and serum‐stimulated cells, and their regulation by TES‐proximal MYC/MAX binding, were as varied as they were in genes that are driven solely by MYC binding near TSSs.^[^ ^1^, ^2^, ^5^, ^6^ ^]^

DoG transcription can also be impacted by other stresses such as heat and osmotic shock and reactive oxygen species, albeit in gene‐specific ways.^[^ ^15b,c^ ^]^ To determine whether TES‐associated E‐box loss affected these responses, 5 of the above cell lines were subjected to each of these 3 stresses for 4 h during log‐phase growth after which strand‐specific qRT‐PCR was performed to quantify DoG transcript levels (Table S4, Supporting Information). In WT cells, 3 genes (*Git1, Trp53l13*, and *Tspo*) were affected by at least one stress condition, although DoG transcription was reduced rather than increased (Figure 7D). In KO cells, DoG transcription was generally lower under control conditions, which largely replicated the results of serum‐stimulated MYC induction. The responses of KO cells were both gene‐ and stress‐specific and were not always decreased as they were in WT cells. Thus, TES‐associated MYC binding can impact both the directionality, extent and specificity of stress‐induced DoG transcription in highly gene‐specific ways.

The above‐noted changes in total and DoG transcription could have been facilitated via the formation of chromatin loops between MYC/MAX‐bound TESs and TSSs in a manner analogous to that of promoter‐enhancer contacts.^[^ ^7^, ^24^, ^26^, ^29^ ^]^ To test this, we performed a modified and focused version of 3D chromatin conformation capture in which DNA bound by MYC/MAX was chemically cross‐linked in WT and KO cell lines, digested with DpnII, and then self‐ligated prior to reversing the protein‐DNA cross‐links (Figure 7E). DpnII site junctions, in closest proximity to TSSs and TESs, were then amplified using PCR primer sets that flanked each of the 4 potential points of contact formed upon DpnII site re‐ligation (Figure S10, Supporting Information). The results of these studies showed that KO cells contained many fewer such re‐ligated sites (Figure 7F). Thus, just as was true for basal and DoG transcription, chromatin looping and direct contact between TSSs and TESs were facilitated and maintained by the presence of MYC binding at the latter sites.

### 2.6 MYC Binding at Functionally Ambiguous Sites can Simultaneously Co‐Regulate Closely‐Spaced, Head‐To‐Tail‐Orientated Genes

**Figure 8:**
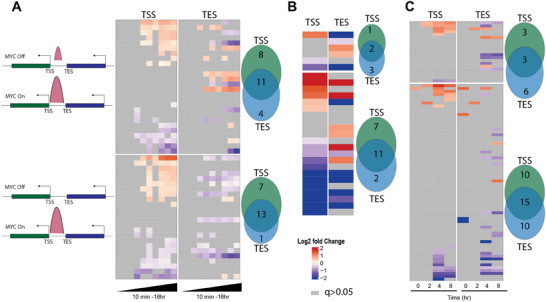
MYC binding at functionally ambiguous intergenic regions can regulate one or both of its neighboring genes. Transcriptomic profiling for gene pairs arranged in head‐to‐tail orientation and with MYC binding detected only in the intergenic region, as shown in the cartoons. Note that each heat map is divided into 4 quadrants, indicating the relative expression of closely‐spaced genes whose intergenic MYC binding sites could be assigned to either the TSS or TES of their respective gene pair. The upper left and right quadrants contain genes with high‐affinity intergenic MYC binding detected prior to MYC induction that was increased even further afterwards, as indicated by the adjacent cartoon. The lower left and right quadrants contain genes with lower affinity intergenic MYC binding that were only detected in response to MYC induction. The upper and lower left quadrants show the expression of genes downstream of MYC‐associated TSSs, whereas the upper and lower right quadrants show the expression of genes upstream of the TESs associated with the same MYC binding sites. At no time prior to or after MYC induction were any other MYC binding sites detected in the vicinity of these genes. Numbers to the right of each heat map indicate the total number of gene dyads included in its upper (high affinity sites) and lower segments (low affinity sites). A) Expression profiles of gene pairs in log‐phase murine fibroblasts prior to and after MycER activation for times ranging from 10 min to 16 h. The relative expression of all genes is normalized to their baseline level observed prior to MycER activation. B) Expression profiles of the gene pairs in murine HCCs originating in response to doxycycline‐regulated MYC induction and normalized to control livers. C) Expression profiles of gene pairs in response to LPS treatment of quiescent B cells and normalized to those of *Myc‐/‐* B cells treated in the same manner.

As mentioned earlier, many head‐to‐tail oriented genes are spaced closely enough such that MYC and/or MAX binding within their short intergenic regions cannot be unambiguously assigned functionally to the TSS of one gene or the TES of the other. We investigated this in greater detail in the previously described murine fibroblasts and livers in which high levels of MYC target gene binding and expression could be conditionally achieved by MycER activation and doxycycline induction, respectively and LPS‐stimulated murine B cells (Figure 3). Importantly, we considered those gene pairs that bound MYC only in the intragenic region, either prior to or following its induction. The genes under study thus resembled those depicted by categories 1,2,9, and 13 shown in Figure 1A. The exclusion of genes with MYC binding anywhere other than within these intergenic regions ensured that any expression changes in either member of the gene pair were likely to be the result of MYC or MYC/MAX binding only at these solitary proximal sites. We found striking similarities among the 3 cell and tissue types tested. In each case, gene expression profiles could be categorized into 3 distinct groups when assuming that regulation by MYC was mediated by the TSS element in a classical promoter‐specific manner (**Figure** **8**A–C; File S3, Supporting Information). The genes downstream of these sites showed either positive regulation, no regulation, or negative regulation in response to MYC induction. A similar set of categories was observed when we assigned functionality to the TES sites. In these cases, transcripts upstream of these sites were also regulated in the same manner. Although by no means uniform, certain groups of genes, most notably negative MYC targets associated with lower affinity binding, tended to show a coordinated response between genes located upstream and downstream of the intergenic binding site (lower left portions of heat maps in each panel). Collectively, these findings indicated that a single intergenic MYC binding site, in close proximity to the TSS and TES of an adjacent gene pair, can in many cases simultaneously regulate both genes.

## 3 Discussion

MYC and other TFs are traditionally portrayed as interacting with consensus DNA elements located in proximal promoters or enhancers, with the latter being classified as either intra‐ or extra‐genic.^[^ ^30^ ^]^ The contacts between these sites occur via chromatin looping and are mediated by various post‐translational histone modifications and protein‐protein interactions.^[^ ^7^, ^16^, ^17^, ^26^, ^30^ ^]^ These facilitate access by more general components of the transcriptional machinery, such as RNAPII, Mediator, and their co‐factors that additively or synergistically promote mRNA initiation, capping, the reversal of pausing, and elongation.^[^ ^3^, ^14^, ^30^ ^]^ Relying on ENCODE data,^[^ ^8a^ ^]^ we have identified heretofore unreported binding of MYC and/or MAX to the 3′‐ends of over one‐sixth of all annotated genes, with the vast majority of this binding centering around TESs (Figure 1A,B; Figure S2, Supporting Information). Its conservation over >700 million years of evolution thus precedes the bilaterian divergence and suggests important functional roles (Figure S7, Supporting Information).^[^ ^11^ ^]^ Typically, it involves co‐binding by both MAX and MYC, although single‐factor binding is not uncommon (Figure 1A). MAX binding in MYC's absence is more frequent and probably reflects its additional MYC‐independent interactions with MXD members (Figure 6J).^[^ ^1b,d^ ^]^ The fact that some sites appear to simultaneously bind both MYC/MAX and MXD/MAX heterodimers concurrently likely reflects cell‐to‐cell differences in the levels of these various factors and competition among them for occupancy of these sites. The observation that solitary MYC binding was 5 times less frequent is consistent with the fact that both its direct binding to DNA and its inhibitory interactions with positively‐acting TFs such as Miz1 and Sp1/3, occur in association with MAX.^[^ ^1^, ^6^ ^]^ Overall, binding by MYC and/or Max at either or both 5′‐ and 3′‐end of genes as defined here accounted for an average of 52.5% of all annotated genes (range 30.0‐63.6%) across the 9 human and murine cell lines examined in this study (Figure 1A).

Initial comparisons of MYC and MAX binding revealed both similarities and differences with regard to 5′‐ and 3′‐end binding profiles. For example, both factors’ binding centered around TSSs and TESs, although latter sites’ footprints were broader (Figure 1 B; Figure S2, Supporting Information). Both high‐ and low‐affinity binding sites were observed with the latter being somewhat more prominent at 3′‐ends (Figure 1E,G). In response to both physiologic and pathologic levels of MYC induction, changes in gene expression occurred as soon as 2 h after MYC/MAX binding and in genes with multiple binding sites, those residing closest to TSSs and TESs tended to be of higher affinity and were among the first to be occupied (Figures 1G and 3A–I). Collectively, these findings indicate that MYC and MAX binding sites around TSSs and TESs have evolved and behave similarly with respect to their spatial organization, binding affinities, and effects on gene expression in response to changes in MYC protein levels. Our preliminary studies indicate that these general DNA binding patterns are also conserved in the case of MYCN, the expression of which is largely confined to cell lines derived from neuroblastomas and a limited number of other cancers (Figure S11, Supporting Information).

Although MYC‐regulated genes tend to oversee mitochondrial and ribosomal structure and function, cell cycle, redox regulation, senescence/aging and DNA damage recognition/repair, those with MYC/MAX binding sites in TES‐proximal regions were somewhat more enriched for functions pertaining to differentiation, proliferation and cytokine/immune responses (Figure 3J,K).^[^ ^1b,d^ ^]^ lncRNA genes were also more likely to show TES‐associated MYC/MAX binding (Figure 4A). The precise reasons for these differences remain to be determined but nonetheless indicate that, like the temporal and spatial binding hierarchies of these sites, the functional preferences are also broadly conserved across cell types and species.

The persistence of MYC at both TSS‐ and TES‐proximal sites in MYCi975‐treated 22Rv1 cells belied the more extensive changes in its total levels as previously determined by immuno‐blotting (Figure 1E).^[^ ^8b^ ^]^ Numerous studies have shown MYC protein half‐life to be 15–30 min and that its levels decline by >90% within 12–24 h of adding various MYC inhibitors.^[^ ^8^, ^10^, ^31^ ^]^ Reconciling these seemingly contradictory findings thus leads to the conclusion that, at any given time, a modest fraction of MYC is highly stabilized by virtue of being chromatin‐associated. This is consistent with the fact that newly synthesized MYC and MAX do not immediately associate since they are synthesized in and initially localize to different nuclear compartments.^[^ ^32^ ^]^ The stability of MYC/MAX at its DNA‐bound sites may also partially reflect the fact that MYC inhibitors are much more effective at preventing MYC/MAX heterodimerization than in reversing it.^[^ ^33^ ^]^ Furthermore, nuclear‐localized DNA‐bound MYC may be relatively under‐phosphorylated at Thr_58_/Ser_62_ (a major determinant of MYC's instability) and further shielded from proteasome‐mediated decay.^[^ ^10^, ^34^ ^]^ The marked and rapid changes in gene expression following MYCi975 treatment, despite the persistence of MYC/MAX binding, may thus reflect the tendency of many MYC targets to be exquisitely sensitive to even small changes in MYC levels (Figures 1E and 3A–F).^[^ ^1d^ ^]^ Cells may therefore contain 2 spatially segregated populations of MYC, the first of which is abundant, inherently unstable, and susceptible to MYC inhibitors prior to its association with MAX. In contrast, the second and less abundant population is stable due to its sequestration by chromatin (possibly in an under‐phosphorylated state), its protection from proteasomal degradation, and the resistance of MYC/MAX heterodimers to MYC inhibitors. Precisely how much DNA‐bound MYC's half‐life is extended, why gene expression is so sensitive to small changes in its levels, and whether it serves non‐canonical functions as a result of this persistence are questions that will be important to explore in future work.

Both TSS‐ and TES‐associated MYC and/or MAX binding and their functional outcomes occur in the context of numerous other TFs and co‐factors that promote transcriptionally conducive changes in the local epigenetic landscape and an open chromatin network (Figures 4E–I and 5; File S2, Supporting Information). This includes a high density of “paused” RNAPII that tends to accumulate around TESs where it plays important roles in transcriptional termination, mRNA cleavage, and polyadenylation.^[^ ^35^ ^]^ The distinct binding pattern around MYC/MAX‐bound sites suggests that it also plays additional roles in gene regulation (Figure 4J). This might reduce the likelihood of mRNA termination and increase the probability of DoG transcription. Similarly, the distinct binding patterns of these various TFs in the vicinity of TESs further suggest that their roles are different from those of either intragenic or distal enhancers (Figure 4I).

Four of the 7 genes whose 3′‐end E box sites were mutated for the studies described above resided in tail‐tail configurations and shared only a single binding peak of MYC/MAX in close proximity of one another's TESs (*Git1‐Trp53i13* and *Tspo‐Ttl12*) (Figure 7A). In the latter case, the shared E box appeared to be necessary for the basal expression for both genes, whereas in the former case, the baseline level of *Git1* was only affected by E box mutation. A variation of this type of regulation by intergenic MYC binding was seen for a larger set of gene pairs that were identified in an unbiased manner in 3 different tissues and cell types (Figure 8). These dyads were all arranged head‐to‐tail and demonstrated MYC binding that was sufficiently close to the TSS of one gene and the TES of its neighbor as to be functionally ambiguous and thus unassignable. In these cases, none of which bound MYC anywhere other than in the intergenic region, we observed both monogenic and digenic regulation that could be either positive or negative. Collectively, these studies suggest that MYC/MAX binding to a single shared E box or other binding site can differentially impact the expression of its adjacent genes in both similar or opposite directions. However, it remains likely that under different circumstances or in different tissues, both qualitative and quantitative variations in these patterns are possible.

Several features distinguish MYC/MAX‐associated TESs from classical enhancers. The first and most notable of these is their predictable and nearly always solitary location relative to TESs in contrast to enhancers whose numbers and positions are highly variable and cannot be ascertained a priori (Figure 1B).^[^ ^7^, ^16^, ^26^, ^36^ ^]^ Second is their association with a prominent landmark, namely the cleavage/poly(A) site. Third is their TF/co‐factor binding patterns that distinguish them from both promoters and enhancers (Figure 4I). Fourth is the fact that, unlike enhancers, which positively regulate transcription, MYC/MAX‐bound TES regions can also be suppressive. Fifth is their direct participation in both basal and stress‐responsive DoG transcription (Figures 1C,4K, and 7C, D). DoG transcripts may possess novel properties that include their refractoriness to nuclear export, their maintenance of euchromatin structure, and their read‐through into and activation of adjacent downstream oncogenes that can impact cancer.^[^ ^15^, ^37^ ^]^

Despite the above differences, MYC/MAX‐associated TESs do appear to share some of the functions of classical enhancers as determined by their interactions with both TSSs and true enhancers in a manner that involves DNA loop‐mediated direct contacts (Figure 6C,E,F). In the limited number of directly examined genes, we identified both positive and negative impacts of TES‐associated MYC/MAX binding on total and DoG transcription. This binding appears to represent a somewhat more flexible and specific way of regulating total and DoG transcription than is afforded by enhancers, particularly since it involves closer contacts between sites of mRNA initiation and termination. The ability of MYC/MAX heterodimers bound at one site to interact with those at another and facilitate looping (Figure 7F) is also in keeping with the long‐known ability of MYC and MAX to hetero‐tetramerize.^[^ ^38^ ^]^ At the same time, it should be stressed that, despite the importance of MYC/MAX in the formation of 5′‐ and 3′‐end contacts, it seems likely that this represents only one of many critical factors that are important for establishing these loops, which are likely to be transient in nature, highly tissue‐specific and subject to many additional physiologic and stress‐related factors and post‐translational modifications (Figures 5, 6, and 7).

The studies reported here have focused on MYC and its role in regulating gene expression via binding to TESs. However, it is clear from our analyses that the chromatin epigenetic landscape is associated with and modified accordingly by many factors other than MYC/MAX (Figures 4I and 6D–I). Precisely how these and the modifications they impart influence gene expression, contribute to or modify MYC/MAX DNA binding, and further enable DNA looping and contact with TSSs and enhancers will require individualized attention.

## 4 Experimental Section

### Computational methods

ChIPseq, DNaseseq, ATACseq, ChIA‐PET, and RNAseq datasets utilized in this study were obtained from the ENCODE or GEO databases. A complete list of all metadata employed in the current study can be found in Table S1 (Supporting Information).

All alignments were performed using GRCh38, mm10, and dm6 as the reference sequences for human, mouse, and *D. melanogaster* genomes, respectively. The “gencode.vM21.primary_assembly.annotation_UCSC_names.gtf” (EMCODE accession: ENCSR884DHJ) and “gencode.v29.primary_assembly.annotation_UCSC_names.gtf” (ENCODE accession: ENCSR884DHJ) annotation files^[^ ^39^ ^]^ were used as the reference annotations for mouse and human, respectively.

For the ENCODE ChIPseq datasets, the IDR (Irreproducible Discovery Rate) thresholded peaks provided by ENCODE were directly used as the processed data. For GEO ChIPseq datasets, fastq files were downloaded from the NCBI SRA (Sequence Read Archive) database. Subsequently, these files were analyzed using the ENCODE Transcription Factor and Histone ChIPseq processing pipeline (ENCODE‐DCC/chip‐seq‐pipeline2‐2.2.0),^[^ ^40^ ^]^ which incorporates the ENCODE integrative analysis approach.^[^ ^8a^ ^]^ ChIPseq data for MYCN was obtained from the ReMap2022 database (https://remap2022.univ‐amu.fr/target_page/MYCN:9606).

ChIP peak annotation was performed using the ChIPpeakAnno package (version 3.6.5) in the R/Bioconductor environment. IDR thresholded peaks, obtained in the “narrowPeak” format, were subjected to further analysis. To prepare IDR thresholded peaks for annotation, they were transformed from the “narrowPeak” format into GrangesList forms. The transformed peaks served as the input data for the subsequent annotation process. The annotatePeakInBatch function used the transformed GrangesList forms of ChIPseq peaks as input and assigned functional annotations to each peak based on its genomic coordinates. In this analysis, the binding regions for all genes of interest were defined as ±2.5 kb (±1 kb for *D. melanogaster*) relative to each transcript's TSS or TES. To annotate the ChIP peaks, the appropriate AnnotationData for the respective organisms was utilized. For human data, the TxDb.Hsapiens.UCSC.hg38.The knownGene(3.15.0) database was used. For mouse data, the TxDb.Mmusculus.UCSC.mm10.knownGene(3.10.0) database was employed and for *D. melanogaster*, the TxDb.Dmelanogaster.UCSC.dm6.ensGene database was utilized.

For ENCODE RNAseq datasets, gene quantification files available from the ENCODE website were directly utilized as the processed data. For GEO RNAseq datasets, raw FASTQ files were downloaded from the NCBI SRA database and analyzed using the nf‐core‐rnaseq‐3.4 pipeline. Most of the RNAseq results in our study were derived from ENCODE datasets, which incorporate spike‐in controls to ensure accurate normalization, correct for per‐cell mRNA differences, and enable robust cross‐sample comparisons. For GEO‐derived RNAseq data lacking such controls, we applied the DESeq2 normalization method instead.^[^ ^41^ ^]^

For gene set over‐representation analyses, gene sets were obtained using the R package msigdbr (version 7.5.1) and subsequently refined to include sets categorized as “C5: Gene Ontology resource (GO)”. The R package ClusterProfiler (version 4.10.0) from Bioconductor^[^ ^42^ ^]^ was employed to conduct over‐representation analysis using the enrichGO function, with a filter applied to retain results featuring adjusted *p*‐values <0.05.

To define transcriptional landscapes of promoters and enhancers, Cis Regulatory Module (CRM) annotations were derived from the ReMap2022 database, which integrates ChIPseq results from multiple sources and biotypes and thereby provides a multi‐cellular multi‐tissue regulatory map.^[^ ^43^ ^]^ ReMap2022 covers 1210 TFs and co‐factors from 737 human cell lines and tissues, 8103 QC ChIPseq datasets and 182 million binding sites. CRM annotations were first filtered so as to include all regions with MYC binding in common (File S2, Supporting Information). Based on mapping to the human genome (version GRCh38), these “MYC CRMs” were assigned to TSSs or TESs as previously noted (Figure 1A,B). For all MYC CRMs sit, CCRE annotation^[^ ^8a^ ^]^ was used to determine whether they met the criteria as enhancers, which were then assigned to regions within genes (intragenic enhancers) or to distal regions (extragenic enhancers) located >2.5 kb upstream of TSSs or >2.5 kb downstream of TESs. The percentage of other transcriptional regulators associated with MYC in these regions was also calculated and then transformed to z‐scores. Each factor's z‐score in each cCRE‐genome region was used as a matrix for heatmaps, which were generated using the ComplexHeatmap R package.

Motif analyses to identify putative bind sites at 5′‐ and 3′‐ends of their target genes were performed in R using memes 1.10.0/ MEME Suite 5.5.0 and universalmotif. Enriched motifs were identified using the runAME function from memes with motif Databases HOCOMOCO Human (v11 FULL) as known motif database and a control set to “shuffle” for the input sequences. The runFimo function was used to identify individual motif occurrences.

For read‐through/DoG analyses, we used ENCODE, rRNA‐depleted strand‐specific RNAseq datasets. Output BAM files were generated by aligning to either the human genome (assembly: GRCh38, genome annotation: V29) or the mouse genome (assembly: mm10, genome annotation: M21). GEO total RNAseq was aligned using the same genome assembly and annotation, with detailed information listed in Table S1 (Supporting Information). Additional analyses utilized DoGFinder software^[^ ^44^ ^]^ were used. Two different gene boundaries were considered, corresponding to stranded and unstranded RNAseq, respectively. Gene boundaries were set so as to be as inclusive as possible based on gene annotation V29 (human) or M21 (mouse), considering strand specificity or lack thereof. We defined the read count in the 500 bp downstream of the TES as read through and the 500 bp upstream of the TES as total gene expression. The Get_DoGs_rpkm function was used for reading counting total and reading through.

Chromatin Interaction Analysis Using Paired‐End Tag Sequencing (ChIA‐PET) data from ENCODE utilized Pol II results.^[^ ^25b^ ^]^ Interaction loop information files in bedpe format were downloaded from ENCODE (see Table S1, Supporting Information for details). The assignment of loop ends was performed by associating them with gene ends, employing the same approach as the binding assignment for MYC/MAX gene ends.

### gRNA Vectors

gRNA sequences were generated to allow targeting of MYC/MAX‐binding E boxes of interest residing in proximity of TESs. The pDG458 vector, which encodes EGFP and can accommodate 2 gRNAs, was obtained from Addgene (Watertown, MA). Plasmid DNAs were purified using Qiagen columns according to the supplier's protocol (Qiagen). gRNAs were directionally cloned into the vector using a single‐step protocol (https://media.addgene.org/data/plasmids/100/100900/100900‐attachment_Yl0i43bWJig3.pdf). Following introduction into chemically competent *E. coli* (Thermo Scientific DH5a competent cells, Thermo‐Fisher, Pittsburgh, PA), plasmid DNAs from isolated bacterial colonies were purified and the identities and orientations of the gRNA‐encoding inserts were confirmed by automated DNA sequencing. All gRNA‐encoding oligonucleotides were chemically synthesized by IDT, Inc. (Coralville, IA) and are shown in Figure S9 and Table S3 (Supporting Information).

### Generation and Characterization of Murine Fibroblasts Lacking TES‐Proximal E Boxes

NIH 3T3 murine fibroblasts were obtained from the ATCC (Manassas, VA) and routinely cultured in Dulbecco's‐modified minimal essential medium supplemented with 10% fetal bovine serum (FBS) (Atlanta Biological, Inc. Flowery Branch, GA) L‐glutamine (200 mM), penicillin (100 units mL^−1^) and streptomycin (100 µg mL^−1^) (all from Sigma‐Aldrich, Inc. St. Louis, MO). Cells were seeded the day before transfection into 6‐well plates so as to be 30–50% confluent at the time of transfection. Transfections with gRNA‐encoding pDG458 vectors (3 µg) were performed using Lipofectamine according to the directions of the supplier (Thermo‐Fisher, Inc. Pittsburgh, PA). Because each vector encoded gRNAs targeting 2 different genes, a single NIH3T3 clone could potentially harbor E‐box mutations in both genes. 2–3 days after transfection, single EGFP+ cells were sorted into 96‐well plates using a FacsAria IIu Cell Sorter (BD Biosciences, San Jose, CA) and expanded into individual clones. Total cellular DNAs from randomly selected clones were then purified using DNeasy kits (Qiagen). PCR reactions (12 mL) using 100 ng of input genomic DNA and Pfu DNA Polymerase (Promega, Inc. Madison, WI) were performed to amplify regions flanking the targeted E boxes using the PCR primers listed in Table S3 (Supporting Information). Aliquots of PCR products from genes with consensus palindromic CACGTG E boxes were digested with 5 units the restriction enzyme PmlI to determine whether the product was fully or partially resistant to digestion and thereby indicating the presence of a homozygous E box mutation. PCR fragments from genes containing non‐consensus E box elements were melted, re‐annealed and digested with one unit of T7 endonuclease 1 according to the protocol of the supplier (New England Biolabs, Inc., Ipswich, MA). This permitted the identification of heteroduplexes between WT and mutant sequences or between two different mutant sequences. Upon confirming that all E boxes had been mutated or deleted, PCR products were subjected to amplicon sequencing to identify all mutations that were present (Azenta Life Sciences, Chelmsford, MA). Because NIH3T3 cells are known to be hypertriploid, more than 2 mutant alleles were often detected (Figure S9, Supporting Information) (https://nih3t3.com).

### Quantification of Total Transcripts, DoG Transcription, and TSS‐TES Contacts

RNA extractions were performed using RNeasy columns (Qiagen) and cDNAs were synthesized by SuperScript IV First‐Strand Synthesis System as previously described.^[^ ^28^ ^]^ Transcripts were quantified by qRT‐PCR using the Syber Green method and the primers listed in Table S4 (Supporting Information).^[^ ^1^, ^45^ ^]^ These were normalized against a control qRT‐PCR reaction for transcripts encoding TBP. DoG transcripts were quantified by first performing strand‐specific reverse transcription for each gene followed by qRT‐PCR as described above. DoG transcript levels were then normalized to those obtained for total transcript levels. Strand‐specific RT primers and those for subsequent qPCR are listed in Table S4 (Supporting Information).

### PCR‐Based Assay for TES‐TSS Interactions

A qPCR‐based assay was employed to quantify interactions between TES and TSS regions as indicated in Figure 7E. Briefly, ≈10^7^ log‐phase WT and E box mutant NIH3T3 cells were cross‐linked with 1% formaldehyde (FA) for 20 min, quenched with 0.2 m glycine and washed with Dulbeccos‐PBS (DPBS). The FA‐crosslinked cells were further cross‐linked with 2 mm EGS [ethylene glycol bis(succinimidyl succinate)] for 45 min, quenched again with 0.2 m glycine, and washed with DPBS. The cross‐linked cells were lysed with 0.1% SDS in the presence of protease inhibitors (Protease Inhibitor Cocktail, MilliporeSigma) at 4 °C for 1 h. Nuclei were further permeabilized with 0.55% SDS at 25 °C for 10 min, followed by incubation at 62 °C for 10 min, and then at 37 °C for 10 min. SDS was quenched with Triton X‐100 at 37 °C for 15 min. The chromatin was then digested to completion with 400 U of DpnII (New England Biolabs, Inc., Ipswich, MA) in 1X DpnII NEBuffer at 37 °C for 24 h. The enzyme was inactivated at 65 °C for 20 min and desalted using a 3 kDa cutoff centrifugal filter (Millipore, Inc. Burlington, MA). The DpnII‐digested chromatin was next ligated in 1X NEB ligase buffer with 4000 U of NEB T4 ligase overnight at 16 °C and then at 4 °C for an additional 48 h. FA cross‐links were then reversed with proteinase K (1 mg mL^−1^) at 65 °C overnight. Following phenol/chloroform/isoamyl alcohol extraction and precipitation with ethanol, the success of the digestion and ligation steps was verified using agarose gel electrophoresis. Ligated DNAs were quantified using a NanoDrop spectrophotometer. SybrGreen‐based PCR reactions were used to amplify the TES‐TSS junctions, with primer sequences and positions shown in Figure S10 and Table S5 (Supporting Information). The amplicon from each PCR reaction was verified by automated sequencing (Azenta Life Sciences, Waltham, MA). Final PCR products were also checked by agarose gel electrophoresis at the end of each assay to ensure that only single bands had been obtained, and could be re‐digested with DpnII to generate products of the predicted size. A region of the *Mlx* gene lacking DpnII site was used for normalization by TaqMan PCR assay (forward primer sequence: 5′‐AGTCCGCTGGCTTGTTT‐3′, reverse primer sequence: 5′‐TTGACCCAAGGGTCCTC‐3′, probe sequence: 5′‐/56‐FAM/CGGTTCGGT/ZEN/AGGTTCACGATGACG/3IABkFQ/‐3′).

### Statistical Analysis

Quantification and statistical analysis were performed using R software v4.3 (R Foundation for Statistical Computing, Vienna, Austria) and GraphPad Prism v9.00 (GraphPad Software Inc., USA). Data are shown as mean ± SEM. When comparing 2 groups, a 2‐tailed unpaired Student's *t*‐test or a Mann‐Whitney *U* test was performed. When comparing >2 groups, one‐way ANOVA or Kruskal–Wallis tests were used. Correlative analyses were performed using a Pearson correlation test. All experiments were performed on at least 3 biological replicates.

## Conflict of Interest

The authors declare no conflict of interest.

## Author Contributions

H.W. compiled and analyzed genomics data and performed ChIP studies; B.M., T.S., J.K., and J.L. generated and characterized cell lines and conducted qRT‐PCR studies; E.V.P. conceived the study and E.V.P. and H.W. devised experiments, analyzed data, and wrote the manuscript. All authors read and approved the final version of the manuscript prior to submission for publication.

## Supporting information



Supporting Information

Supporting Information

Supporting Information

Supporting Information

## Data Availability

The data that support the findings of this study are available from the corresponding author upon reasonable request.

## References

[1] a) D. Levens , Cold Spring Harb Perspect. Med. 2013, 3, a014233; 24186489 10.1101/cshperspect.a014233 PMC3808771

[2] M. Scafuro , L. Capasso , V. Carafa , L. Altucci , A. Nebbioso , Int. J. Mol. Sci. 2021, 22, 3458. 33801599 10.3390/ijms22073458 PMC8037706

[3] a) M. Kalkat , D. Resetca , C. Lourenco , P. K. Chan , Y. Wei , Y. J. Shiah , N. Vitkin , Y. F. Tong , M. Sunnerhagen , S. J. Done , P. C. Boutros , B. Raught , L. Z. Penn , Mol. Cell 2018, 72, 836; 30415952 10.1016/j.molcel.2018.09.031

[4] a) D. Diolaiti , L. McFerrin , P. A. Carroll , R. N. Eisenman , Biochim. Biophys. Acta 2015, 1849, 484; 24857747 10.1016/j.bbagrm.2014.05.016 PMC4241192

[5] a) A. C. Davis , M. Wims , G. D. Spotts , S. R. Hann , A. Bradley , Genes Dev. 1993, 7, 671; 8458579 10.1101/gad.7.4.671

[6] a) A. L. Gartel , K. Shchors , Exp. Cell Res. 2003, 283, 17; 12565816 10.1016/s0014-4827(02)00020-4

[7] a) K. Hamamoto , T. Fukaya , Curr. Opin. Cell Biol. 2022, 74, 62; 35168174 10.1016/j.ceb.2022.01.003

[8] a) Encode Project Consortium , Science 2004, 306, 636; 15499007 10.1126/science.1105136

[9] M. I. Truica , M. C. Burns , H. Han , S. A. Abdulkadir , Cancer Res. 2021, 81, 248. 33087323 10.1158/0008-5472.CAN-20-2959 PMC7855142

[10] A. S. Farrell , R. C. Sears , Cold Spring Harbor Perspect. Med. 2014, 4, a014365. 10.1101/cshperspect.a014365 PMC3935390 24591536

[11] J. Shih , R. Hodge , M. A. Andrade‐Navarro , Genomics Data 2015, 3, 49. 26484147 10.1016/j.gdata.2014.11.010 PMC4536057

[12] A. Sabo , T. R. Kress , M. Pelizzola , S. de Pretis , M. M. Gorski , A. Tesi , M. J. Morelli , P. Bora , M. Doni , A. Verrecchia , C. Tonelli , G. Faga , V. Bianchi , A. Ronchi , D. Low , H. Muller , E. Guccione , S. Campaner , B. Amati , Nature 2014, 511, 488. 25043028 10.1038/nature13537 PMC4110711

[13] T. Habib , H. Park , M. Tsang , I. M. de Alboran , A. Nicks , L. Wilson , P. S. Knoepfler , S. Andrews , D. J. Rawlings , R. N. Eisenman , B. M. Iritani , J. Cell Biol. 2007, 179, 717. 17998397 10.1083/jcb.200704173 PMC2080907

[14] M. Kalkat , J. De Melo , K. A. Hickman , C. Lourenco , C. Redel , D. Resetca , A. Tamachi , W. B. Tu , L. Z. Penn , Genes 2017, 8, 151. 28587062 10.3390/genes8060151 PMC5485515

[15] a) P. Humburg , N. Maugeri , W. Lee , B. Mohr , J. C. Knight , Genome Med. 2016, 8, 87; 27553423 10.1186/s13073-016-0345-5 PMC4995779

[16] a) M. Bacabac , W. Xu , Cancer Metastasis Rev. 2023, 42, 471; 37059907 10.1007/s10555-023-10103-4 PMC10527203

[17] D. Hnisz , D. S. Day , R. A. Young , Cell 2016, 167, 1188. 27863240 10.1016/j.cell.2016.10.024 PMC5125522

[18] X. Tian , S. Zhang , L. Zhou , A. A. Seyhan , L. Hernandez Borrero , Y. Zhang , W. S. El‐Deiry , Front. Pharmacol. 2021, 12, 747837. 34630117 10.3389/fphar.2021.747837 PMC8498116

[19] H. Wang , J. M. Dolezal , S. Kulkarni , J. Lu , J. Mandel , L. E. Jackson , F. Alencastro , A. W. Duncan , E. V. Prochownik , J. Biol. Chem. 2018, 293, 14740. 30087120 10.1074/jbc.RA118.004099 PMC6153302

[20] T. L. Bailey , M. Boden , F. A. Buske , M. Frith , C. E. Grant , L. Clementi , J. Ren , W. W. Li , W. S. Noble , Nucleic Acids Res. 2009, 37, W202. 19458158 10.1093/nar/gkp335 PMC2703892

[21] R. E. Debaugny , J. A. Skok , Curr. Opin. Genet. Dev. 2020, 61, 44. 32334335 10.1016/j.gde.2020.02.021 PMC7893514

[22] M. Allevato , E. Bolotin , M. Grossman , D. Mane‐Padros , F. M. Sladek , E. Martinez , PLoS One 2017, 12, 0180147. 10.1371/journal.pone.0180147 PMC5515408 28719624

[23] S. S. P. Rao , S. C. Huang , B. G. St Hilaire , J. M. Engreitz , E. M. Perez , K. R. Kieffer‐Kwon , A. L. Sanborn , S. E. Johnstone , G. D. Bascom , I. D. Bochkov , X. Huang , M. S. Shamim , J. Shin , D. Turner , Z. Ye , A. D. Omer , J. T. Robinson , T. Schlick , B. E. Bernstein , R. Casellas , E. S. Lander , E. L. Aiden , Cell 2017, 171, 305. 28985562 10.1016/j.cell.2017.09.026 PMC5846482

[24] a) P. Allepuz‐Fuster , M. J. O'Brien , N. Gonzalez‐Polo , B. Pereira , Z. Dhoondia , A. Ansari , O. Calvo , Nucleic Acids Res. 2019, 47, 8975; 31304538 10.1093/nar/gkz597 PMC6753479

[25] a) E. de Wit , W. de Laat , Genes Dev. 2012, 26, 11; 22215806 10.1101/gad.179804.111 PMC3258961

[26] a) O. V. Kyrchanova , O. V. Bylino , P. G. Georgiev , Front. Genet. 2022, 13, 1081088; 36531247 10.3389/fgene.2022.1081088 PMC9751008

[27] T. Curran , R. Bravo , R. Muller , Cancer Surv. 1985, 4, 655. 3939686

[28] S. Kulkarni , J. M. Dolezal , H. Wang , L. Jackson , J. Lu , B. P. Frodey , A. Dosunmu‐Ogunbi , Y. Li , M. Fromherz , A. Kang , L. Santana‐Santos , P. V. Benos , E. V. Prochownik , PLoS One 2017, 12, 0182705. 10.1371/journal.pone.0182705 PMC5562309 28820908

[29] a) P. J. M. Davidson If , Nat. Rev. Mol. Cell Biol. 2021, 22, 445; 33767413 10.1038/s41580-021-00349-7

[30] a) S. A. Lambert , A. Jolma , L. F. Campitelli , P. K. Das , Y. Yin , M. Albu , X. Chen , J. Taipale , T. R. Hughes , M. T. Weirauch , Cell 2018, 172, 650; 29425488 10.1016/j.cell.2018.01.029 PMC12908702

[31] H. Wang , L. Sharma , J. Lu , P. Finch , S. Fletcher , E. V. Prochownik , Oncotarget 2015, 6, 15857. 26036281 10.18632/oncotarget.4327 PMC4599242

[32] X. Y. Yin , M. F. Landay , W. P. Han , E. S. Levitan , S. C. Watkins , R. M. Levenson , D. L. Farkas , E. V. Prochownik , Oncogene 2001, 20, 4650. 11498788 10.1038/sj.onc.1204606

[33] S. Fletcher , E. V. Prochownik , Biochim. Biophys. Acta 2015, 1849, 525. 24657798 10.1016/j.bbagrm.2014.03.005 PMC4169356

[34] a) M. A. Gregory , Y. Qi , S. R. Hann , J. Biol. Chem. 2003, 278, 51606; 14563837 10.1074/jbc.M310722200

[35] a) A. Abuhashem , V. Garg , A. K. Hadjantonakis , Open Biol. 2022, 12, 210220; 34982944 10.1098/rsob.210220 PMC8727152

[36] J. M. Karnuta , P. C. Scacheri , Hum. Mol. Genet. 2018, 27, R219. 29726898 10.1093/hmg/ddy167 PMC6061867

[37] A. R. Grosso , A. P. Leite , S. Carvalho , M. R. Matos , F. B. Martins , A. C. Vitor , J. M. Desterro , M. Carmo‐Fonseca , S. F. de Almeida , eLife 2015, 4, 09214. 10.7554/eLife.09214 PMC4744188 26575290

[38] S. K. Nair , S. K. Burley , Cell 2003, 112, 193. 12553908 10.1016/s0092-8674(02)01284-9

[39] J. Harrow , A. Frankish , J. M. Gonzalez , E. Tapanari , M. Diekhans , F. Kokocinski , B. L. Aken , D. Barrell , A. Zadissa , S. Searle , I. Barnes , A. Bignell , V. Boychenko , T. Hunt , M. Kay , G. Mukherjee , J. Rajan , G. Despacio‐Reyes , G. Saunders , C. Steward , R. Harte , M. Lin , C. Howald , A. Tanzer , T. Derrien , J. Chrast , N. Walters , S. Balasubramanian , B. Pei , M. Tress , et al., Genome Res. 2012, 22, 1760. 22955987 10.1101/gr.135350.111 PMC3431492

[40] B. C. Hitz , J. W. Lee , O. Jolanki , M. S. Kagda , K. Graham , P. Sud , I. Gabdank , J. S. Strattan , C. A. Sloan , T. Dreszer , L. D. Rowe , N. R. Podduturi , V. S. Malladi , E. T. Chan , J. M. Davidson , M. Ho , S. Miyasato , M. Simison , F. Tanaka , Y. Luo , I. Whaling , E. L. Hong , B. T. Lee , R. Sandstrom , E. Rynes , J. Nelson , A. Nishida , A. Ingersoll , M. Buckley , M. Frerker , et al., Res. Sq. 2023, 10.21203/rs.3.rs-3111932/v1.

[41] a) P. A. Ewels , A. Peltzer , S. Fillinger , H. Patel , J. Alneberg , A. Wilm , M. U. Garcia , P. Di Tommaso , S. Nahnsen , Nat. Biotechnol. 2020, 38, 276; 32055031 10.1038/s41587-020-0439-x

[42] T. Wu , E. Hu , S. Xu , M. Chen , P. Guo , Z. Dai , T. Feng , L. Zhou , W. Tang , L. Zhan , X. Fu , S. Liu , X. Bo , G. Yu , Innovation 2021, 2, 100141. 34557778 10.1016/j.xinn.2021.100141 PMC8454663

[43] F. Hammal , P. de Langen , A. Bergon , F. Lopez , B. Ballester , Nucleic Acids Res. 2022, 50, D316. 34751401 10.1093/nar/gkab996 PMC8728178

[44] Y. Wiesel , N. Sabath , R. Shalgi , BMC Genomics 2018, 19, 597. 30089468 10.1186/s12864-018-4983-4 PMC6083495

[45] H. Wang , J. Lu , J. A. Mandel , W. Zhang , M. Schwalbe , J. Gorka , Y. Liu , B. Marburger , J. Wang , S. Ranganathan , E. V. Prochownik , Cell Mol. Gastroenterol. Hepatol. 2021, 12, 199. 33618031 10.1016/j.jcmgh.2021.02.004 PMC8102178

[46] C. Leibiger , N. Kosyakova , H. Mkrtchyan , M. Glei , V. Trifonov , T. Liehr , J. Histochem. Cytochem. 2013, 61, 306. 23321776 10.1369/0022155413476868 PMC3621507

