# Supplementary material for: MYC Binding Near Transcriptional End Sites Regulates Basal Gene Expression, Read‐Through Transcription, and Intragenic Contacts
Source: Adv Sci (Weinh). 2025 May 30;12(31):e14601. doi: 10.1002/advs.202414601 (PMC12376626; doi:10.1002/advs.202414601)
Supplement: Supplementary file 1 — Supporting Information [file ADVS-12-e14601-s002.pdf]

ADVANCED SCIENCE
Open Access

Supporting Information

for *Adv. Sci.*, DOI 10.1002/advs.202414601

MYC Binding Near Transcriptional End Sites Regulates Basal Gene Expression, Read-Through Transcription, and Intragenic Contacts

*Huabo Wang, Bingwei Ma, Taylor Stevens, Jessica Knapp, Jie Lu and Edward V. Prochownik**

Supporting Information

MYC Binding Near Transcriptional End Sites Regulates Basal Gene Expression, Read-Through Transcription and Intragenic Contacts

Huabo Wang, Bingwei Ma, Taylor Stevens, Jessica Knapp, Jie Lu, Edward V. Prochownik

Supplemental Tables ------- Table S1-S5
Supplemental Figures ------- Figure S1-S11
Supplemental File ------- File S1-S3

Table S1. Cell lines and data sets used in the current study

Assay type	Database	File accession ID	Experiment accession	Genome assembly	Biosample term name	Biosample organism	Experiment target	Biosample treatments
ChIP-seq	ENCODE	ENCFF598BZD	ENCSR000DYC	GRCh38	A549	Homo sapiens	MYC-human	
ChIP-seq	ENCODE	ENCFF713RHL	ENCSR000BTJ	GRCh38	A549	Homo sapiens	MAX-human	
ChIP-seq	ENCODE	ENCFF342ASE	ENCSR000ERN	mm10	CH12.LX	Mus musculus	MYC-mouse	
ChIP-seq	ENCODE	ENCFF349LDZ	ENCSR000ERL	mm10	CH12.LX	Mus musculus	MAX-mouse	
ChIP-seq	ENCODE	ENCFF270GMO	ENCSR000EBY	GRCh38	H1	Homo sapiens	MYC-human	
ChIP-seq	ENCODE	ENCFF460LNS	ENCSR000BSJ	GRCh38	H1	Homo sapiens	MAX-human	
ChIP-seq	ENCODE	ENCFF239IMY	ENCSR784BVD	GRCh38	HepG2	Homo sapiens	MYC-human	
ChIP-seq	ENCODE	ENCFF254ZDA	ENCSR168DYA	GRCh38	HepG2	Homo sapiens	MAX-human	
ChIP-seq	ENCODE	ENCFF608CXN	ENCSR000EGJ	GRCh38	K562	Homo sapiens	MYC-human	
ChIP-seq	ENCODE	ENCFF822FKQ	ENCSR000BLP	GRCh38	K562	Homo sapiens	MAX-human	
ChIP-seq	ENCODE	ENCFF152JNC	ENCSR000EUA	mm10	MEL	Mus musculus	MYC-mouse	
ChIP-seq	ENCODE	ENCFF262ITC	ENCSR000ETX	mm10	MEL	Mus musculus	MAX-mouse	
ChIP-seq	ENCODE	ENCFF858ZYN	ENCSR000EHR	GRCh38	NB4	Homo sapiens	MYC-human	
ChIP-seq	ENCODE	ENCFF793GVV	ENCSR000EHS	GRCh38	NB4	Homo sapiens	MAX-human	
ChIP-seq	ENCODE	ENCFF562ZOV	ENCSR000EEZ	GRCh38	endothelial cell of umbilical vein (HUVEC)	Homo sapiens	MAX-human	
ChIP-seq	ENCODE	ENCFF459QFK	ENCSR000DLU	GRCh38	endothelial cell of umbilical vein (HUVEC)	Homo sapiens	MYC-human	
ChIP-seq	ENCODE	ENCFF327QOB	ENCSR999ZCR	dm6	whole organism	Drosophila melanogaster	Myc-dmelanogaster	
ChIP-seq	ENCODE	ENCFF629XNY	ENCSR672KPS	dm6	whole organism	Drosophila melanogaster	Max-dmelanogaster	
ChIP-seq	ENCODE	ENCFF341QUK	ENCSR191VCQ	dm6	whole organism	Drosophila melanogaster	Myc-dmelanogaster	
ChIP-seq	ENCODE	ENCFF409EHG	ENCSR949RDZ	dm6	Kc167	Drosophila melanogaster	Myc-dmelanogaster	
ATAC-seq	ENCODE	ENCFF735UWS	ENCSR220ASC	GRCh38	A549	Homo sapiens		
ATAC-seq	ENCODE	ENCFF333TAT	ENCSR868FGK	GRCh38	K562	Homo sapiens		
ATAC-seq	ENCODE	ENCFF439EIO	ENCSR291GJU	GRCh38	HepG2	Homo sapiens		
DNase-seq	ENCODE	ENCFF291OQF	ENCSR000CNN	mm10	MEL	Mus musculus		
DNase-seq	ENCODE	ENCFF855RCO	ENCSR000CMQ	mm10	CH12.LX	Mus musculus		
DNase-seq	ENCODE	ENCFF983UCL	ENCSR000EMU	GRCh38	H1	Homo sapiens		
DNase-seq	ENCODE	ENCFF128ZVL	ENCSR000ELW	GRCh38	A549	Homo sapiens		
DNase-seq	ENCODE	ENCFF274YGF	ENCSR000EKS	GRCh38	K562	Homo sapiens		
DNase-seq	ENCODE	ENCFF080VVT	ENCSR000EOQ	GRCh38	endothelial cell of umbilical vein (HUVEC)	Homo sapiens		
DNase-seq	ENCODE	ENCFF218WIP	ENCSR000EPL	GRCh38	NB4	Homo sapiens		
DNase-seq	ENCODE	ENCFF897NME	ENCSR149XIL	GRCh38	HepG2	Homo sapiens		
ChIP-seq	ENCODE	ENCFF398DMC	ENCSR000ERI	mm10	CH12.LX	Mus musculus	EP300-mouse	
ChIP-seq	ENCODE	ENCFF185UAQ	ENCSR000ETV	mm10	MEL	Mus musculus	EP300-mouse	
ChIP-seq	ENCODE	ENCFF702XPO	ENCSR000EGE	GRCh38	K562	Homo sapiens	EP300-human	
ChIP-seq	ENCODE	ENCFF678QTH	ENCSR886OEO	GRCh38	A549	Homo sapiens	EP300-human	
ChIP-seq	ENCODE	ENCFF244VKF	ENCSR000BKK	GRCh38	H1	Homo sapiens	EP300-human	
ChIP-seq	ENCODE	ENCFF827LSX	ENCSR271XMW	GRCh38	HepG2	Homo sapiens	EP300-human	
ChIP-seq	ENCODE	ENCFF125SFM	ENCSR000CGL	mm10	CH12.LX	Mus musculus	H3K9ac-mouse	
ChIP-seq	ENCODE	ENCFF392DCV	ENCSR000ALD	GRCh38	endothelial cell of umbilical vein	Homo sapiens	H3K9ac-human	
ChIP-seq	ENCODE	ENCFF568IPQ	ENCSR000ALD	GRCh38	endothelial cell of umbilical vein	Homo sapiens	H3K9ac-human	
ChIP-seq	ENCODE	ENCFF077LGZ	ENCSR000ALB	GRCh38	endothelial cell of umbilical vein	Homo sapiens	H3K27ac-human	
ChIP-seq	ENCODE	ENCFF317QGQ	ENCSR000ANP	GRCh38	H1	Homo sapiens	H3K27ac-human	
ChIP-seq	ENCODE	ENCFF148UQI	ENCSR000EVZ	GRCh38	K562	Homo sapiens	H3K9ac-human	
ChIP-seq	ENCODE	ENCFF044QMC	ENCSR000AMD	GRCh38	HepG2	Homo sapiens	H3K9ac-human	
ChIP-seq	ENCODE	ENCFF392KDI	ENCSR000AMO	GRCh38	HepG2	Homo sapiens	H3K27ac-human	
ChIP-seq	ENCODE	ENCFF395EHX	ENCSR000CGJ	mm10	CH12.LX	Mus musculus	H3K27ac-mouse	
ChIP-seq	ENCODE	ENCFF544LXB	ENCSR000AKP	GRCh38	K562	Homo sapiens	H3K27ac-human	
ChIP-seq	ENCODE	ENCFF747IZX	ENCSR778NQS	GRCh38	A549	Homo sapiens	H3K27ac-human	
ChIP-seq	ENCODE	ENCFF900QYB	ENCSR000CEU	mm10	MEL	Mus musculus	H3K9ac-mouse	
ChIP-seq	ENCODE	ENCFF972YIT	ENCSR000CEV	mm10	MEL	Mus musculus	H3K27ac-mouse	
ChIP-seq	ENCODE	ENCFF679LHF	ENCSR441UHO	GRCh38	H1	Homo sapiens	H3K9ac-human	
ChIP-seq	ENCODE	ENCFF354VWZ	ENCSR000EEM	GRCh38	HepG2	Homo sapiens	POLR2A-human	
ChIP-seq	ENCODE	ENCFF322DAE	ENCSR000BHN	GRCh38	H1	Homo sapiens	POLR2A-human	
ChIP-seq	ENCODE	ENCFF004KQA	ENCSR000EUC	mm10	MEL	Mus musculus	POLR2A-mouse	
ChIP-seq	ENCODE	ENCFF269DMJ	ENCSR000ERQ	mm10	CH12.LX	Mus musculus	POLR2A-mouse	
ChIP-seq	ENCODE	ENCFF730QNU	ENCSR000FAL	GRCh38	NB4	Homo sapiens	POLR2A-human	
ChIP-seq	ENCODE	ENCFF229KAY	ENCSR000BQB	GRCh38	endothelial cell of umbilical vein	Homo sapiens	POLR2A-human	
ChIP-seq	ENCODE	ENCFF681MRC	ENCSR000DMZ	GRCh38	A549	Homo sapiens	POLR2A-human	
ChIP-seq	ENCODE	ENCFF355MNE	ENCSR388QZF	GRCh38	K562	Homo sapiens	POLR2A-human	
ChIA-PET	ENCODE	ENCFF364UNM	ENCSR857MYZ	GRCh38	HepG2	Homo sapiens	POLR2A-human	
ChIA-PET	ENCODE	ENCFF421KYP	ENCSR138NSW	GRCh38	A549	Homo sapiens	POLR2A-human	

ChIA-PET	ENCODE	ENCFF753NSM	ENCSR782EKZ	GRCh38	H1	Homo sapiens	POLR2A-human	
ChIA-PET	ENCODE	ENCFF308LJF	ENCSR080OMN	GRCh38	endothelial cell of umbilical vein	Homo sapiens	POLR2A-human	
ChIA-PET	ENCODE	ENCFF511QFN	ENCSR880DSH	GRCh38	K562	Homo sapiens	POLR2A-human	
ChIP-seq	GEO	SRR8555222	GSM3596839	mm10	primary naive mouse B-cells	Mus musculus	none(input control)	LPS (50 ug/ml) 0h

Table S1. Cell lines and data sets used in the current study(cont'd)

Assay type	Database	File accession ID	Experiment accession	Genome assembly	Biosample term name	Biosample organism	Experiment target	Biosample treatments
ChIP-seq	GEO	SRR8555223	GSM3596840	mm10	primary naive mouse B-cells	Mus musculus	MYC-mouse	LPS (50 ug/ml) 0h
ChIP-seq	GEO	SRR8555224	GSM3596841	mm10	primary naive mouse B-cells	Mus musculus	MYC-mouse	LPS (50 ug/ml) 2h
ChIP-seq	GEO	SRR8555225	GSM3596842	mm10	primary naive mouse B-cells	Mus musculus	MYC-mouse	LPS (50 ug/ml) 4h
ChIP-seq	GEO	SRR8555226	GSM3596843	mm10	primary naive mouse B-cells	Mus musculus	MYC-mouse	LPS (50 ug/ml) 8h
ChIP-seq	GEO	SRR5493408	GSM2595123	mm10	3T9	Mus musculus	none(input control)	MYC-ER activation by 4-hydroxytamoxifen (400nM)
ChIP-seq	GEO	SRR5493409	GSM2595124	mm10	3T9	Mus musculus	MYC-mouse	0 min MYC-ER activation by 4-hydroxytamoxifen (400nM)
ChIP-seq	GEO	SRR5493410	GSM2595125	mm10	3T9	Mus musculus	MYC-mouse	10 min MYC-ER activation by 4-hydroxytamoxifen (400nM)
ChIP-seq	GEO	SRR5493411	GSM2595126	mm10	3T9	Mus musculus	MYC-mouse	20 min MYC-ER activation by 4-hydroxytamoxifen (400nM)
ChIP-seq	GEO	SRR5493412	GSM2595127	mm10	3T9	Mus musculus	MYC-mouse	30 min MYC-ER activation by 4-hydroxytamoxifen (400nM)
ChIP-seq	GEO	SRR5493413	GSM2595128	mm10	3T9	Mus musculus	MYC-mouse	2 h MYC-ER activation by 4-hydroxytamoxifen (400nM)
ChIP-seq	GEO	SRR5493414	GSM2595129	mm10	3T9	Mus musculus	MYC-mouse	4 h MYC-ER activation by 4-hydroxytamoxifen (400nM)
ChIP-seq	GEO	SRR3020030	GSM1973344	mm10	normal liver	Mus musculus	MYC-mouse	
ChIP-seq	GEO	SRR3020031	GSM1973345	mm10	normal liver	Mus musculus	MYC-mouse	
ChIP-seq	GEO	SRR3020032	GSM1973346	mm10	normal liver	Mus musculus	MYC-mouse	
ChIP-seq	GEO	SRR3020033	GSM1973347	mm10	normal liver	Mus musculus	MYC-mouse	
ChIP-seq	GEO	SRR3020034	GSM1973348	mm10	tet-Myc liver tumor	Mus musculus	MYC-mouse	
ChIP-seq	GEO	SRR3020035	GSM1973349	mm10	tet-Myc liver tumor	Mus musculus	MYC-mouse	
ChIP-seq	GEO	SRR3020036	GSM1973350	mm10	tet-Myc liver tumor	Mus musculus	MYC-mouse	
ChIP-seq	GEO	SRR3020037	GSM1973351	mm10	tet-Myc liver tumor	Mus musculus	MYC-mouse	
ChIP-seq	GEO	SRR3020038	GSM1973352	mm10	tet-Myc liver tumor	Mus musculus	MYC-mouse	
ChIP-seq	GEO	SRR3020039	GSM1973353	mm10	tet-Myc liver tumor	Mus musculus	MYC-mouse	
ChIP-seq	GEO	SRR3020040	GSM1973354	mm10	tet-Myc liver tumor	Mus musculus	MYC-mouse	
ChIP-seq	GEO	SRR3020041	GSM1973355	mm10	tet-Myc liver tumor	Mus musculus	MYC-mouse	
ChIP-seq	GEO	SRR3020042	GSM1973356	mm10	tet-Myc liver tumor	Mus musculus	MYC-mouse	
ChIP-seq	GEO	SRR3020076	GSM1973390	mm10	normal liver	Mus musculus	none(input control)	
ChIP-seq	GEO	SRR14907854	GSM5399514	GRCh38	22Rv1	Homo sapiens	MYC-human	1hr MYCi - 10 μM
ChIP-seq	GEO	SRR14907855	GSM5399515	GRCh38	22Rv1	Homo sapiens	MYC-human	1hr MYCi - 10 μM
ChIP-seq	GEO	SRR14907856	GSM5399516	GRCh38	22Rv1	Homo sapiens	MYC-human	1hr MYCi - 10 μM
ChIP-seq	GEO	SRR14907991	GSM5399513	GRCh38	22Rv1	Homo sapiens	MYC-human	1hr MYCi - 10 μM
ChIP-seq	GEO	SRR14907857	GSM5399517	GRCh38	22Rv1	Homo sapiens	MAX-human	1hr MYCi - 10 μM
ChIP-seq	GEO	SRR14907858	GSM5399518	GRCh38	22Rv1	Homo sapiens	MAX-human	1hr MYCi - 10 μM
ChIP-seq	GEO	SRR14907859	GSM5399519	GRCh38	22Rv1	Homo sapiens	MAX-human	1hr MYCi - 10 μM
ChIP-seq	GEO	SRR14907860	GSM5399520	GRCh38	22Rv1	Homo sapiens	MAX-human	1hr MYCi - 10 μM
ChIP-seq	GEO	SRR14907863	GSM5399523	GRCh38	22Rv1	Homo sapiens	none(input control)	1hr MYCi - 10 μM
ChIP-seq	GEO	SRR14907864	GSM5399524	GRCh38	22Rv1	Homo sapiens	none(input control)	1hr MYCi - 10 μM
ChIP-seq	GEO	SRR14907865	GSM5399525	GRCh38	22Rv1	Homo sapiens	none(input control)	1hr MYCi - 10 μM
ChIP-seq	GEO	SRR14907866	GSM5399526	GRCh38	22Rv1	Homo sapiens	none(input control)	1hr MYCi - 10 μM
ChIP-seq	GEO	SRR14907896	GSM5399555	GRCh38	22Rv1	Homo sapiens	MYC-human	24hr MYCi - 10 μM
ChIP-seq	GEO	SRR14907897	GSM5399556	GRCh38	22Rv1	Homo sapiens	MYC-human	24hr MYCi - 10 μM
ChIP-seq	GEO	SRR14907898	GSM5399557	GRCh38	22Rv1	Homo sapiens	MYC-human	24hr MYCi - 10 μM
ChIP-seq	GEO	SRR14907899	GSM5399558	GRCh38	22Rv1	Homo sapiens	MYC-human	24hr MYCi - 10 μM
ChIP-seq	GEO	SRR14907900	GSM5399559	GRCh38	22Rv1	Homo sapiens	MAX-human	24hr MYCi - 10 μM
ChIP-seq	GEO	SRR14907901	GSM5399560	GRCh38	22Rv1	Homo sapiens	MAX-human	24hr MYCi - 10 μM
ChIP-seq	GEO	SRR14907902	GSM5399561	GRCh38	22Rv1	Homo sapiens	MAX-human	24hr MYCi - 10 μM
ChIP-seq	GEO	SRR14907903	GSM5399562	GRCh38	22Rv1	Homo sapiens	MAX-human	24hr MYCi - 10 μM
ChIP-seq	GEO	SRR14907908	GSM5399567	GRCh38	22Rv1	Homo sapiens	none(input control)	24hr MYCi - 10 μM
ChIP-seq	GEO	SRR14907909	GSM5399568	GRCh38	22Rv1	Homo sapiens	none(input control)	24hr MYCi - 10 μM
ChIP-seq	GEO	SRR14907910	GSM5399569	GRCh38	22Rv1	Homo sapiens	none(input control)	24hr MYCi - 10 μM
ChIP-seq	GEO	SRR14907911	GSM5399570	GRCh38	22Rv1	Homo sapiens	none(input control)	24hr MYCi - 10 μM
ChIP-seq	GEO	SRR14907912	GSM5399571	GRCh38	22Rv1	Homo sapiens	MYC-human	48hr MYCi - 10 μM
ChIP-seq	GEO	SRR14907913	GSM5399572	GRCh38	22Rv1	Homo sapiens	MYC-human	48hr MYCi - 10 μM
ChIP-seq	GEO	SRR14907914	GSM5399573	GRCh38	22Rv1	Homo sapiens	MYC-human	48hr MYCi - 10 μM
ChIP-seq	GEO	SRR14907915	GSM5399574	GRCh38	22Rv1	Homo sapiens	MYC-human	48hr MYCi - 10 μM
ChIP-seq	GEO	SRR14907916	GSM5399575	GRCh38	22Rv1	Homo sapiens	MAX-human	48hr MYCi - 10 μM
ChIP-seq	GEO	SRR14907917	GSM5399576	GRCh38	22Rv1	Homo sapiens	MAX-human	48hr MYCi - 10 μM
ChIP-seq	GEO	SRR14907918	GSM5399577	GRCh38	22Rv1	Homo sapiens	MAX-human	48hr MYCi - 10 μM
ChIP-seq	GEO	SRR14907919	GSM5399578	GRCh38	22Rv1	Homo sapiens	MAX-human	48hr MYCi - 10 μM
ChIP-seq	GEO	SRR14907922	GSM5399581	GRCh38	22Rv1	Homo sapiens	none(input control)	48hr MYCi - 10 μM
ChIP-seq	GEO	SRR14907923	GSM5399582	GRCh38	22Rv1	Homo sapiens	none(input control)	48hr MYCi - 10 μM
ChIP-seq	GEO	SRR14907924	GSM5399583	GRCh38	22Rv1	Homo sapiens	none(input control)	48hr MYCi - 10 μM
ChIP-seq	GEO	SRR14907925	GSM5399584	GRCh38	22Rv1	Homo sapiens	none(input control)	48hr MYCi - 10 μM
ChIP-seq	GEO	SRR14907867	GSM5399527	GRCh38	22Rv1	Homo sapiens	MYC-human	4hr MYCi - 10 μM
ChIP-seq	GEO	SRR14907868	GSM5399528	GRCh38	22Rv1	Homo sapiens	MYC-human	4hr MYCi - 10 μM

Table S1. Cell lines and data sets used in the current study(cont'd..)

Assay type	Database	File accession ID	Experiment accession	Genome assembly	Biosample term name	Biosample organism	Experiment target	Biosample treatments
ChIP-seq	GEO	SRR14907869	GSM5399529	GRCh38	22Rv1	Homo sapiens	MYC-human	4hr MYCi - 10 µM
ChIP-seq	GEO	SRR14907870	GSM5399530	GRCh38	22Rv1	Homo sapiens	MYC-human	4hr MYCi - 10 µM
ChIP-seq	GEO	SRR14907871	GSM5399531	GRCh38	22Rv1	Homo sapiens	MAX-human	4hr MYCi - 10 µM
ChIP-seq	GEO	SRR14907872	GSM5399532	GRCh38	22Rv1	Homo sapiens	MAX-human	4hr MYCi - 10 µM
ChIP-seq	GEO	SRR14907873	GSM5399533	GRCh38	22Rv1	Homo sapiens	MAX-human	4hr MYCi - 10 µM
ChIP-seq	GEO	SRR14907874	GSM5399534	GRCh38	22Rv1	Homo sapiens	MAX-human	4hr MYCi - 10 µM
ChIP-seq	GEO	SRR14907877	GSM5399537	GRCh38	22Rv1	Homo sapiens	none(input control)	4hr MYCi - 10 µM
ChIP-seq	GEO	SRR14907878	GSM5399538	GRCh38	22Rv1	Homo sapiens	none(input control)	4hr MYCi - 10 µM
ChIP-seq	GEO	SRR14907879	GSM5399539	GRCh38	22Rv1	Homo sapiens	none(input control)	4hr MYCi - 10 µM
ChIP-seq	GEO	SRR14907881	GSM5399540	GRCh38	22Rv1	Homo sapiens	none(input control)	4hr MYCi - 10 µM
ChIP-seq	GEO	SRR14907882	GSM5399541	GRCh38	22Rv1	Homo sapiens	MYC-human	8hr MYCi - 10 µM
ChIP-seq	GEO	SRR14907883	GSM5399542	GRCh38	22Rv1	Homo sapiens	MYC-human	8hr MYCi - 10 µM
ChIP-seq	GEO	SRR14907884	GSM5399543	GRCh38	22Rv1	Homo sapiens	MYC-human	8hr MYCi - 10 µM
ChIP-seq	GEO	SRR14907885	GSM5399544	GRCh38	22Rv1	Homo sapiens	MYC-human	8hr MYCi - 10 µM
ChIP-seq	GEO	SRR14907886	GSM5399545	GRCh38	22Rv1	Homo sapiens	MAX-human	8hr MYCi - 10 µM
ChIP-seq	GEO	SRR14907887	GSM5399546	GRCh38	22Rv1	Homo sapiens	MAX-human	8hr MYCi - 10 µM
ChIP-seq	GEO	SRR14907888	GSM5399547	GRCh38	22Rv1	Homo sapiens	MAX-human	8hr MYCi - 10 µM
ChIP-seq	GEO	SRR14907889	GSM5399548	GRCh38	22Rv1	Homo sapiens	MAX-human	8hr MYCi - 10 µM
ChIP-seq	GEO	SRR14907892	GSM5399551	GRCh38	22Rv1	Homo sapiens	none(input control)	8hr MYCi - 10 µM
ChIP-seq	GEO	SRR14907893	GSM5399552	GRCh38	22Rv1	Homo sapiens	none(input control)	8hr MYCi - 10 µM
ChIP-seq	GEO	SRR14907894	GSM5399553	GRCh38	22Rv1	Homo sapiens	none(input control)	8hr MYCi - 10 µM
ChIP-seq	GEO	SRR14907895	GSM5399554	GRCh38	22Rv1	Homo sapiens	none(input control)	8hr MYCi - 10 µM
ChIP-seq	GEO	SRR14907974	GSM5399497	GRCh38	22Rv1	Homo sapiens	MYC-human	DMSO (0.2%)
ChIP-seq	GEO	SRR14907975	GSM5399498	GRCh38	22Rv1	Homo sapiens	MYC-human	DMSO (0.2%)
ChIP-seq	GEO	SRR14907976	GSM5399499	GRCh38	22Rv1	Homo sapiens	MYC-human	DMSO (0.2%)
ChIP-seq	GEO	SRR14907977	GSM5399500	GRCh38	22Rv1	Homo sapiens	MYC-human	DMSO (0.2%)
ChIP-seq	GEO	SRR14907978	GSM5399501	GRCh38	22Rv1	Homo sapiens	MAX-human	DMSO (0.2%)
ChIP-seq	GEO	SRR14907979	GSM5399502	GRCh38	22Rv1	Homo sapiens	MAX-human	DMSO (0.2%)
ChIP-seq	GEO	SRR14907980	GSM5399503	GRCh38	22Rv1	Homo sapiens	MAX-human	DMSO (0.2%)
ChIP-seq	GEO	SRR14907981	GSM5399504	GRCh38	22Rv1	Homo sapiens	MAX-human	DMSO (0.2%)
ChIP-seq	GEO	SRR14907986	GSM5399509	GRCh38	22Rv1	Homo sapiens	none(input control)	DMSO (0.2%)
ChIP-seq	GEO	SRR14907988	GSM5399510	GRCh38	22Rv1	Homo sapiens	none(input control)	DMSO (0.2%)
ChIP-seq	GEO	SRR14907989	GSM5399511	GRCh38	22Rv1	Homo sapiens	none(input control)	DMSO (0.2%)
ChIP-seq	GEO	SRR14907990	GSM5399512	GRCh38	22Rv1	Homo sapiens	none(input control)	DMSO (0.2%)
polyA plus RNA-seq	ENCODE	ENCFF472HFI	ENCSR000AEP	GRCh38	K562	Homo sapiens		
polyA plus RNA-seq	ENCODE	ENCFF628SMT	ENCSR000AEP	GRCh38	K562	Homo sapiens		
polyA plus RNA-seq	ENCODE	ENCFF486DGZ	ENCSR000AEQ	GRCh38	K562	Homo sapiens		
polyA plus RNA-seq	ENCODE	ENCFF088LCK	ENCSR000AEQ	GRCh38	K562	Homo sapiens		
polyA plus RNA-seq	ENCODE	ENCFF298KDC	ENCSR000CID	mm10	MEL	Mus musculus		
polyA plus RNA-seq	ENCODE	ENCFF952RYM	ENCSR000CID	mm10	MEL	Mus musculus		
polyA plus RNA-seq	ENCODE	ENCFF806WJZ	ENCSR937WIG	GRCh38	A549	Homo sapiens		
polyA plus RNA-seq	ENCODE	ENCFF203NNS	ENCSR937WIG	GRCh38	A549	Homo sapiens		
polyA plus RNA-seq	ENCODE	ENCFF715WBR	ENCSR937WIG	GRCh38	A549	Homo sapiens		
polyA plus RNA-seq	ENCODE	ENCFF842EIL	ENCSR000AEO	GRCh38	K562	Homo sapiens		
polyA plus RNA-seq	ENCODE	ENCFF322EYC	ENCSR000AEO	GRCh38	K562	Homo sapiens		
polyA plus RNA-seq	ENCODE	ENCFF831QQF	ENCSR000CPE	GRCh38	HepG2	Homo sapiens		
polyA plus RNA-seq	ENCODE	ENCFF168QKW	ENCSR000CPE	GRCh38	HepG2	Homo sapiens		
polyA plus RNA-seq	ENCODE	ENCFF355TDA	ENCSR985KAT	GRCh38	HepG2	Homo sapiens		
polyA plus RNA-seq	ENCODE	ENCFF073RKC	ENCSR985KAT	GRCh38	HepG2	Homo sapiens		
polyA plus RNA-seq	ENCODE	ENCFF179CNW	ENCSR000EYO	GRCh38	K562	Homo sapiens		
polyA plus RNA-seq	ENCODE	ENCFF679BCM	ENCSR000EYO	GRCh38	K562	Homo sapiens		
polyA plus RNA-seq	ENCODE	ENCFF068NRZ	ENCSR000CPH	GRCh38	K562	Homo sapiens		
polyA plus RNA-seq	ENCODE	ENCFF928YLB	ENCSR000CPH	GRCh38	K562	Homo sapiens		
polyA plus RNA-seq	ENCODE	ENCFF321HCT	ENCSR000COU	GRCh38	H1	Homo sapiens		
polyA plus RNA-seq	ENCODE	ENCFF736CCO	ENCSR000COU	GRCh38	H1	Homo sapiens		
polyA plus RNA-seq	ENCODE	ENCFF443ZUL	ENCSR000CHM	mm10	MEL	Mus musculus		
polyA plus RNA-seq	ENCODE	ENCFF838AMX	ENCSR000CHM	mm10	MEL	Mus musculus		
polyA plus RNA-seq	ENCODE	ENCFF742CVV	ENCSR000AEM	GRCh38	K562	Homo sapiens		
polyA plus RNA-seq	ENCODE	ENCFF222UVT	ENCSR000AEM	GRCh38	K562	Homo sapiens		
polyA plus RNA-seq	ENCODE	ENCFF006IHP	ENCSR000BZU	GRCh38	H1	Homo sapiens		
polyA plus RNA-seq	ENCODE	ENCFF779OCC	ENCSR000COZ	GRCh38	endothelial cell of umbilical vein (HUVEC)	Homo sapiens		
polyA plus RNA-seq	ENCODE	ENCFF770MAV	ENCSR000COZ	GRCh38	endothelial cell of umbilical vein (HUVEC)	Homo sapiens		
polyA plus RNA-seq	ENCODE	ENCFF855AKQ	ENCSR000CON	GRCh38	A549	Homo sapiens		
polyA plus RNA-seq	ENCODE	ENCFF244DNJ	ENCSR000CON	GRCh38	A549	Homo sapiens		
polyA plus RNA-seq	ENCODE	ENCFF082MCE	ENCSR000CHR	mm10	CH12.LX	Mus musculus		
polyA plus RNA-seq	ENCODE	ENCFF795QMY	ENCSR000CHR	mm10	CH12.LX	Mus musculus		
polyA plus RNA-seq	ENCODE	ENCFF881WXC	ENCSR000EYS	GRCh38	endothelial cell of umbilical vein	Homo sapiens		
polyA plus RNA-seq	ENCODE	ENCFF237XJC	ENCSR000EYS	GRCh38	endothelial cell of umbilical vein	Homo sapiens		
polyA plus RNA-seq	ENCODE	ENCFF233CZT	ENCSR632DQP	GRCh38	A549	Homo sapiens		
polyA plus RNA-seq	ENCODE	ENCFF285BTU	ENCSR632DQP	GRCh38	A549	Homo sapiens		
polyA plus RNA-seq	ENCODE	ENCFF904PCS	ENCSR632DQP	GRCh38	A549	Homo sapiens		
polyA plus RNA-seq	ENCODE	ENCFF360GXF	ENCSR632DQP	GRCh38	A549	Homo sapiens		
polyA plus RNA-seq	ENCODE	ENCFF190XYJ	ENCSR962TBJ	GRCh38	H1	Homo sapiens		
polyA plus RNA-seq	ENCODE	ENCFF296UGP	ENCSR000EYP	GRCh38	H1	Homo sapiens		
polyA plus RNA-seq	ENCODE	ENCFF247KYR	ENCSR000EYP	GRCh38	H1	Homo sapiens		

Table S1. Cell lines and data sets used in the current study(cont'd)

Assay type	Database	File accession ID	Experiment accession	Genome assembly	Biosample term name	Biosample organism	Experiment target	Biosample treatments
polyA plus RNA-seq	ENCODE	ENCFF131NEX	ENCSR000EYP	GRCh38	H1	Homo sapiens		
polyA plus RNA-seq	ENCODE	ENCFF562ECY	ENCSR000EYP	GRCh38	H1	Homo sapiens		
polyA plus RNA-seq	ENCODE	ENCFF121UFS	ENCSR637VLS	GRCh38	K562	Homo sapiens		
polyA plus RNA-seq	ENCODE	ENCFF938DAF	ENCSR637VLS	GRCh38	K562	Homo sapiens		
polyA plus RNA-seq	ENCODE	ENCFF432RPO	ENCSR043RSE	GRCh38	H1	Homo sapiens		
polyA plus RNA-seq	ENCODE	ENCFF695GMA	ENCSR000EYR	GRCh38	HepG2	Homo sapiens		
polyA plus RNA-seq	ENCODE	ENCFF292KIL	ENCSR000EYR	GRCh38	HepG2	Homo sapiens		
polyA plus RNA-seq	ENCODE	ENCFF806WAH	ENCSR643QIZ	GRCh38	H1	Homo sapiens		
polyA plus RNA-seq	ENCODE	ENCFF582HOU	ENCSR643QIZ	GRCh38	H1	Homo sapiens		
polyA plus RNA-seq	ENCODE	ENCFF243GKW	ENCSR670WQY	GRCh38	H1	Homo sapiens		
polyA plus RNA-seq	ENCODE	ENCFF119KXQ	ENCSR561FEE	GRCh38	HepG2	Homo sapiens		
polyA plus RNA-seq	ENCODE	ENCFF308OYV	ENCSR561FEE	GRCh38	HepG2	Homo sapiens		
polyA plus RNA-seq	ENCODE	ENCFF416LVG	ENCSR545DKY	GRCh38	K562	Homo sapiens		
polyA plus RNA-seq	ENCODE	ENCFF556ISR	ENCSR545DKY	GRCh38	K562	Homo sapiens		
total RNA-seq	ENCODE	ENCFF263YGK	ENCSR000AJV	mm10	CH12.LX	Mus musculus		
total RNA-seq	ENCODE	ENCFF525WDQ	ENCSR000AJV	mm10	CH12.LX	Mus musculus		
total RNA-seq	ENCODE	ENCFF465NLB	ENCSR000AJV	mm10	CH12.LX	Mus musculus		
total RNA-seq	ENCODE	ENCFF445CEF	ENCSR000AJV	mm10	CH12.LX	Mus musculus		
total RNA-seq	ENCODE	ENCFF160NVZ	ENCSR414IGI	GRCh38	A549	Homo sapiens		
total RNA-seq	ENCODE	ENCFF017ELJ	ENCSR414IGI	GRCh38	A549	Homo sapiens		
total RNA-seq	ENCODE	ENCFF292XOH	ENCSR414IGI	GRCh38	A549	Homo sapiens		
total RNA-seq	ENCODE	ENCFF823SJE	ENCSR414IGI	GRCh38	A549	Homo sapiens		
total RNA-seq	ENCODE	ENCFF537IEO	ENCSR414IGI	GRCh38	A549	Homo sapiens		
total RNA-seq	ENCODE	ENCFF507RJZ	ENCSR000CWD	mm10	CH12.LX	Mus musculus		
total RNA-seq	ENCODE	ENCFF469ZCH	ENCSR000CWD	mm10	CH12.LX	Mus musculus		
total RNA-seq	ENCODE	ENCFF203XTH	ENCSR000CWD	mm10	CH12.LX	Mus musculus		
total RNA-seq	ENCODE	ENCFF796PCV	ENCSR000CWD	mm10	CH12.LX	Mus musculus		
total RNA-seq	ENCODE	ENCFF472HPD	ENCSR000CWE	mm10	MEL	Mus musculus		
total RNA-seq	ENCODE	ENCFF933SWB	ENCSR000CWE	mm10	MEL	Mus musculus		
total RNA-seq	ENCODE	ENCFF475KFH	ENCSR000CWE	mm10	MEL	Mus musculus		
total RNA-seq	ENCODE	ENCFF082TDF	ENCSR000CWE	mm10	MEL	Mus musculus		
total RNA-seq	ENCODE	ENCFF067CVP	ENCSR181ZGR	GRCh38	HepG2	Homo sapiens		
total RNA-seq	ENCODE	ENCFF343TKA	ENCSR181ZGR	GRCh38	HepG2	Homo sapiens		
total RNA-seq	ENCODE	ENCFF286SDY	ENCSR181ZGR	GRCh38	HepG2	Homo sapiens		
total RNA-seq	ENCODE	ENCFF140GVE	ENCSR181ZGR	GRCh38	HepG2	Homo sapiens		
total RNA-seq	ENCODE	ENCFF379NOY	ENCSR895ZTB	GRCh38	H1	Homo sapiens		
total RNA-seq	ENCODE	ENCFF675NTU	ENCSR895ZTB	GRCh38	H1	Homo sapiens		
total RNA-seq	ENCODE	ENCFF948VKA	ENCSR993JMV	GRCh38	endothelial cell of umbilical vein	Homo sapiens		
total RNA-seq	ENCODE	ENCFF140CNM	ENCSR993JMV	GRCh38	endothelial cell of umbilical vein	Homo sapiens		
total RNA-seq	ENCODE	ENCFF932MJL	ENCSR792OIJ	GRCh38	K562	Homo sapiens		
total RNA-seq	ENCODE	ENCFF170FKF	ENCSR792OIJ	GRCh38	K562	Homo sapiens		
RNA-seq	GEO	SRR3020294	GSM1973514	mm10	normal liver	Mus musculus		
RNA-seq	GEO	SRR3020296	GSM1973515	mm10	normal liver	Mus musculus		
RNA-seq	GEO	SRR3020297	GSM1973516	mm10	normal liver	Mus musculus		
RNA-seq	GEO	SRR3020299	GSM1973517	mm10	normal liver	Mus musculus		
RNA-seq	GEO	SRR3020300	GSM1973518	mm10	normal liver	Mus musculus		
RNA-seq	GEO	SRR3020302	GSM1973519	mm10	normal liver	Mus musculus		
RNA-seq	GEO	SRR3020304	GSM1973520	mm10	normal liver	Mus musculus		
RNA-seq	GEO	SRR3020305	GSM1973521	mm10	normal liver	Mus musculus		
RNA-seq	GEO	SRR3020306	GSM1973522	mm10	normal liver	Mus musculus		
RNA-seq	GEO	SRR3020307	GSM1973523	mm10	normal liver	Mus musculus		
RNA-seq	GEO	SRR3020308	GSM1973524	mm10	normal liver	Mus musculus		
RNA-seq	GEO	SRR3020309	GSM1973525	mm10	tet-Myc liver tumor	Mus musculus		
RNA-seq	GEO	SRR3020310	GSM1973526	mm10	tet-Myc liver tumor	Mus musculus		
RNA-seq	GEO	SRR3020311	GSM1973527	mm10	tet-Myc liver tumor	Mus musculus		
RNA-seq	GEO	SRR3020312	GSM1973528	mm10	tet-Myc liver tumor	Mus musculus		
RNA-seq	GEO	SRR3020313	GSM1973529	mm10	tet-Myc liver tumor	Mus musculus		
RNA-seq	GEO	SRR3020314	GSM1973530	mm10	tet-Myc liver tumor	Mus musculus		
RNA-seq	GEO	SRR3020315	GSM1973531	mm10	tet-Myc liver tumor	Mus musculus		
RNA-seq	GEO	SRR3020316	GSM1973532	mm10	tet-Myc liver tumor	Mus musculus		
RNA-seq	GEO	SRR3020317	GSM1973533	mm10	tet-Myc liver tumor	Mus musculus		
RNA-seq	GEO	SRR3020318	GSM1973534	mm10	tet-Myc liver tumor	Mus musculus		
RNA-seq	GEO	SRR3020319	GSM1973535	mm10	tet-Myc liver tumor	Mus musculus		
RNA-seq	GEO	SRR3020320	GSM1973536	mm10	tet-Myc liver tumor	Mus musculus		
RNA-seq	GEO	SRR3020321	GSM1973537	mm10	tet-Myc liver tumor	Mus musculus		
RNA-seq	GEO	SRR3020322	GSM1973538	mm10	tet-Myc liver tumor	Mus musculus		
RNA-seq	GEO	SRR3020323	GSM1973539	mm10	tet-Myc liver tumor	Mus musculus		
RNA-seq	GEO	SRR3020324	GSM1973540	mm10	tet-Myc liver tumor	Mus musculus		
RNA-Seq	GEO	SRR8555186	GSM3596803	mm10	Primary B-cells_WT	Mus musculus		untreated
RNA-Seq	GEO	SRR8555187	GSM3596804	mm10	Primary B-cells_WT	Mus musculus		untreated
RNA-Seq	GEO	SRR8555188	GSM3596805	mm10	Primary B-cells_WT	Mus musculus		untreated
RNA-Seq	GEO	SRR8555189	GSM3596806	mm10	Primary B-cells_WT	Mus musculus		untreated
RNA-Seq	GEO	SRR8555190	GSM3596807	mm10	Primary B-cells_WT	Mus musculus		untreated
RNA-Seq	GEO	SRR8555191	GSM3596808	mm10	Primary B-cells_WT	Mus musculus		untreated
RNA-Seq	GEO	SRR8555192	GSM3596809	mm10	Primary B-cells_MycKO	Mus musculus		untreated
RNA-Seq	GEO	SRR8555193	GSM3596810	mm10	Primary B-cells_MycKO	Mus musculus		untreated
RNA-Seq	GEO	SRR8555194	GSM3596811	mm10	Primary B-cells_MycKO	Mus musculus		untreated

Table S1. Cell lines and data sets used in the current study(cont'd)

Assay type	Database	File accession ID	Experiment accession	Genome assembly	Biosample term name	Biosample organism	Experiment target	Biosample treatments
RNA-Seq	GEO	SRR8555195	GSM3596812	mm10	Primary B-cells_MycKO	Mus musculus		untreated
RNA-Seq	GEO	SRR8555196	GSM3596813	mm10	Primary B-cells_MycKO	Mus musculus		untreated
RNA-Seq	GEO	SRR8555197	GSM3596814	mm10	Primary B-cells_MycKO	Mus musculus		untreated
RNA-Seq	GEO	SRR8555198	GSM3596815	mm10	Primary B-cells_WT	Mus musculus		LPS (50 ug/ml) 2h
RNA-Seq	GEO	SRR8555199	GSM3596816	mm10	Primary B-cells_WT	Mus musculus		LPS (50 ug/ml) 2h
RNA-Seq	GEO	SRR8555200	GSM3596817	mm10	Primary B-cells_WT	Mus musculus		LPS (50 ug/ml) 2h
RNA-Seq	GEO	SRR8555201	GSM3596818	mm10	Primary B-cells_WT	Mus musculus		LPS (50 ug/ml) 2h
RNA-Seq	GEO	SRR8555202	GSM3596819	mm10	Primary B-cells_MycKO	Mus musculus		LPS (50 ug/ml) 2h
RNA-Seq	GEO	SRR8555203	GSM3596820	mm10	Primary B-cells_MycKO	Mus musculus		LPS (50 ug/ml) 2h
RNA-Seq	GEO	SRR8555204	GSM3596821	mm10	Primary B-cells_MycKO	Mus musculus		LPS (50 ug/ml) 2h
RNA-Seq	GEO	SRR8555205	GSM3596822	mm10	Primary B-cells_MycKO	Mus musculus		LPS (50 ug/ml) 2h
RNA-Seq	GEO	SRR8555206	GSM3596823	mm10	Primary B-cells_WT	Mus musculus		LPS (50 ug/ml) 4h
RNA-Seq	GEO	SRR8555207	GSM3596824	mm10	Primary B-cells_WT	Mus musculus		LPS (50 ug/ml) 4h
RNA-Seq	GEO	SRR8555208	GSM3596825	mm10	Primary B-cells_WT	Mus musculus		LPS (50 ug/ml) 4h
RNA-Seq	GEO	SRR8555209	GSM3596826	mm10	Primary B-cells_WT	Mus musculus		LPS (50 ug/ml) 4h
RNA-Seq	GEO	SRR8555210	GSM3596827	mm10	Primary B-cells_MycKO	Mus musculus		LPS (50 ug/ml) 4h
RNA-Seq	GEO	SRR8555211	GSM3596828	mm10	Primary B-cells_MycKO	Mus musculus		LPS (50 ug/ml) 4h
RNA-Seq	GEO	SRR8555212	GSM3596829	mm10	Primary B-cells_MycKO	Mus musculus		LPS (50 ug/ml) 4h
RNA-Seq	GEO	SRR8555213	GSM3596830	mm10	Primary B-cells_MycKO	Mus musculus		LPS (50 ug/ml) 4h
RNA-Seq	GEO	SRR8555214	GSM3596831	mm10	Primary B-cells_WT	Mus musculus		LPS (50 ug/ml) 8h
RNA-Seq	GEO	SRR8555215	GSM3596832	mm10	Primary B-cells_WT	Mus musculus		LPS (50 ug/ml) 8h
RNA-Seq	GEO	SRR8555216	GSM3596833	mm10	Primary B-cells_WT	Mus musculus		LPS (50 ug/ml) 8h
RNA-Seq	GEO	SRR8555217	GSM3596834	mm10	Primary B-cells_WT	Mus musculus		LPS (50 ug/ml) 8h
RNA-Seq	GEO	SRR8555218	GSM3596835	mm10	Primary B-cells_MycKO	Mus musculus		LPS (50 ug/ml) 8h
RNA-Seq	GEO	SRR8555219	GSM3596836	mm10	Primary B-cells_MycKO	Mus musculus		LPS (50 ug/ml) 8h
RNA-Seq	GEO	SRR8555220	GSM3596837	mm10	Primary B-cells_MycKO	Mus musculus		LPS (50 ug/ml) 8h
RNA-Seq	GEO	SRR8555221	GSM3596838	mm10	Primary B-cells_MycKO	Mus musculus		LPS (50 ug/ml) 8h
RNA-Seq	GEO	SRR5493432	GSM2595068	mm10	3T9	Mus musculus		0 min MYC-ER activation by 4-hydroxytamoxifen (400nM)
RNA-Seq	GEO	SRR5493433	GSM2595069	mm10	3T9	Mus musculus		10 min MYC-ER activation by 4-hydroxytamoxifen (400nM)
RNA-Seq	GEO	SRR5493434	GSM2595070	mm10	3T9	Mus musculus		20 min MYC-ER activation by 4-hydroxytamoxifen (400nM)
RNA-Seq	GEO	SRR5493435	GSM2595071	mm10	3T9	Mus musculus		30 min MYC-ER activation by 4-hydroxytamoxifen (400nM)
RNA-Seq	GEO	SRR5493436	GSM2595072	mm10	3T9	Mus musculus		1h MYC-ER activation by 4-hydroxytamoxifen (400nM)
RNA-Seq	GEO	SRR5493437	GSM2595073	mm10	3T9	Mus musculus		1.5h MYC-ER activation by 4-hydroxytamoxifen (400nM)
RNA-Seq	GEO	SRR5493438	GSM2595074	mm10	3T9	Mus musculus		2 h MYC-ER activation by 4-hydroxytamoxifen (400nM)
RNA-Seq	GEO	SRR5493439	GSM2595075	mm10	3T9	Mus musculus		4 h MYC-ER activation by 4-hydroxytamoxifen (400nM)
RNA-Seq	GEO	SRR5493440	GSM2595076	mm10	3T9	Mus musculus		8 h MYC-ER activation by 4-hydroxytamoxifen (400nM)
RNA-Seq	GEO	SRR5493441	GSM2595077	mm10	3T9	Mus musculus		12 h MYC-ER activation by 4-hydroxytamoxifen (400nM)
RNA-Seq	GEO	SRR5493442	GSM2595078	mm10	3T9	Mus musculus		16 h MYC-ER activation by 4-hydroxytamoxifen (400nM)
RNA-Seq	GEO	SRR5493454	GSM2595090	mm10	3T9	Mus musculus		0 min MYC-ER activation by 4-hydroxytamoxifen (400nM)
RNA-Seq	GEO	SRR5493455	GSM2595091	mm10	3T9	Mus musculus		10 min MYC-ER activation by 4-hydroxytamoxifen (400nM)
RNA-Seq	GEO	SRR5493456	GSM2595092	mm10	3T9	Mus musculus		20 min MYC-ER activation by 4-hydroxytamoxifen (400nM)
RNA-Seq	GEO	SRR5493457	GSM2595093	mm10	3T9	Mus musculus		30 min MYC-ER activation by 4-hydroxytamoxifen (400nM)
RNA-Seq	GEO	SRR5493458	GSM2595094	mm10	3T9	Mus musculus		1h MYC-ER activation by 4-hydroxytamoxifen (400nM)
RNA-Seq	GEO	SRR5493459	GSM2595095	mm10	3T9	Mus musculus		1.5h MYC-ER activation by 4-hydroxytamoxifen (400nM)
RNA-Seq	GEO	SRR5493460	GSM2595096	mm10	3T9	Mus musculus		2 h MYC-ER activation by 4-hydroxytamoxifen (400nM)
RNA-Seq	GEO	SRR5493461	GSM2595097	mm10	3T9	Mus musculus		4 h MYC-ER activation by 4-hydroxytamoxifen (400nM)
RNA-Seq	GEO	SRR5493462	GSM2595098	mm10	3T9	Mus musculus		8 h MYC-ER activation by 4-hydroxytamoxifen (400nM)
RNA-Seq	GEO	SRR5493463	GSM2595099	mm10	3T9	Mus musculus		12 h MYC-ER activation by 4-hydroxytamoxifen (400nM)
RNA-Seq	GEO	SRR5493464	GSM2595100	mm10	3T9	Mus musculus		16 h MYC-ER activation by 4-hydroxytamoxifen (400nM)
RNA-Seq	GEO	SRR5493476	GSM2595112	mm10	3T9	Mus musculus		0 min MYC-ER activation by 4-hydroxytamoxifen (400nM)
RNA-Seq	GEO	SRR5493477	GSM2595113	mm10	3T9	Mus musculus		10 min MYC-ER activation by 4-hydroxytamoxifen (400nM)
RNA-Seq	GEO	SRR5493478	GSM2595114	mm10	3T9	Mus musculus		20 min MYC-ER activation by 4-hydroxytamoxifen (400nM)
RNA-Seq	GEO	SRR5493479	GSM2595115	mm10	3T9	Mus musculus		30 min MYC-ER activation by 4-hydroxytamoxifen (400nM)
RNA-Seq	GEO	SRR5493480	GSM2595116	mm10	3T9	Mus musculus		1h MYC-ER activation by 4-hydroxytamoxifen (400nM)
RNA-Seq	GEO	SRR5493481	GSM2595117	mm10	3T9	Mus musculus		1.5h MYC-ER activation by 4-hydroxytamoxifen (400nM)
RNA-Seq	GEO	SRR5493482	GSM2595118	mm10	3T9	Mus musculus		2 h MYC-ER activation by 4-hydroxytamoxifen (400nM)
RNA-Seq	GEO	SRR5493483	GSM2595119	mm10	3T9	Mus musculus		4 h MYC-ER activation by 4-hydroxytamoxifen (400nM)

Table S1. Cell lines and data sets used in the current study(cont'd)

Assay type	Database	File accession ID	Experiment accession	Genome assembly	Biosample term name	Biosample organism	Experiment target	Biosample treatments
RNA-Seq	GEO	SRR5493484	GSM2595120	mm10	3T9	Mus musculus		8 h MYC-ER activation by 4-hydroxytamoxifen (400nM)
RNA-Seq	GEO	SRR5493485	GSM2595121	mm10	3T9	Mus musculus		12 h MYC-ER activation by 4-hydroxytamoxifen (400nM)
RNA-Seq	GEO	SRR5493486	GSM2595122	mm10	3T9	Mus musculus		16 h MYC-ER activation by 4-hydroxytamoxifen (400nM)
ENCODE cCREs (encodeCcreCombined)	The UCSC Genome Browser database	encodeCcreCombined.bb		GRCh38		Homo sapiens		
remap2022_crm_macs2_hg38_v1_0.bed.gz	ReMAP2022	https://remap.univ-amu.fr/storage/remap2022/hg38/MACS2/remap2022_crm_macs2_hg38_v1_0.bed.gz		GRCh38		Homo sapiens		

Table S2. 190 genes associated with higher levels of TES-associated MYC+MAX binding and read-through transcription in HCCs vs. livers.

gene_name	HCC			Liver			P value
	Gene_RPKM	DoG_RPKM	DoG_RPKM/Gene_RPKM	Gene_RPKM	DoG_RPKM	DoG_RPKM/Gene_RPKM	
2410002F23Rik	11.34078	0.01426	0.00123	1.53105	0.00000	0.00000	4.60E-03
9530068E07Rik	16.66992	0.09146	0.00542	16.55367	0.04270	0.00275	1.72E-02
Aars	12.45808	0.18122	0.01526	27.31433	0.01128	0.00056	6.49E-06
Acot4	7.93994	0.01391	0.00177	12.61325	0.00805	0.00033	9.17E-03
Adk	49.88304	0.39532	0.00877	114.30119	0.63074	0.00559	3.31E-02
Afmid	1.64394	0.08979	0.05635	20.98295	0.20805	0.00993	4.80E-05
Akap13	2.89655	0.11263	0.03765	3.11615	0.04768	0.01667	3.26E-03
Akt1	16.53958	0.10946	0.00674	5.80536	0.01966	0.00336	4.18E-02
Alkbh6	4.22233	0.09751	0.02420	1.84113	0.01381	0.00778	5.98E-04
Alkbh8	5.70713	0.17645	0.03061	1.26941	0.02068	0.01529	8.81E-03
Ank	1.91810	0.53853	0.28245	2.14291	0.41421	0.19531	4.91E-03
Apoh	65.95812	0.37588	0.00614	474.81591	0.66272	0.00141	8.10E-04
Asb13	11.60138	0.05771	0.00495	9.43275	0.01455	0.00158	4.65E-03
Aspg	13.95176	0.08926	0.00656	33.03140	0.07748	0.00227	6.34E-04
Atp5b	308.36277	0.54225	0.00187	142.65346	0.12272	0.00089	9.26E-03
Atp9b	4.57492	0.13873	0.03073	3.97628	0.06805	0.01871	1.01E-03
Atrn	3.03428	0.11464	0.03838	8.15124	0.09634	0.01334	3.81E-04
BC004004	8.80189	0.03951	0.00466	6.37933	0.00184	0.00027	3.20E-05
Bcar3	3.52909	0.15310	0.04288	7.02469	0.09266	0.01282	1.43E-04
C3	214.34285	0.16265	0.00079	1306.22436	0.26365	0.00021	3.02E-03
C330022C24Rik	5.41556	0.03617	0.00734	23.94696	0.02068	0.00095	1.93E-03
Chd9	3.67687	0.24589	0.06760	3.51365	0.16014	0.04969	2.51E-02
Chid1	7.37203	12.74452	1.73190	4.44363	6.41796	1.45363	6.15E-03
Cluap1	6.19966	0.59598	0.09627	1.95130	0.11533	0.05899	3.67E-03
Commd5	8.00275	0.37842	0.05099	4.21332	0.14459	0.03725	4.60E-02
Coq8b	2.31412	0.01259	0.00496	1.82505	0.00169	0.00084	4.94E-02
Cox17	28.25153	0.01105	0.00042	9.81616	0.00000	0.00000	4.33E-02
Cpb2	31.14404	0.52962	0.01805	57.64553	0.35572	0.00624	2.85E-06
Crebbp	4.19352	0.13751	0.03409	2.33239	0.03921	0.01723	8.74E-03
Crebl2	1.87426	0.08986	0.04779	6.62354	0.07758	0.01154	2.91E-04
Csf1r	5.77080	3.61617	0.66203	3.84525	1.49284	0.44190	5.13E-03
Ctdsp1	10.54117	0.12940	0.01232	15.37208	0.09037	0.00571	5.07E-04
Cul4a	20.57131	0.95883	0.04685	15.69491	0.46734	0.03282	2.62E-02
Cxxc5	8.06895	0.21770	0.02656	5.72521	0.08850	0.01579	1.75E-02
Dbt	9.20462	0.04349	0.00483	13.41807	0.02117	0.00153	4.67E-03
Dedd2	4.86472	0.07345	0.01552	3.10817	0.01042	0.00344	3.88E-04
Dmpk	1.32293	0.17604	0.14341	1.15845	0.09748	0.08097	8.59E-03
Dnaja1	37.95137	16.98592	0.45325	46.72701	5.94671	0.13216	2.17E-11
Dnajc14	4.22559	0.25865	0.06123	4.50589	0.06918	0.01635	5.56E-06
Dnttip1	3.13726	0.23756	0.07471	1.37440	0.02180	0.01671	5.52E-08
Dpm1	4.85415	1.43699	0.29933	4.65783	0.61344	0.13543	2.64E-07
Dst	3.42364	0.05714	0.01683	5.81082	0.01816	0.00365	2.79E-03
Dyrk1a	6.37261	0.21686	0.03375	2.55418	0.05166	0.02182	1.75E-02
Eef1akmt4	7.09084	0.02480	0.00353	2.63222	0.00000	0.00000	1.82E-05

Table S2. 190 genes associated with higher levels of TES-associated MYC+MAX binding and read-through transcription in HCCs vs. livers(cont'd).

gene_name	HCC			Liver			P value
	Gene_RPKM	DoG_RPKM	DoG_RPKM/Gene_RPKM	Gene_RPKM	DoG_RPKM	DoG_RPKM/Gene_RPKM	
Eef1b2	107.33352	1.36229	0.01287	16.52691	0.11634	0.00723	9.05E-04
Eef2	263.48819	0.20858	0.00081	130.94803	0.04154	0.00033	2.01E-03
Egfl7	13.05827	1.37701	0.10741	5.52801	0.06731	0.01292	1.65E-09
Eif4a2	58.20868	15.18731	0.26087	35.78269	0.78064	0.02161	3.24E-17
Eif5	58.69608	2.27125	0.03867	29.67156	0.73348	0.02488	1.07E-04
Elk4	6.09982	0.34530	0.05666	6.46943	0.15819	0.02490	1.80E-06
Eng	2.78456	22.19393	8.61980	7.24490	20.10352	2.83641	6.58E-07
Epb41	12.40302	1.16592	0.09631	10.90067	0.36703	0.03476	5.94E-04
Erbb3	5.86840	0.05741	0.01001	14.82948	0.05558	0.00388	9.96E-03
Etfb	68.97742	0.18590	0.00287	115.15766	0.01786	0.00018	8.42E-04
Evi5	11.80213	0.31051	0.02680	13.48888	0.19742	0.01511	2.38E-04
Fads1	13.53383	0.07673	0.00578	103.77382	0.09996	0.00098	6.04E-03
Farp1	1.10453	0.81479	0.78054	2.43819	0.45461	0.19314	1.23E-10
Fbxl5	6.56279	0.56256	0.08741	2.93184	0.08717	0.03033	3.81E-08
Foxn3	7.55283	0.04824	0.00650	6.34670	0.01978	0.00313	2.25E-02
Gabarapl1	11.90741	0.41434	0.03571	60.42976	0.40099	0.00715	7.55E-06
Gas5	117.35393	2.29247	0.02083	17.92110	0.23462	0.01387	3.21E-02
Gcat	56.06024	4.66023	0.08710	15.25562	0.02215	0.00141	1.56E-08
Gnas	75.01851	0.03658	0.00047	62.06142	0.01234	0.00019	2.46E-02
Gng12	10.23798	0.36515	0.03543	4.20731	0.10122	0.02399	2.45E-02
Golt1a	3.82895	0.01121	0.00330	4.98266	0.00147	0.00027	3.44E-02
Grb2	8.07495	0.09715	0.01182	4.24263	0.02837	0.00649	2.98E-02
Grina	8.62048	0.05344	0.00594	14.94226	0.02145	0.00156	6.60E-04
H13	18.63147	0.13058	0.00717	22.45215	0.06245	0.00321	8.26E-03
Herc4	5.73196	0.21062	0.03722	5.07393	0.13059	0.02522	3.13E-02
Hmgcs2	7.21698	0.25264	0.03700	76.77754	0.46113	0.00642	2.28E-06
Hook3	3.17220	0.10103	0.03153	4.50197	0.07941	0.01751	1.76E-02
Hsd11b1	4.01656	0.02421	0.00687	168.72190	0.14974	0.00086	3.44E-03
Hsd17b2	9.78746	0.06320	0.00660	41.05146	0.12799	0.00292	3.32E-03
Ifnar2	2.45068	0.11209	0.04547	1.08456	0.02361	0.02459	4.32E-02
Ip6k1	5.10926	0.01592	0.00314	6.70319	0.00545	0.00083	1.77E-02
Irf6	2.33426	0.13290	0.06128	5.64007	0.04310	0.00771	1.34E-05
Jam2	1.27642	0.45734	0.37189	1.27520	0.23896	0.19733	5.56E-07
Jmjd8	18.86097	2.25497	0.12040	13.99545	1.25238	0.08701	2.59E-04
Kansl3	3.98085	0.10719	0.02743	2.50058	0.04090	0.01595	2.49E-02
Kif13b	3.96629	0.28026	0.07017	5.94631	0.29351	0.05037	3.47E-02
Klf9	6.33103	0.42799	0.06843	9.89090	0.28537	0.02948	3.64E-07
Lipc	12.17822	0.14504	0.01278	39.45775	0.03881	0.00097	1.42E-04
Litaf	17.44704	0.27388	0.01576	12.08337	0.08777	0.00746	6.17E-05
Lrba	2.60069	0.08241	0.03225	1.68085	0.02382	0.01485	9.12E-03
Lrrc8a	3.75985	0.26126	0.07116	4.48840	0.13945	0.03127	2.47E-05
Malat1	239.30118	0.85539	0.00365	450.23848	0.29439	0.00066	4.27E-08
Matr3	32.40263	1.14871	0.03515	7.52704	0.16688	0.02237	2.52E-03
Mbnl1	18.78336	0.74499	0.03964	12.69409	0.33937	0.02569	4.39E-03

Table S2. 190 genes associated with higher levels of TES-associated MYC+MAX binding and read-through transcription in HCCs vs. livers(cont'd)).

gene_name	HCC			Liver			P value
	Gene_RPKM	DoG_RPKM	DoG_RPKM/Gene_RPKM	Gene_RPKM	DoG_RPKM	DoG_RPKM/Gene_RPKM	
Mccc1	2.78989	0.66021	0.23775	3.45600	0.47701	0.13678	1.18E-04
Mgll	13.41916	0.08420	0.00633	31.22481	0.06074	0.00200	2.39E-04
Mmd	3.02775	0.02138	0.00683	11.59133	0.03091	0.00266	2.59E-02
Mrpl15	3.59307	0.83807	0.23513	1.10338	0.12251	0.11614	3.82E-04
Msl1	4.62548	0.10253	0.02159	3.13617	0.02948	0.00987	8.52E-03
Mtor	5.57465	0.02817	0.00509	4.10461	0.00720	0.00156	1.69E-02
Ndufb9	84.82118	0.32195	0.00413	108.30192	0.21495	0.00214	1.36E-02
Nek7	9.81215	0.18031	0.01830	8.61428	0.08129	0.00932	1.44E-02
Nfib	4.49735	0.34477	0.07773	9.66122	0.26678	0.02808	2.79E-09
Nipsnap1	89.87719	0.52639	0.00595	53.24608	0.07275	0.00137	4.63E-07
Nr3c2	1.40740	0.22085	0.16221	1.90801	0.07397	0.04326	7.58E-07
Nsmf	2.68291	0.14348	0.05482	14.08314	0.19722	0.01420	2.48E-06
Nucks1	34.49483	0.36951	0.01071	10.74348	0.07056	0.00684	4.73E-03
Numb	2.30352	1.32581	0.57393	2.62278	0.25995	0.10042	5.44E-10
Oaz1	68.36889	0.19938	0.00300	69.67492	0.03563	0.00052	1.77E-06
Oaz1-ps	50.00989	0.38097	0.00823	53.12362	0.10726	0.00235	2.12E-04
Pcsk6	5.07658	0.62130	0.12419	12.94952	0.09845	0.00829	1.38E-09
Pex6	9.37149	2.14509	0.23413	11.67859	1.32602	0.13515	1.20E-04
Pfdn2	10.74426	0.23371	0.02203	3.94839	0.05461	0.01360	1.43E-02
Phkb	3.99376	0.40147	0.10088	3.46376	0.26239	0.07712	4.98E-03
Pick1	6.35381	0.02885	0.00466	1.88852	0.00000	0.00000	2.22E-03
Pik3c2a	14.05468	0.95924	0.06835	5.52838	0.16583	0.03036	7.95E-05
Pik3c2g	1.25602	0.03832	0.03939	1.78056	0.02488	0.01348	3.08E-02
Pknox1	2.56655	0.10710	0.04366	1.69561	0.03590	0.02060	1.84E-02
Pnkd	4.30315	0.03632	0.00776	7.49645	0.01116	0.00138	6.90E-04
Pnpla7	4.59443	0.11631	0.02528	38.07515	0.17683	0.00489	1.21E-06
Ppp1r21	3.64693	0.19434	0.05279	2.16074	0.04693	0.02067	3.48E-05
Prr14	2.89118	0.11486	0.03902	4.52120	0.10021	0.02341	2.20E-02
Ptbp1	67.99520	0.36709	0.00537	11.42549	0.02239	0.00192	2.08E-05
Ptprf	10.99600	0.67180	0.06108	18.08228	0.62899	0.03515	1.96E-07
Rabggtb	25.31230	1.40123	0.05613	6.02275	0.13963	0.02355	2.80E-06
Ramp2	16.76655	0.16949	0.01019	12.79984	0.04335	0.00331	3.45E-04
Rara	3.33304	0.02708	0.00774	3.06419	0.00079	0.00025	1.69E-03
Rc3h1	6.12920	0.35182	0.05679	2.95602	0.10572	0.03390	2.76E-03
Repin1	4.91507	0.09606	0.01979	5.67922	0.01218	0.00208	9.12E-09
Rhou	2.65160	0.07441	0.02784	10.88760	0.06135	0.00704	8.72E-06
Rit1	4.22707	0.15809	0.03819	2.89265	0.07743	0.02661	3.77E-02
Rnf130	2.96437	0.07620	0.02647	8.95353	0.08341	0.00925	2.01E-03
Rnf14	5.63259	0.14671	0.02640	7.30376	0.09435	0.01320	7.28E-04
Rpl17	341.86199	1.92865	0.00577	68.48556	0.26236	0.00389	8.60E-03
Rpl21	115.56349	0.86557	0.00810	22.87647	0.11202	0.00513	2.83E-02
Rpl27a	290.31906	2.07393	0.00744	57.03651	0.27439	0.00479	6.44E-04
Rpl37	82.75293	2.29689	0.03039	15.86345	0.29958	0.02032	2.18E-02
Rpl4	547.56298	0.70036	0.00132	94.61602	0.03237	0.00035	9.64E-07

Table S2. 190 genes associated with higher levels of TES-associated MYC+MAX binding and read-through transcription in HCCs vs. livers(cont'd).

gene_name	HCC			Liver			P value
	Gene_RPKM	DoG_RPKM	DoG_RPKM/Gene_RPKM	Gene_RPKM	DoG_RPKM	DoG_RPKM/Gene_RPKM	
Rpl6	159.99727	0.99485	0.00676	32.42205	0.09607	0.00306	4.53E-03
Rps20	232.91263	2.00933	0.00902	36.81532	0.14260	0.00402	6.87E-05
Rpsa	299.98263	2.07477	0.00735	52.01091	0.16088	0.00353	7.20E-04
Rusf1	6.58994	0.23601	0.03614	2.89391	0.05266	0.01879	8.19E-04
Ryk	12.97168	0.44937	0.03535	4.22666	0.07271	0.01782	8.65E-04
Sap18	3.08975	4.14444	1.34454	1.86405	0.10423	0.05812	1.57E-13
Serpinc1	52.59892	3.45409	0.06733	169.05848	2.25578	0.01346	1.40E-09
Sft2d1	5.47567	0.00773	0.00138	4.16691	0.00000	0.00000	1.86E-02
Sgms1	4.93866	0.15574	0.03141	1.88629	0.03267	0.01812	3.48E-02
Shb	1.09890	0.04153	0.03557	3.78296	0.05010	0.01423	2.45E-02
Sirt7	3.91522	0.11369	0.02884	6.66898	0.12695	0.01808	1.73E-02
Slc16a1	69.20914	0.30102	0.00451	22.53791	0.01485	0.00074	2.93E-08
Slc25a16	2.98584	0.07428	0.02503	8.10769	0.02770	0.00356	2.02E-07
Slc25a47	10.09411	0.21148	0.02354	76.27969	0.45370	0.00581	3.97E-05
Slc35e2	4.64409	0.48176	0.10549	12.69505	0.24359	0.02025	4.56E-09
Slc6a6	15.13141	0.14463	0.00946	10.86325	0.04475	0.00388	5.92E-05
Smap2	4.30959	0.22924	0.05299	3.58705	0.12563	0.03596	1.33E-02
Smo	5.68280	0.14750	0.02590	1.94513	0.02003	0.01096	3.20E-03
Smurf1	1.10130	0.03195	0.03122	1.44364	0.01544	0.01210	1.38E-02
Snhg12	27.24115	3.63577	0.13356	15.14413	0.40515	0.02927	2.03E-13
Snora16a	27.44810	2.47587	0.10015	9.24959	0.22397	0.03005	1.13E-05
Snora20	2.69603	2.50515	0.96650	8.78256	1.03801	0.12886	1.27E-09
Snora3	8.16171	3.25818	0.41059	2.00327	0.45206	0.25707	2.89E-03
Snora44	26.90402	2.43713	0.10762	5.25847	0.18551	0.04038	1.71E-03
Snora61	112.60862	2.47001	0.02408	24.14663	0.20694	0.01037	2.31E-03
Snord22	688.30769	2.39465	0.00476	368.46463	0.06908	0.00024	3.01E-03
Srd5a1	2.98289	0.04570	0.01572	14.29234	0.05513	0.00373	2.39E-04
Srebf1	11.87667	2.22253	0.18983	50.59409	0.57400	0.01370	7.13E-10
Srf	3.89285	0.92646	0.24268	1.37170	0.21125	0.14842	6.57E-03
Ssh2	3.15768	0.08937	0.02855	3.02354	0.05123	0.01726	1.42E-02
Stat1	4.52154	0.24369	0.05411	4.66229	0.14029	0.03095	1.63E-04
Taok3	2.48144	0.06538	0.02731	1.97975	0.02714	0.01513	1.19E-02
Tapbp	4.38145	0.23626	0.05621	7.55369	0.10315	0.01344	6.68E-06
Tapt1	7.77002	0.30905	0.03989	11.72817	0.30574	0.02634	2.40E-03
Thtpa	3.29474	3.11874	0.97613	3.44360	0.63914	0.20642	1.98E-09
Tlcd1	14.71093	4.76327	0.32753	7.23593	1.65309	0.23545	9.65E-04
Tmem123	9.29884	0.44816	0.04934	5.93812	0.02881	0.00514	2.28E-07
Tmem184b	2.94674	0.21777	0.07342	2.13348	0.10680	0.04903	1.62E-02
Tmem38b	5.75361	0.07184	0.01263	15.11911	0.07983	0.00577	1.68E-03
Tmem62	1.22568	0.01106	0.00875	2.33360	0.00385	0.00209	1.96E-02
Tnpo2	7.84798	0.34012	0.04403	2.84040	0.04374	0.01913	2.26E-03
Tpt1	399.02910	1.26237	0.00323	171.18316	0.25973	0.00159	9.11E-06
Traf7	5.01921	0.68765	0.13724	1.06676	0.05643	0.05802	2.69E-05
Tymp	12.93516	17.19967	1.32454	24.56404	4.72316	0.21049	9.72E-16

Table S2. 190 genes associated with higher levels of TES-associated MYC+MAX binding and read-through transcription in HCCs vs. livers(cont'd).

gene_name	HCC			Liver			P value
	Gene_RPKM	DoG_RPKM	DoG_RPKM/Gene_RPKM	Gene_RPKM	DoG_RPKM	DoG_RPKM/Gene_RPKM	
Uba7	1.45551	0.01877	0.01524	1.55472	0.00244	0.00154	4.51E-02
Ubqln1	19.62581	0.36670	0.01831	13.86032	0.09122	0.00658	1.07E-05
Ugt1a10	12.09358	0.73280	0.06371	168.72442	1.09888	0.00694	3.06E-08
Ugt1a6a	12.09358	0.73280	0.06371	168.72442	1.09888	0.00694	3.06E-08
Ugt1a7c	12.09358	0.73280	0.06371	168.72442	1.09888	0.00694	3.06E-08
Uso1	4.87062	0.07419	0.01566	8.28451	0.05000	0.00648	2.27E-02
Vps13b	5.33378	0.99298	0.18464	3.42649	0.25932	0.07595	4.57E-07
Vps35l	4.80230	13.80961	2.90414	1.85689	2.83377	1.55445	3.32E-10
Whamm	2.89285	0.28749	0.10000	11.92157	0.28676	0.02388	2.43E-08
Zfand3	4.94925	0.23571	0.04804	7.86967	0.16858	0.02103	9.48E-05
Zfas1	43.26804	1.72950	0.04148	2.97412	0.03629	0.01174	4.48E-05
Zfp444	4.31142	0.02822	0.00697	1.79141	0.00460	0.00251	4.57E-02
Zfp524	1.95369	0.19389	0.09868	1.81303	0.09346	0.05310	5.56E-03
Zfp710	3.24251	1.51147	0.47205	1.15344	0.21484	0.18745	3.38E-08

Table S3. Oligonucleotides used to create pDG458 gRNA constructs for CRISPR-Cas9 targeting of TES-associated E boxes and PCR primers for amplifying regions flanking the targeted E boxes.

Crispr-Cas9 targeting site	Purpose	oligo Name	Sequance
Rps19_TES	for pDG458 construct	Rps19_Fw_P2_gR	accgCTAAGGCTTTGAGAATCACAgt
		Rps19_Fw_P2_gR_rc	taaaacTGTGATTCTCAAAGCCTTAG
	amplify E box flanking region	Rps19_TES_fwd	AGTGCGGTTGAGCAGATTTA
		Rps19_TES_rev	CAGTTGGCAGTTCCTGAAGA
Tspo/Ttll12_TES	for pDG458 construct	Tspo_fw_P1_gR	caccgTGCCCACCTTGAGCACGTGC
		Tspo_fw_P1_gR_rc	aaacGCACGTGCTCAAGGTGGGCAc
	amplify E box flanking region	Tspo_TES_fw	GGTCTCTGGCTTGTGCTTAT
		Tspo_TES_rev	CAAGACAGCTCACCGAGTAAA
Git1/Trp53i13_TES	for pDG458 construct	Git1_rv_P1_gR	caccgCTCTGCGACCGCTGCCACGT
		Git1_rv_P1_gR_rc	aaacACGTGGCAGCGGTCGCAGAGc
	amplify E box flanking region	Git1_TES_fw	TTCCTTGCCCTTGACTTCTC
		Git1_TES_rev	CCATGTCCTCTCTCTGCAATC
Hsp90aa1_TES	for pDG458 construct	Hsp90aa1_fw_P1_gR	caccgCATGAATCAGGCACGTGCCT
		Hsp90aa1_fw_P1_gR_rc	aaacAGGCACGTGCCTGATTCATGc
	amplify E box flanking region	Hsp90aa1_TES_fw	CCCGAAACAAGTGCTTTGATAC
		Hsp90aa1_TES_rev	CTACTTTCCTTCCCTCCCTTTG
Dyrk3_TES	for pDG458 construct	Dyrk3_fw_P1_gR	caccgAAAAACTGAACTGGGTCACG
		Dyrk3_fw_P1_gR_rc	aaacCGTGACCCAGTTCAGTTTTTc
	amplify E box flanking region	Dyrk3_TES_fw	CAGGGCTTCCACACTGATAA
		Dyrk3_TES_rev	TGACTCCTGTGGGCATTAAG

Table S4. qRT-PCR primers used to quantify gene expression and DoG expression in WT and KO fibroblasts (Figure 7B-7D).

Name of Gene	NCBI Gene ID	Assay	Reverse transcription primer	RT-PCR primers
Tspo	12257	Total gene expression	Random hexamers	Fwd: 5 '-CGCTTGCTGTACCCTTACC-3'
				Rev: 5'-CCAGAGTTATCACGCCATACAT-3'
		Expression of DoG	TES_RT: 5'-CAAGACAGCTCACCGAGTAAA-3'	Fwd: 5 '-CGCTTGCTGTACCCTTACC-3'
				Rev: 5'-CCAGAGTTATCACGCCATACAT-3'
Ttll12	223723	Total gene expression	Random hexamers	Fwd: 5'-CCAAATCCTGGAGGTGAACTT-3'
				Rev: 5'-TCAGTCTCGTCCAGAAACAAAG-3'
		Expression of DoG	TES_RT:5'-GGTCTCTGGCTTGTGCTTAT-3'	Fwd: 5'-CCAAATCCTGGAGGTGAACTT-3'
				Rev: 5'-TCAGTCTCGTCCAGAAACAAAG-3'
Git1	216963	Total gene expression	Random hexamers	Fwd: 5'-CGGCTTCAGAGCGAGTG-3'
				Rev: 5'-CAGCCTTGGCGATGTCATA-3'
		Expression of DoG	TES_RT: 5'-TTCCTTGCCCTTGACTTCTC-3'	Fwd: 5'-CGGCTTCAGAGCGAGTG-3'
				Rev: 5'-CAGCCTTGGCGATGTCATA-3'
Trp53i13	216964	Total gene expression	Random hexamers	Fwd: 5'-CCTCGATCAGTGTTGTGAAGAG-3'
				Rev: 5'-CTGTGAAGAGGCCAAGGAAA-3'
		Expression of DoG	TES_RT: 5'-CCATGTCCTCTCTCTGCAATC-3'	Fwd: 5'-CCTCGATCAGTGTTGTGAAGAG-3'
				Rev: 5'-CTGTGAAGAGGCCAAGGAAA-3'
Dyrk3	226419	Total gene expression	Random hexamers	Fwd: 5'-CCCACCCTATTCGGACACATT-3'
				Rev: 5'-TGAAACAGTTGTTCCACCTTCAT-3'
		Expression of DoG	TES_RT: 5'-CAGGGCTTCCACACTGATAA-3'	Fwd: 5'-CCCACCCTATTCGGACACATT-3'
				Rev: 5'-TGAAACAGTTGTTCCACCTTCAT-3'
Rps19	20085	Total gene expression	Random hexamers	Fwd: 5'-CAGCAGGAGTTCGTCAGAGC-3'
				Rev: 5'-CACCCATTCGGGGACTTTCA-3'
		Expression of DoG	TES_RT: 5'-CAGTTGGCAGTTCCTGAAGA-3'	Fwd: 5'-CAGCAGGAGTTCGTCAGAGC-3'
				Rev: 5'-CACCCATTCGGGGACTTTCA-3'
Hsp90aa1	15519	Total gene expression	Random hexamers	Fwd: 5'-TGTTGCGGTACTACACATCTGC-3'
				Rev: 5'-GTCCTTGGTCTCACCTGTGATA-3'
		Expression of DoG	TES_RT: 5'-CTACTTTCCTTCCCTCCCTTTG-3'	Fwd: 5'-TGTTGCGGTACTACACATCTGC-3'
				Rev: 5'-GTCCTTGGTCTCACCTGTGATA-3'
Tbp	21374	Internal conrol for total gene expression	Random hexamers	Fwd: 5’-CCCCACAACTCTTCCATTCT-3’
				Rev: 5'-5’-GCAGGAGTGATAGGGGTCAT-3’
		Internal conrol for expression of DoG	TES_RT: 5'-GTGGTCTTCCTGAATCCCTTTA-3'	Fwd: 5’-CCCCACAACTCTTCCATTCT-3’
				Rev: 5'-5’-GCAGGAGTGATAGGGGTCAT-3’

Table S5. qRT-PCR primers used to quantify TES-TSS region interactions(Figure 7F).

Name of Gene	NCBI Gene ID	Amplicon length	RT-PCR primers
Tspo	12257	58 bp	5’-CCTTGGGTTGGTAGTGTGGA-3’
			5’-AAGAGAGACAGCCTGTGGAC-3’
Ttll12	223723	90 bp	5’-CCGTCCGCTTAAATCCTGC-3’
			5’-CCTTGGGTTGGTAGTGTGGA-3’
Git1	216963	205 bp	5’-TCCGAATCAGGGCCACTATC-3’
			5’-CAGAGCAGCAGTGACTTGTG-3’
Trp53i13	216964	158 bp	5’-TCACAGACATCCACTTGCCT-3’
			5’-TCCGAATCAGGGCCACTATC-3’
Dyrk3	226419	144 bp	5’-GCCCAGCCTCGTCTTGTA-3’
			5’-TCACAAGCTTTTCCTGTGGC-3’
Rps19	20085	102 bp	5’-CAAAATGGGCGGGTCTGTAA-3’
			5’-ATGCACTTCACACTGGGAGA-3’
Hsp90aa1	15519	104 bp	5’-TGAGTACACTGTCCCTGTCT-3’
			5’-GGGGCATTAAGTAGAAACAGTTT-3’
Mlx (Control)	21428	76 bp	5'-AGTCCGCTGGCTTGTTT-3'
			5'-TTGACCCAAGGGTCCTC-3'
			prob: 56-FAM/CGGTTCGGT/ZEN/AGGTTCACGATGACG/3IAB kFQ/

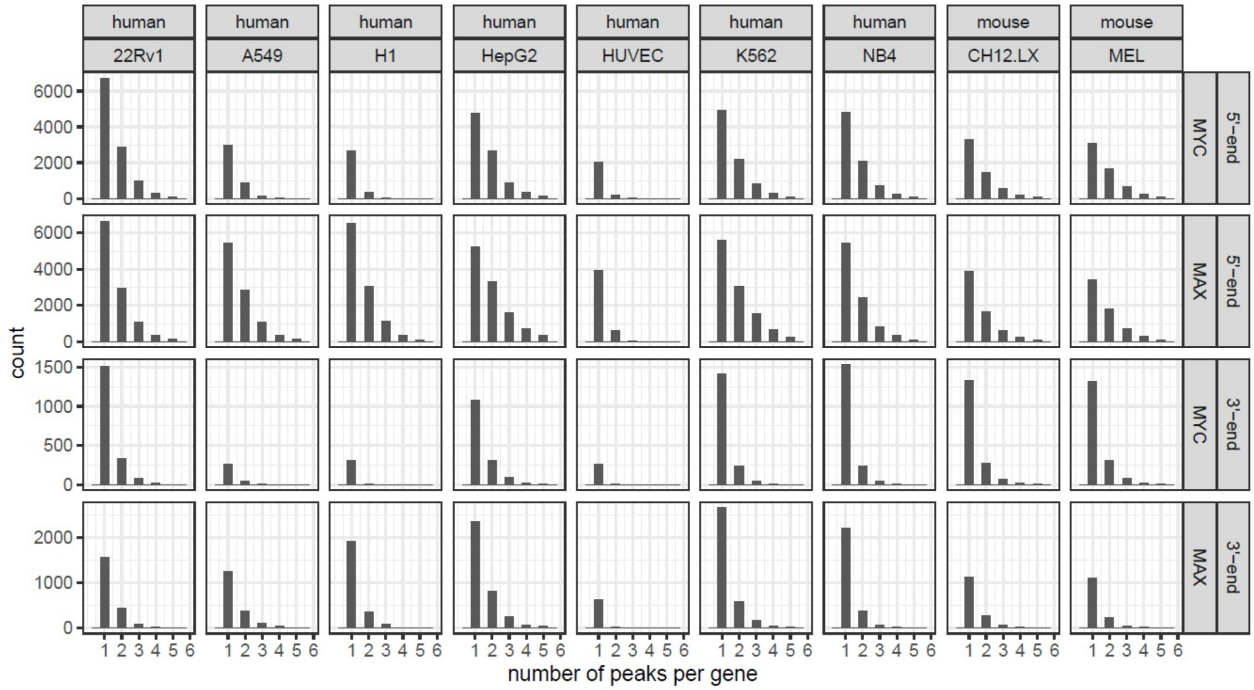

Figure S1. Distribution of MYC and/or MAX binding sites residing within +/- 2.5 kb of TSSs and TESs. Results of MYC and MAX binding were obtained from the ENCODE and GEO databases (Table S1). AnnotatePeakInBatch (ChIPpeakAnno Version 3.6.5) was used to assign binding sites.

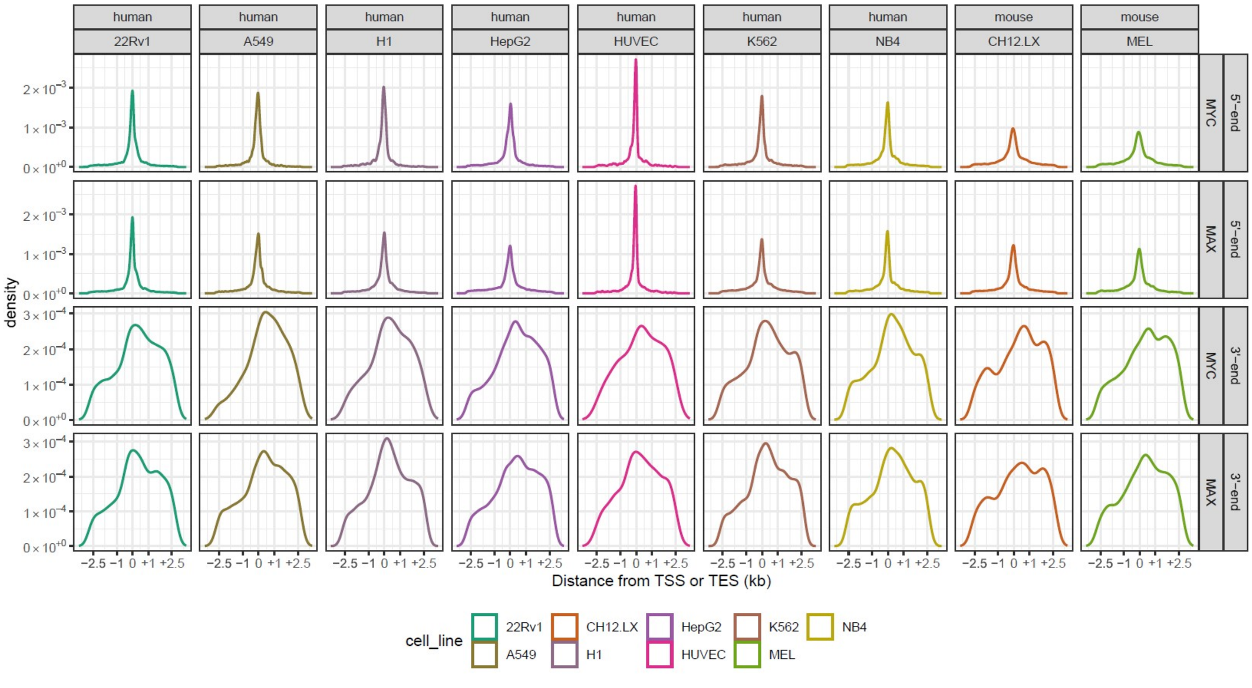

Figure S2. Binding of MYC and MAX around TSSs and TESs of genes for the individual cell lines shown in Figure 1B.

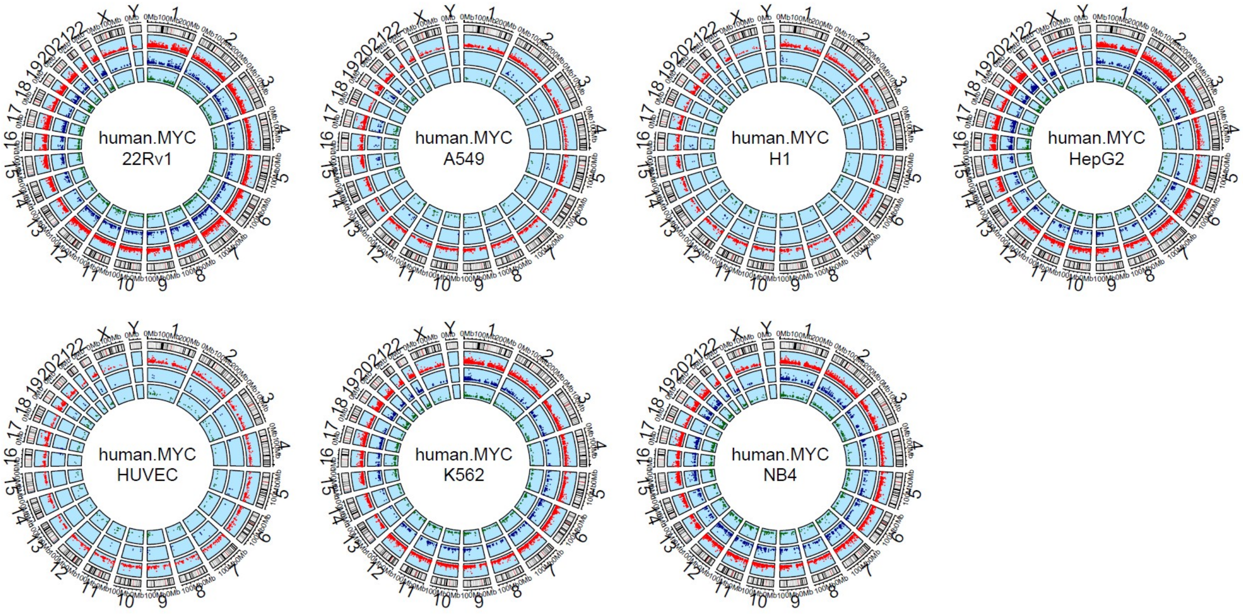

Figure S3. Circos plots showing the chromosomal locations of human genes that bind MYC only in the vicinity of TSSs and/or TESs in each of the 7 cell lines used in this study. Red dots: locations of genes associated with MYC binding near TSSs only; green dots: locations of genes associated with MYC binding near TESs only; blue dots: locations of genes associated with MYC binding near both sites.

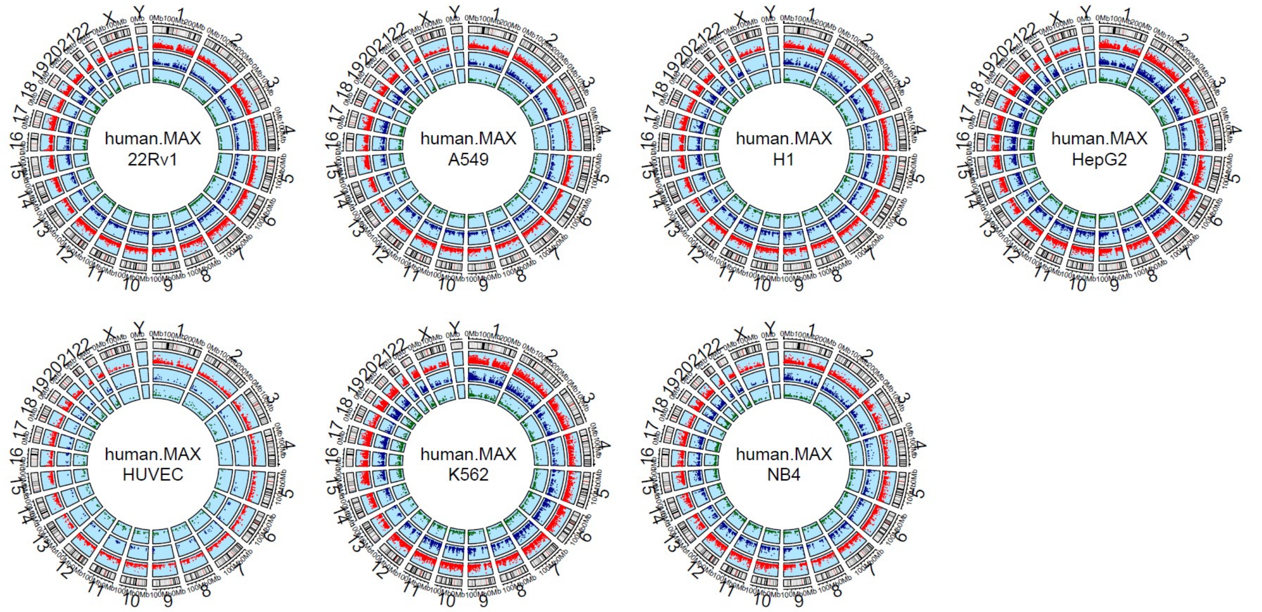

Figure S4. Circos plots showing the chromosomal locations of human genes that bind MAX only in the vicinity of TSSs and/or TESs in each of the 7 cell lines used in this study. Red dots: locations of genes associated with MAX binding near TSSs only; green dots: locations of genes associated with MAX binding near TESs only; blue dots: locations of genes associated with MAX binding near both sites.

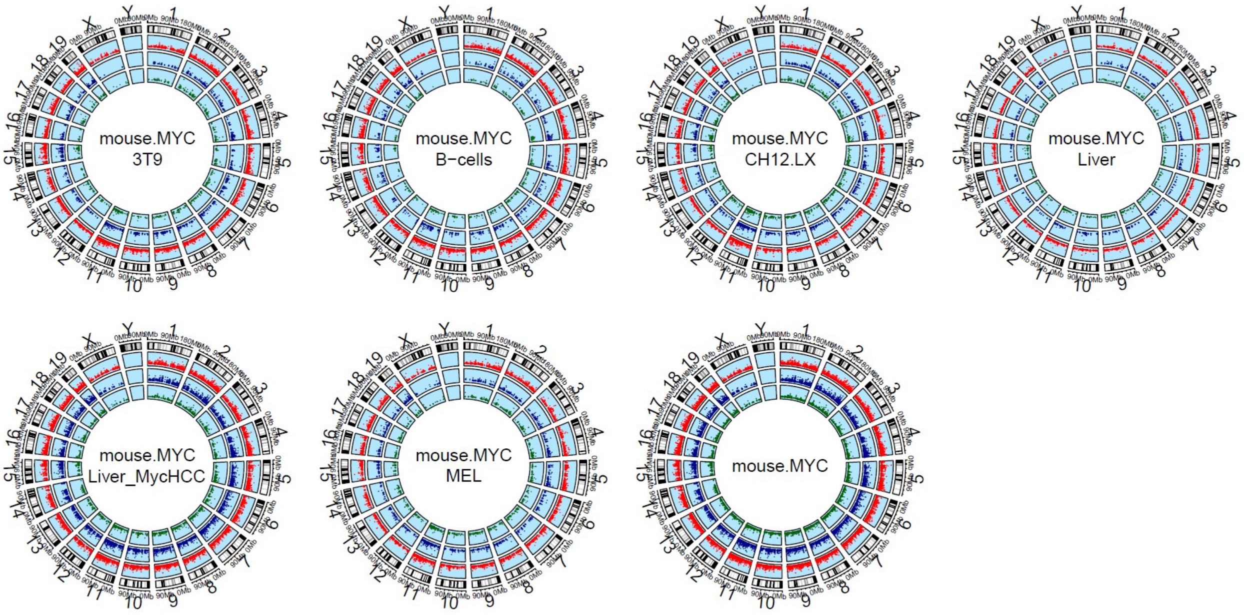

Figure S5. Circos plots showing the chromosomal locations of mouse genes that bind MYC only in the vicinity of TSSs and/or TESs in each of the tissues and cell lines used in this study. Red dots: locations of genes associated with MYC binding near TSSs only; green dots: locations of genes associated with MYC binding near TESs only; blue dots: locations of genes associated with MYC binding near both sites. The last diagram shows the combined results similar to that shown for human genes in Figure 1F.

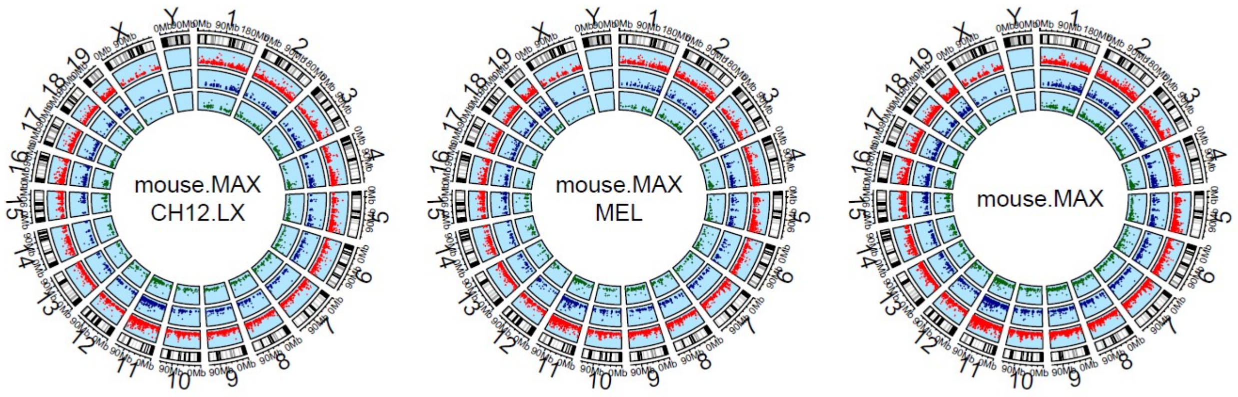

Figure S6. Circos plots showing the chromosomal locations of mouse genes that bind MAX only in the vicinity of TSSs and/or TESs in each of the cell lines used in this study. Red dots: locations of genes associated with MAX binding near TSSs only; green dots: locations of genes associated with MAX binding near TESs only; blue dots: locations of genes associated with MAX binding near both sites. The last diagram shows the combined results similar to that shown for human genes in Figure 1F.

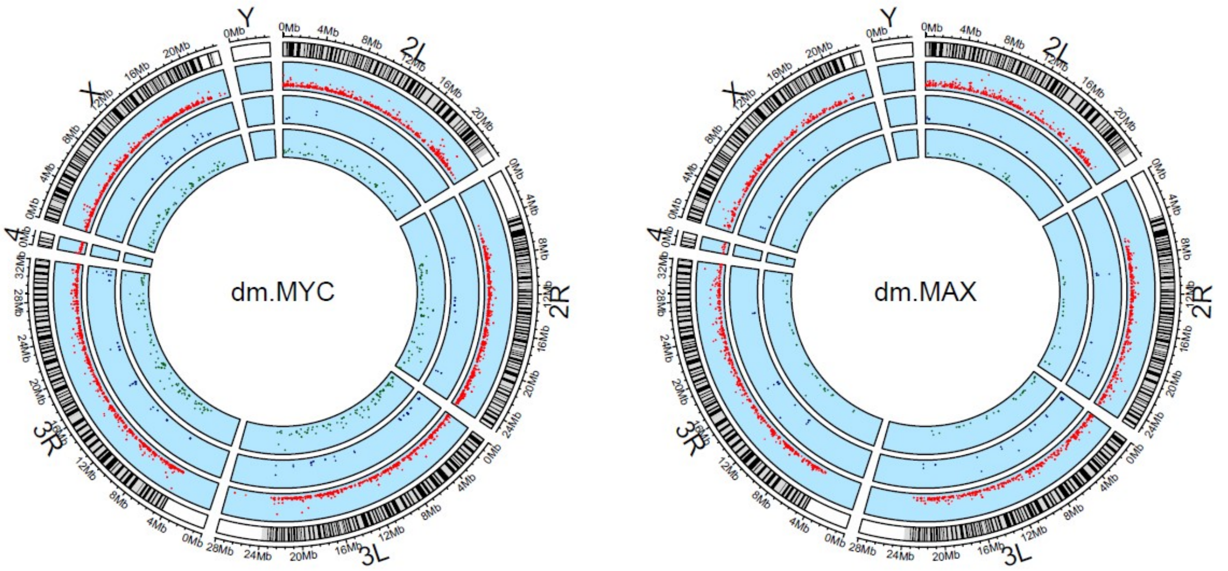

Figure S7. Circos plots showing the chromosomal locations of *D. melanogaster* genes that bind MAX only in the vicinity of TSSs and/or TESs. Gene locations are based on ChIPseq results obtained from third instar larvae.[8a] Red dots: locations of genes associated with dMYC or dMAX binding around TSSs only; green dots: locations of genes associated with dMYC or dMAX binding around TESs only; blue dots: locations of genes associated with dMYC or dMAX binding at both TSSs and TESs.

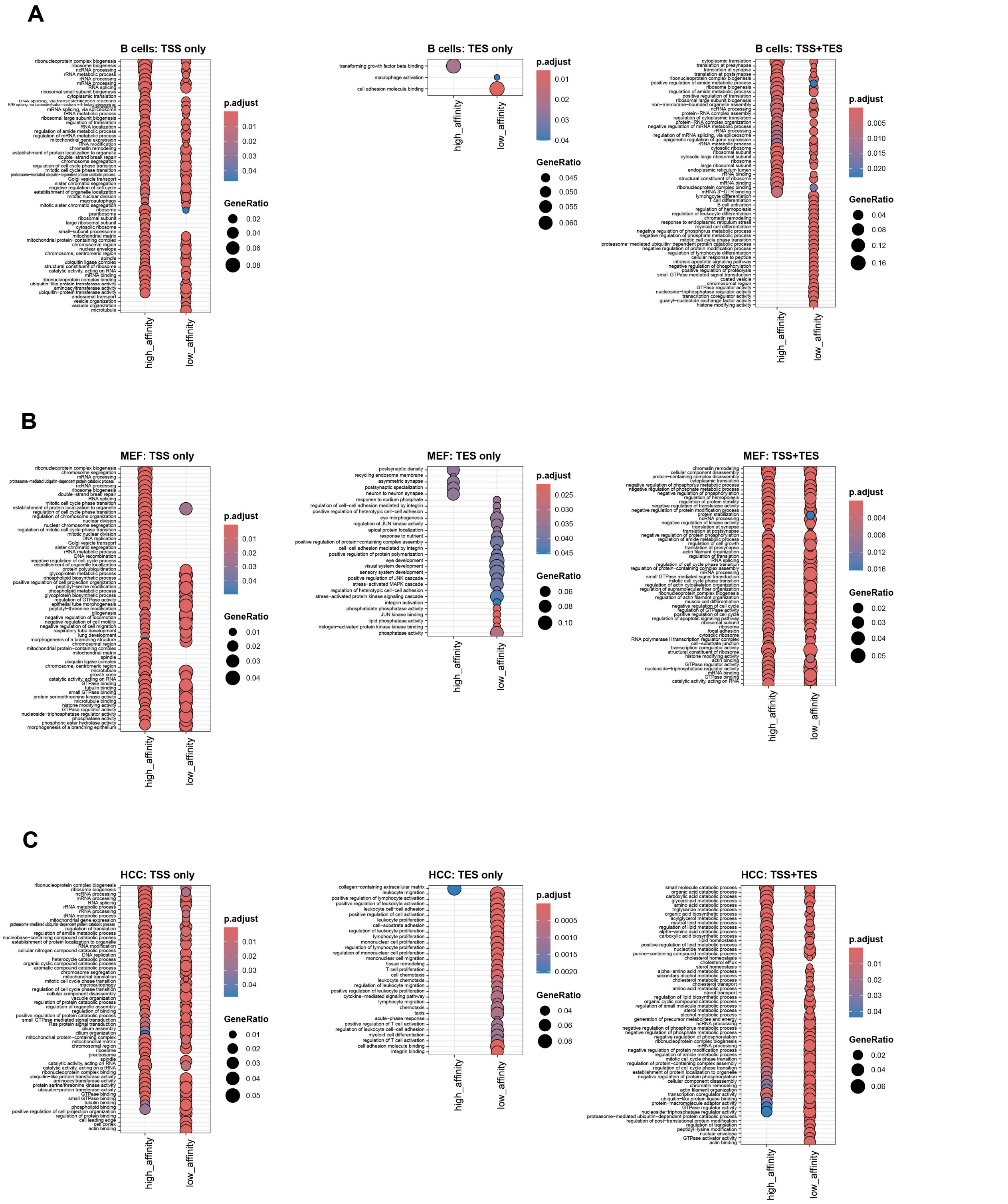

Figure S8. High- and low-affinity MYC binding sites at TESs are associated with different functional categories of genes. Based on the MYC binding site affinities depicted in Fig. 3A-I,

over-representation analysis from the MSigDB database's C5 GO collection was performed as described in Fig. 3J. Genes that bound MYC exclusively in the vicinity of TSSs, exclusively in the vicinity of TESs or in the vicinity of both TSSs and TESs were categorized into functional groups, the most significant of which are indicated.
(A). Analyses performed Murine B cells in which physiologic levels of Myc over-expression were induced by LPS.
(B). Analyses performed on MEFs in which Myc over-expression was induced by MycER induction in response to 4OHT.
(C). Analyses performed on HCCs induced by the doxycycline-mediate induction of MYC.
The paucity of functional categories around high affinity TES-only sites reflects the small number of genes representing such sites and/or their exclusion from defined functional categories.
See File S1 for a complete list of the genes depicted in each of the above categories

Git1/Trp53i13_TES_E-box

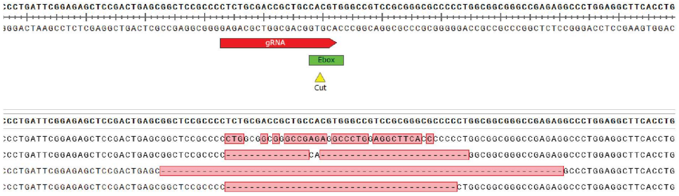

Tspo/Ttll12_TES_E-box

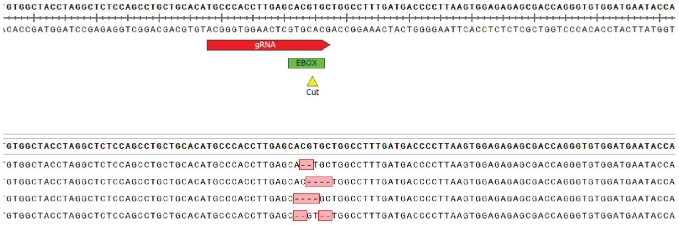

Hsp90aa1_TES_E-box

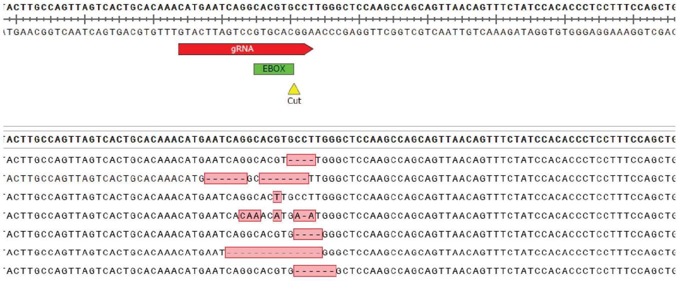

Dyrk3_TES_E-box

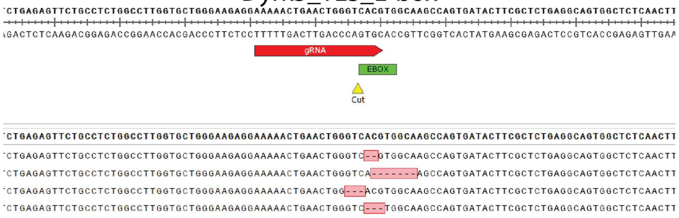

Rps19_TES_E-box

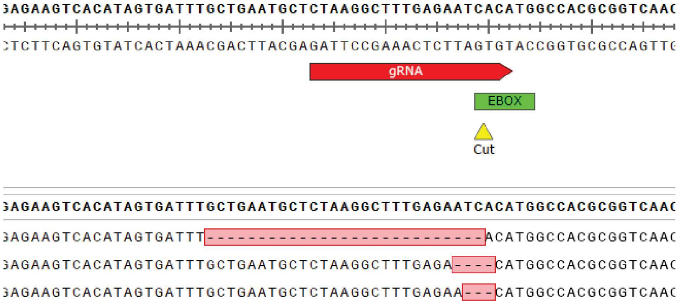

Figure S9. Mutagenesis of TES-associated E boxes identified as sites of MYC/MAX binding in immortalized murine fibroblasts.
The top portion of each panel displays the WT gene sequence surrounding the TES-associated E box, and the gRNA used for Crispr-mediated editing. Beneath this are the mutant sequences identified subsequent to Crispr-Cas9 targeting of the sites. In keeping with the fact that NIH3T3 cells are hyperdiploid,[46] all clones contained between 3 and 6 different mutant alleles.

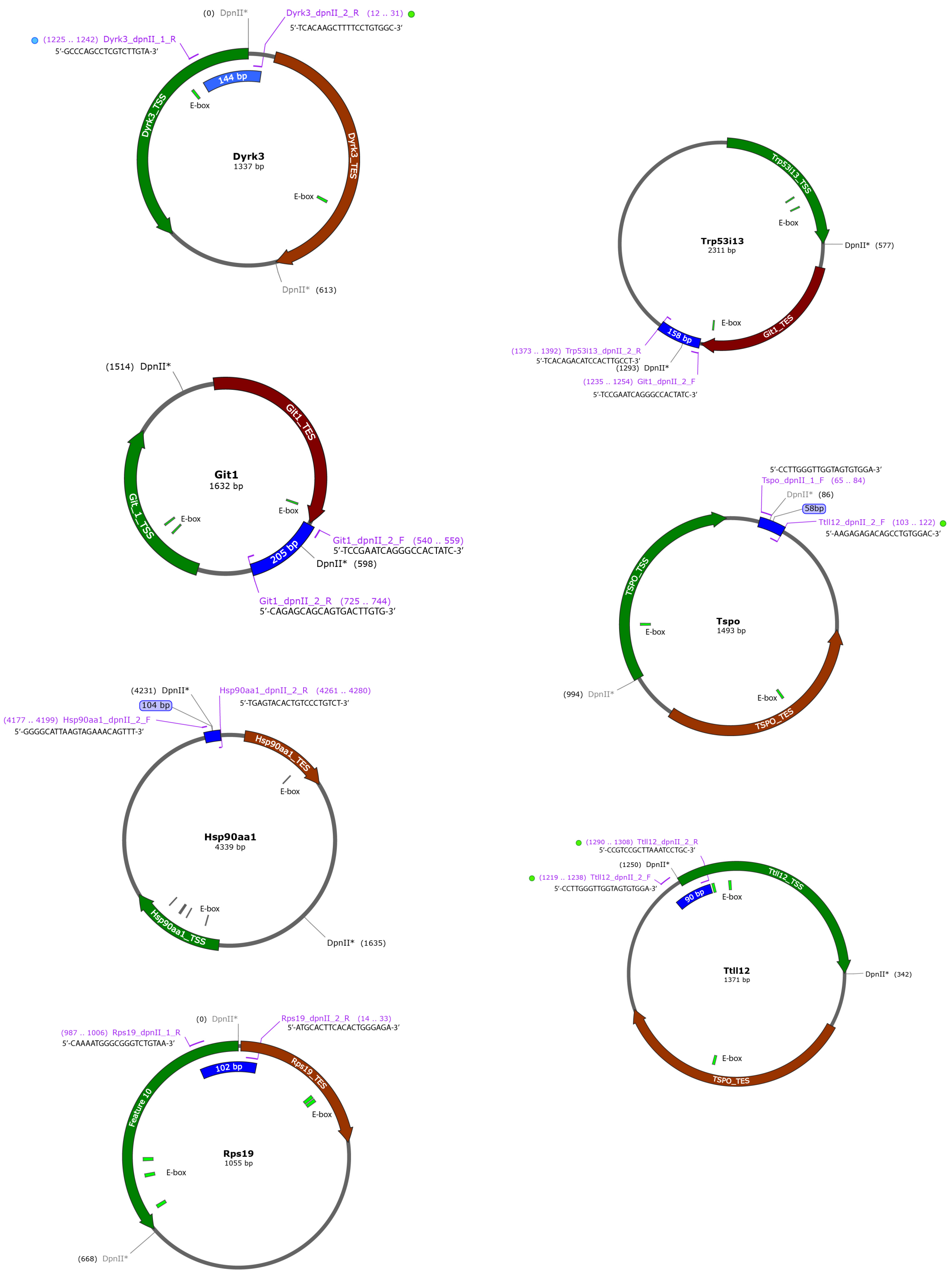

Figure S10. Strategy for quantifying TSS-TES E box contacts in murine fibroblasts and list of qPCR primers used for this purpose.

Each circle shows the expected structure and size (in bps) of loops formed between TSS- and TES-proximal regions (green and brown thick lines, respectively) following DpnII digestion of formaldehyde cross-linked genomic DNA and re-ligation. Locations of ligated DpnII sites and E boxes (or their previous locations) are indicated. Sequences of PCR primers used to amplify ligated regions flanking the sites of DpnII digestion/re-ligation are indicated as are the predicted sizes of the amplified DNA products.

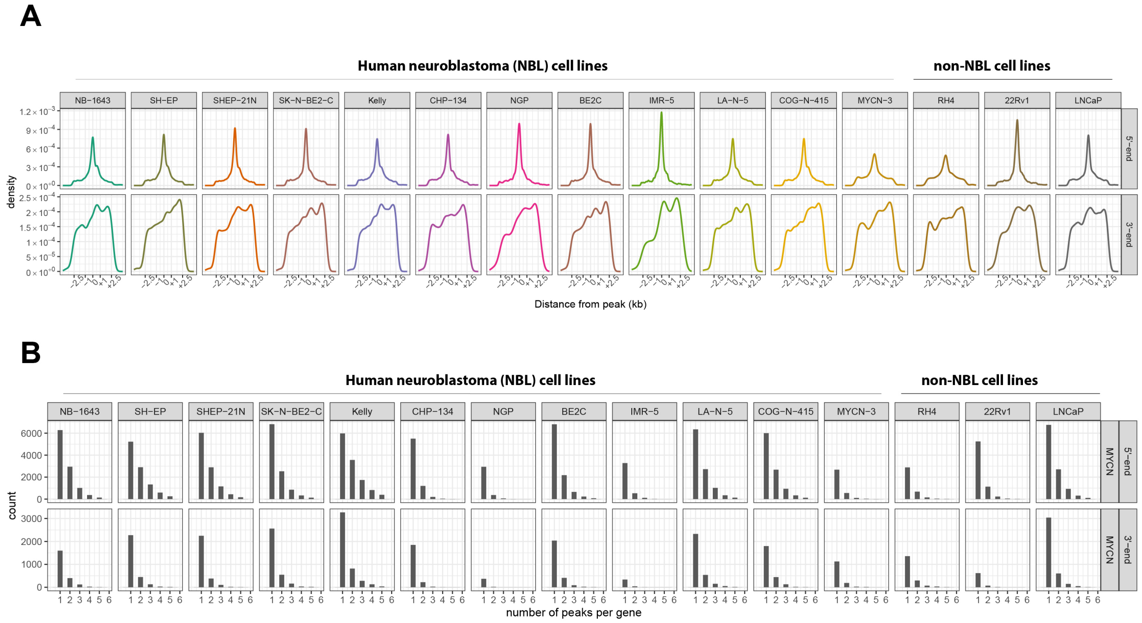

Fig. S11. Binding and distribution of MYCN to TSS- and TES-associated regions in its target genes.

(A). ChIPseq data for the indicated 9 neuroblastoma and 3 non-neuroblastma cell lines were retrieved from the ReMap2022 database and plotted as described in Fig. 1B. The distribution of MYCN binding sites relative to TSSs and TESs, which are defined as residing at position "0", is shown. Some genes are repeated to allow for assignments to multiple TSSs and/or TESs

(B). Distribution of MYCN binding sites residing within +/- 2.5 kb of TSSs and TESs. These were plotted as described in Figure S1

File S1. Significantly enriched GO pathways corresponding to Supplementary Figure S8. This file lists the significantly enriched Gene Ontology (GO) pathways identified in the analysis shown in Supplementary Figure S8. Only pathways that passed the statistical significance threshold (adjusted p-value) are included.

File S2. Rank of 1210 TFs and transcriptional co-factors and the frequency with which they bound to MYC-associated TSSs, TESs and intragenic and distal enhancer elements. The ReMap2022 database (https://remap2022.univ-amu.fr/) was used a source of binding profile.

File S3. List of gene pairs and their expression levels from Figure 8.
